# Genome-wide analysis and expression profiles of glyoxalase gene families in Chinese cabbage (*Brassica rapa* L)

**DOI:** 10.1371/journal.pone.0191159

**Published:** 2018-01-11

**Authors:** Guixin Yan, Xin Xiao, Nian Wang, Fugui Zhang, Guizhen Gao, Kun Xu, Biyun Chen, Jiangwei Qiao, Xiaoming Wu

**Affiliations:** Oil Crops Research Institute of the Chinese Academy of Agricultural Sciences, Key Laboratory of Biology and Genetic Improvement of Oil Crops, Ministry of Agriculture, Wuhan, P. R. China; Huazhong University of Science and Technology, CHINA

## Abstract

The glyoxalase pathway is composed of glyoxalase I (GLYI) and glyoxalase II (GLYII) and is responsible for the detoxification of a cytotoxic metabolite methylglyoxal (MG) into the nontoxic S-D-lactoylglutathione. The two glyoxalase enzymes play a crucial role in stress tolerance in various plant species. Recently, the *GLY* gene families have well been analyzed in Arabidopsis, rice and soybean, however, little is known about them in Chinese cabbage (*Brassica rapa*). Here, 16 *BrGLYI* and 15 *BrGLYII* genes were identified in the *B*. *rapa* genome, and the BrGLYI and BrGLYII proteins were both clustered into five subfamilies. The classifications, chromosomal distributions, gene duplications, exon–intron structures, localizations, conserved motifs and promoter *cis*-elements were also predicted and analyzed. In addition, the expression pattern of these genes in different tissues and their response to biotic and abiotic stresses were analyzed using publicly available data and a quantitative real-time PCR analysis (RT-qPCR). The results indicated that the expression profiles of *BrGLY* genes varied among different tissues. Notably, a number of *BrGLY* genes showed responses to biotic and abiotic stress treatments, including *Plasmodiophora brassicae* infection and various heavy metal stresses. Taken together, this study identifies *BrGLYI* and *BrGLYII* gene families in *B*. *rapa* and offers insight into their roles in plant development and stress resistance, especially in heavy metal stress tolerance and pathogen resistance.

## Introduction

The glyoxalase system is a ubiquitous pathway in all organisms that consists of the following two enzymes: glyoxalase I (GLYI) and glyoxalase II (GLYII). The major function of this pathway is the detoxification of the potent cytotoxin methylglyoxal (MG) into D-lactate through two sequential reactions [1]. GLYI catalyzes the conversion of MG into S-D-lactoylglutathione (S-LG) with glutathione (GSH). GLYII catalyzes S-LG to yield D-lactate and replenishes the GSH that was consumed in the GLYI reaction step. The functions of the glyoxalases have been studied in animals and microbial systems (Thornalley, 1990). However, only several *GLYI* and *GLYII* genes have been cloned in plants, including the *GLYI* gene in *Brassica napus* [2], *Brassica juncea* [3], *Brassica oleracea* [4], *Lycopersicon esculentum* [5], *Glycine max* [6], *Oryza sativa* [7], *Sporobolus stapfianus* [4], *Thlaspi caerulescens* [8], *Triticum aestivum* [9], and *Vigna radiata* [10] and the *GLYII* gene in *Aloe vera* [11], *A*. *thaliana* [12], *B*. *juncea* [13], *Oryza sativa* [14], and *Spinacia oleracea* [15].

Previous studies have found a firm link between the GLY enzymes and stress tolerance in plants. GLYI activity and transcripts can be up-regulated under various stress treatments in different plants [5, 16–18]. The transcription and protein expression level of *GLYI* in tomato was up-regulated in response to salinity stress and phytohormonal and osmotic stimulation [5]. In pumpkin seedlings, *GLYI* transcripts were induced by salinity, heavy metal, white light, and MG treatments [19]. The up-regulation of GLYI and GLYII activity in onions was observed in response to drought and low temperature stress [20]. *B*. *juncea GLYII* can be up-regulated by salt and heavy metal treatments and ABA stress [13]. Therefore, the glyoxalases have been proposed to be potential markers associated with plant stress responses [21].

Furthermore, transgenic tobacco that overexpressed the *B*. *juncea GLYI* gene (*BjGLYI*) conferred an enhanced resistance to high concentration of MG and salinity [3, 22]. Overexpressing the same *GLYI* gene in *V*. *mungo* imparted salt stress tolerance to transgenic tobacco [23]. Tobacco overexpressing *GLYI* from wheat (*T*. *aestivum* L.) showed an enhanced tolerance to ZnCl_2_ stress [9]. In our recent study, yeast cells transformed with *B*. *napus GLYI* showed an improved tolerance to heat and cold stresses [2]. Tobacco and even rice overexpressing the rice *GLYII* gene showed an improved tolerance to high MG and salt conditions [22, 24] Consistent with the above-mentioned results, the overexpression of *GLYII* gene in *B*. *juncea* imparted an improved tolerance to salt stress [25]. Furthermore, *GLYII* transgenic tobacco sustained growth and yielded viable seeds in soils treated by ZnCl_2_ [26]. *A*. *thaliana* overexpressing the *GLYII* gene had an improved tolerance to salt and anoxia stress [27]. Transgenic tobacco overexpressing the *Brassica GLYI* and rice *GLYII* genes showed an increased tolerance to salinity and heavy metal stress than the wild type plants [22, 26]. Recently, the overexpression of glyoxalase system genes (*B*. *juncea*, *BjGLYI*, and *Pennisetum glaucum*, *PgGLYII*) enabled the Carrizo citrange rootstock to tolerate to salt stress, which provided a useful biotechnological method of resisting abiotic stress for woody plant. In conclusion, the overexpression of glyoxalases in plants via genetic manipulation can successfully improve stress tolerance (**Table 1**).

**Table 1:** Summary of the known functions of the *GLYI / GLYII* genes using a transgenic approach.

Species	Gene name	Accession number	Host	Functions	Reference
Sugar beet	*GLYI*	gi15220397	Tobacco	Tolerance to MG, salt, mannitol and H_2_O_2_	[28]
*B*. *juncea*	*GLYI*	Y13239	Tobacco	Tolerance to MG and salt	[3, 22]
*B*. *juncea*	*GLYI*	Y13239	Blackgram	Alleviation of salt stress	[23]
*B*. *juncea*	*GLYI*	Y13239	*Arabidopsis*	Salinity tolerance	[29]
*B*. *juncea*	*GLYI*	Y13239	Rice	Salinity tolerance	[30]
Wheat	*GLYI*	ES451795	Tobacco	Tolerance to Heavy metals	[9]
*B*. *napus*	*GLYI*	KT720495	Yeast	Thermotolerance and cold tolerance	[2]
Rice	*GLYI*	AK108253	Rice	tolerance to NaCl, ZnCl_2_ and mannitol	[31]
Rice	*GLYII*	AY054407	Tobacco	Salinity tolerance	[22]
Rice	*GLYII*	AY054407	rice	Tolerance against MG and salt	[24]
*A*. *thaliana*	*GLYII*	AT2g43430	*A*. *thaliana*	Tolerance against Anoxia, salt stress	[27]
*B*. *juncea* and Rice	*BjGLYI*, *OsGLYII*	Y13239, AY054407	Tobacco	Heavy metals and salinity tolerance	[26]
*B*. *juncea*, and *P*. *glaucum*	*BjGLYI*, *PgGLYII*	Y13239, AF508863.1	Carrizo Citrange	Salinity tolerance	[32]
*B*. *juncea*, and *P*. *glaucum*	*BjGLYI*, *PgGLYII*	Y13239, AF508863.1	Tomato	Salinity tolerance	[33]

*GLYI* and *GLYII* belong to the glyoxalase family. To date, a genome-wide analysis has revealed that there are 11 *GLYI* in both *Arabidopsis* and rice and there are five and three *GLYII* in *Arabidopsis* and rice, respectively [34]. Recently, the release of the *B*. *rapa* genome sequence [35] facilitated the identification and systematic analysis of the putative glyoxalase genes across the whole genome in *B*. *rapa* L (a model organism representing the *Brassica* species). In our study, we characterized 16 *BrGLYI* and 15 *BrGLYII* genes based on a sequence analysis. Detailed information regarding the classification, chromosomal distribution, gene duplication, exon–intron structure, localizations, phylogenetic tree, conserved motif, and promoter *cis*-elements of the genes were predicted and analyzed. Their expression in different organs and under biotic and abiotic stresses was also discussed. This study provides a clearer understanding of the function of the genes in *Brassicas* and promotes further study in other organisms.

## Methods

### Materials and stress treatments

The *B*. *rapa* cultivar Chiffu was planted in a growth chamber at the Oil Crops Research Institute of the Chinese Academy of Agricultural Sciences in Wuhan at 20 ± 2°C with 12 h light and 12 h dark. The roots were sampled from young seedlings. Fresh flower buds were obtained, and the other tissues were sampled approximately 25 day after flowering, including stems, leaves, siliques and seeds. Three biological replicates of each tested tissue were prepared by harvesting samples from three different individuals. The samples were quickly frozen in liquid nitrogen and stored at -80°C until RNA isolation.

A *B*. *rapa* landrace (Wuxianzangcaizi) was used for the heavy metal treatment. Healthy seeds of similar sizes were surface-sterilized, dried and then germinated in sterilized moist filter paper. The seeds were treated with fresh medium supplemented with 20 mL 15 mg/L CdCl_2_ or 50 mg/L Pb (NO3)_2_ [36]. Seeds treated with an equal amount of distilled water served as controls. Three replicates of 50 seeds were used for each treatment. The treatment and control seeds were cultured in darkness for 24 h at 22°C and then cultured during the photoperiod (16 h light /8 h dark cycle) for seven days. The shoots and roots from seedlings of similar sizes were harvested separately and washed three times with deionized water. The samples were frozen in liquid nitrogen until the RNA extraction.

### Identification and analysis of glyoxalase proteins in *B*. *rapa*

Pfam (http://pfam.sanger.ac.uk/) accessions PF00903 for GLYI and PF00753 for GLYII were used for a Hidden Markov Model (HMM) search [34]. The whole genomic sequence of *B*. *rapa* was obtained from the *Brassica* database (BRAD, http://brassicadb.org/brad/) [35]. The *GLYI* and *GLYII* genes were extracted from the whole genomic sequence according to the descriptions provided by Wang et al. [37].

### Analyses of chromosomal locations, gene structures, and gene duplications in the *BrGLYI* and *BrGLYII* genes

The genomic positions of the *BrGLYI* and *BrGLYII* genes on *B*. *rapa* chromosomes were analyzed using a BLASTn search. The *BrGLYI* and *BrGLYII* gene structures were analyzed using the Gene Structure Display Server Program (GSDS, http://gsds.cbi.pku.edu.cn/index.php) [38]. Duplication of *BrGLYI* and *BrGLYII* and their positions were compared between the *Arabidopsis* and *B*. *rapa* subgenomes as previously described [39].

### Sequences analysis and construction of the phylogenetic tree

Clustal X software (ftp://ftp-igbrmc.u-strasbg.fr/pub/clustalX/) was used for amino acid (aa) alignments. Phylogenetic analysis was constructed with the MEGA 5.05 software using the neighbor-joining (NJ) method and 1,000 bootstrap tests [40].

### Sub-cellular localization of the predicted GLYI proteins

The sub-cellular localizations of all predicted BrGLYI and BrGLYII proteins were analyzed using different online tools, i.e., Wolf pSORT [41], TargeP, and ChloroP [42].

### Promoter sequence analysis

To analyze the regulatory elements in the *BrGLYI* and *BrGLYII* promoters, the 1.5 kb 5’-upstream sequences from the ATG initiation code were obtained from BRAD, and analyzed using PlantCARE databases (http://bioinformatics.psb.ugent.be/webtools/plantcare/html/) [40, 43].

### Gene expression analysis

The *BrGLY* expression in root, stem, leaf, flower and silique tissues from 7-week-old and callus Chinese cabbage (Chiifu-401-42) were analyzed using the transcriptomes data online (http://www.ncbi.nlm.nih.gov/geo/query/acc.cgi?acc=GSE43245) [44]. The data were used to generate a heatmap using the Heat map Illustrator (HemI, http://hemi.biocuckoo.org/down.php) package [45].

To reveal the response of the *BrGLY* genes to biotic stress in Chinese cabbage, the expression of all *BrGLYI* and *BrGLYII* genes in response to pathogen infection was analyzed using the reported RNA-seq data (http://www.ncbi.nlm.nih.gov/geo/query/acc.cgi?acc=GSE74044) [46].

The raw data obtained using the tag-based transcriptome sequencing approach were used to confirm the response of the *BrGLY* genes to the heavy metal stress, which was accessible through the GEO database (http://www.ncbi.nlm.nih.gov/geo/query/acc.cgi?acc=GSE55264) [47].

### RT-qPCR analyses

Total RNA was isolated using an isolation kit (BioTeke, RP3201). The cDNA was synthesized using a synthesis kit (TransGen Biotech), and the RT-qPCR was carried out as descried by Li et al. [39]. The relative expression of *BrGLY* was analyzed with the *Actin* as a housekeeping gene using a previously described method [48]. The specific primers designed are listed in **S1 Table.**

## Results

### Identification of the *GLYI/GLYII* genes in *B*. *rapa*

According to *B*. *rapa* genome sequence, we identified the GLY proteins in *B*. *rapa*. Proteins that contained the glyoxalase domain (Pfam databases, PF00903) and had a putative lactoylglutathione lyase function were classified as BrGLYI proteins. Likewise, proteins that contained the metallo-beta-lactamase domain (Pfam databases, PF00753) and had a putative hydroxyacyl glutathione hydrolase function were classified as BrGLYII proteins. In *B*. *rapa*, 16 *BrGLYI* and 15 *BrGLYII* genes were identified. The coding sequences and amino acid sequences of *BrGLY* genes were shown in S2 and S3 Tables.

### Detailed information for the identified *BrGLYI* and *BrGLYII* genes

We analyzed all the identified *BrGLYI* and *BrGLYII* genes in detail. The chromosomal locations, orientation, DNA length, exons and introns, coding DNA sequence (CDS) length, polypeptide (PP) length and isoelectric point (pI) of each *BrGLY* gene are shown in **Table 2.** The full DNA sequence length of *BrGLYI* varied from 555 bp (*BrGLYI8*) to 8430 bp (*BrGLYI6*), and their CDS length varied from 414 bp (*BrGLYI2* and *BrGLYI13*) to 3393 bp (*BrGLYI6*). Accordingly, *BrGLYI6* encodes the largest protein of the family (1131 aa, 125.1 kDa), and *BrGLYI2* and *BrGLYI13* encode the smallest protein (137 aa, 15.24 kDa) (**Table 2**). In addition, the proteins showed a large variation in pI value from 4.78 (BrGLYI8) to 8.59 (BrGLYI6). Most of the BrGLYI proteins were acidic, and only four proteins (i.e., BrGLYI2, BrGLYI4, BrGLYI5 and BrGLYI6) showed a basic pI value (**Table 2**). Most of the BrGLYI proteins were localized in the chloroplast, followed by the cytosol, nucleus and mitochondria (**Table 2**).

**Table 2:** List of putative *BrGLYI* and *BrGLYII* genes along with gene and protein detailed information.

Gene symbol	Location	Locus identifier	Gene Start (bp)	Gene Stop (bp)	Strand	DNA Length (bp)	No. of Introns	CDS length (bp)	PP length (aa)	MW (kDa)	pI	Localization
*BrGLYΙ1*	A02	Bra008491	14958779	14959587	-	809	2	504	167	18.79	5.82	Ch^a^, Nu^a^, Cy^b^
*BrGLYΙ2*	A03	Bra006835	5272539	5273434	+	896	3	582	193	21.8	8.46	Ch^a^, Mt^a^^b^
*BrGLYΙ3*	A05	Bra005612	6530309	6531239	-	931	3	414	137	15.24	5.47	Nu^a^, Cy^b^
*BrGLYΙ4*	A06	Bra018692	2596401	2597542	-	1142	3	516	171	19.27	7.94	Ch^a^^b^
*BrGLYΙ5*	A06	Bra018654	2805621	2807386	+	1766	7	714	238	26.7	8.37	Ch^a^^b^^c^, Mt^b^
*BrGLYΙ6*	A06	Bra019830	4121249	4129678	+	8430	23	3393	1131	125.1	8.59	Cy^a^^b^
*BrGLYΙ7*	A06	Bra026138	5615367	5616250	+	884	2	525	174	19.78	5.69	Ch^a^, Nu^a^, Cy^b^
*BrGLYΙ8*	A07	Bra011950	13267717	13268271	+	555	0	555	184	20.89	4.78	Cy^a^, Nu^b^
*BrGLYΙ9*	A07	Bra004214	20748112	20749979	-	1868	8	1026	342	38.0	6.21	Cy^a^, Ch^b^^c^
*BrGLYΙ10*	A08	Bra016662	18698136	18699063	-	928	2	525	174	19.81	5.89	Ch^a^, Cy^b^
*BrGLYΙ11*	A08	Bra016811	19309787	19311275	-	1489	7	852	284	31.9	5.26	Ch^a^, Nu^a^, Cy^b^
*BrGLYΙ12*	A09	Bra026768	35370602	35371857	-	1256	2	504	167	18.94	6.87	Ch^a^, Cy^b^
*BrGLYΙ13*	A09	Bra031589	37436032	37436826	+	795	2	414	137	15.39	5.85	Ch^a^, Cy^b^
*BrGLYΙ14*	A09	Bra032415	37731431	37732580	+	1150	1	1083	361	39.3	5.07	Ch^a^^b^^c^, Cy^b^
*BrGLYΙ15*	A10	Bra015511	847827	849238	+	1412	1	1332	444	48.9	5.60	Ch^a^^b^, Cy^b^
*BrGLYΙ16*	A10	Bra002767	7787942	7788921	-	980	3	588	195	22.05	6.48	Mt^a^^b^
*BrGLYII1*	A01	Bra011454	2103079	2107882	-	4804	18	1905	635	71.5	6.10	Cy^a^^b^
*BrGLYII2*	A01	Bra031460	17101371	17103984	-	2614	4	2085	695	77.6	5.81	Nu^a^, Mt^a^, Cy^a^^b^
*BrGLYII3*	A02	Bra026637	19722680	19725677	+	2998	12	1848	616	68.3	6.76	Cy^a^
*BrGLYII4*	A03	Bra022836	7342311	7344180	+	1870	7	984	328	36.1	8.86	Ch^a^^c^, Mt^b^
*BrGLYII5*	A03	Bra000305	10460100	10461894	-	1795	6	861	287	31.7	7.81	Nu^a^, Mt^a^^b^
*BrGLYII6*	A04	Bra037715	18205706	18207328	-	1623	6	846	282	31.1	7.82	Mt^a^^b^, Ch^a^
*BrGLYII7*	A05	Bra004763	1717350	1719062	-	1713	6	861	287	31.9	8.54	Mt^a^^b^, Ch^a^,Nu^a^
*BrGLYII8*	A05	Bra018252	6955516	6956948	-	1433	6	864	288	31.7	6.27	Mt^a^^b^
*BrGLYII9*	A05	Bra029872	22646169	22647629	+	1461	6	777	258	28.7	5.66	Cy^a^^b^
*BrGLYII10*	A06	Bra039681	572872	574388	+	1517	6	735	245	26.7	5.80	Mt^a^^b^
*BrGLYII11*	A06	Bra038629	14869488	14874010	+	4523	15	2724	908	100.1	8.53	Ch^a^^b^^c^, Nu^b^
*BrGLYII12*	A06	Bra009712	17316320	17321154	+	4835	16	2220	740	82.0	5.14	Ch^a^, Cy^a^^b^
*BrGLYII13*	A08	Bra030931	898468	900230	+	1763	7	729	243	26.4	6.14	Nu^a^^b^, Cy^b^, Mt^b^
*BrGLYII14*	A09	Bra024757	24197064	24199869	-	2806	11	1566	522	57.6	6.07	Ch^a^, Cy^b^
*BrGLYII15*	A09	Bra032436	37823442	37825415	+	1974	7	984	328	35.7	6.89	Ch^a^^b^^c^, Mt^b^

Abbreviations: CDS: coding DNA sequence, PP: polypeptide length, MW: molecular weight, PI: isoelectric point, bp: base pair, aa: amino acid, kDa: kilodalton, Ch: chloroplast, Cy: cytosol, Mt: mitochondria, Nu: nucleus

a Localization prediction by pSORT (http://www.genscript.com/wolf-psort.html)

b Localization prediction by TargetP 1.1 Server (http://www.cbs.dtu.dk/services/TargetP/)

c Chloroplast localization signal confirmed by ChloroP (http://www.cbs.dtu.dk/services/ChloroP/)

Similarly, the full DNA sequence length of *BrGLYII* varied from 1433 bp (*BrGLYII8*) to 4835 bp (*BrGLYII12*), and the CDS length of *BrGLYII* varies from 729 bp (*BrGLYII13*) to 2724 bp (*BrGLYII11*) (**Table 2**). The largest protein (908 aa, 100.1 KDa) of the *BrGLYII* family is encoded by *BrGLYII11*, and the smallest protein is BrGLYII13 (243 aa, 26.4 kDa) (**Table 2**). BrGLYII proteins also showed a deviation in pI values, which varied from 5.14 (BrGLYII12) to 8.86 (BrGLYII4). Overall, 10 of the 15 BrGLYII proteins showed an acidic pI value, while only five showed a basic pI value. These results are similar to those obtained for the BrGLYI proteins. The localization analysis indicated that BrGLYII proteins localized more in the mitochondria than at the other sites, such as the chloroplast, cytosol and nucleus (**Table 2**).

### Chromosomal distribution of the *BrGLYI* and *BrGLYII* genes

**Fig 1:**
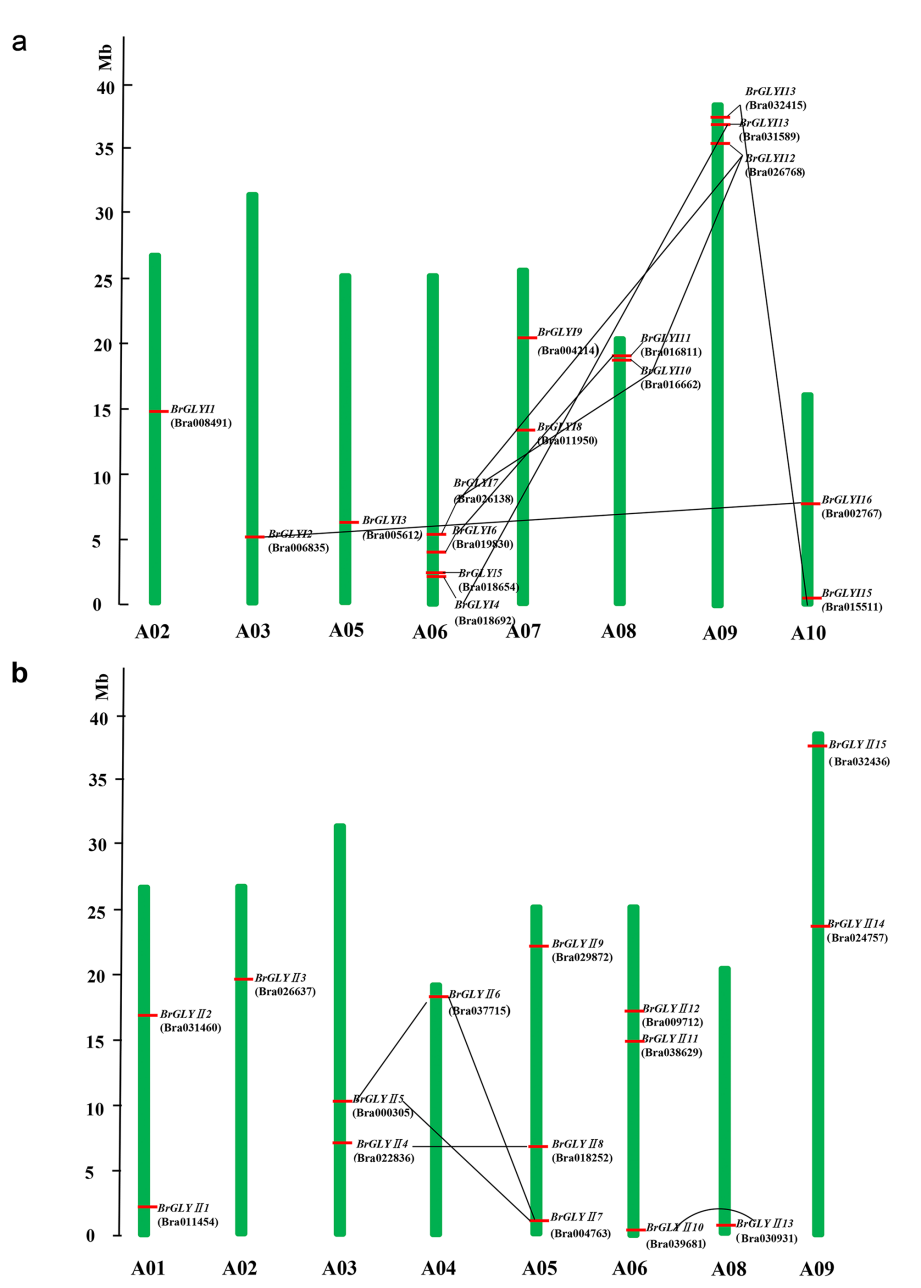
**The positions of the *BrGLYI* (A) and *BrGLYII* (B) genes distributed on *B*. *rapa* chromosomes.** Duplicated glyoxalase genes are connected by black lines between the two relevant chromosomes. The scale is in megabase (Mb). The exact position (Mb) of each glyoxalase gene is shown on the chromosomes. Chromosome numbers are shown at the bottom of each bar.

**Fig 1** shows the distribution of the *BrGLY* genes on *B*. *rapa* chromosomes. Regarding the chromosomal distribution of the *BrGLYI* genes, 16 genes are located on eight different chromosomes, which is highly uneven (**Fig 1A**). Chromosome 6 harbored the most *BrGLYI* genes (four *BrGLYI* genes). Chromosome 9 contained three *BrGLYI* genes, which is ranked second. Two *BrGLYI* genes are located on chromosomes 7, 8, and 10, and one *BrGLYI* gene is located on chromosomes 2, 3, and 5 (**Fig 1A and Table 2**). No *BrGLYI* genes were found on chromosomes 1 and 4. Regarding the *BrGLYII* genes, there were three genes on chromosomes 5 and 6. Two genes were identified on chromosomes 1, 3 and 9, while chromosomes 2, 4, and 8 harbored one *BrGLYII* gene each (**Fig 1B and Table 2**). No *BrGLYII* genes were present on chromosomes 7 and 10.

Duplication events have been previously studied in most plant species. Among the BrGLYI proteins, nine duplicated genes, which shared relatively high sequence similarities (aa identity >90%), were identified in the *B*. *rapa* genome (**Fig 1A**, **S4 Table**). Three of the duplicated genes were categorized into one group (*BrGLYI10*/*BrGLYI12*/*BrGLYI7*) that exhibited a high sequence similarity (>95%). The other six duplicated genes were divided into three groups (*BrGLYI15*/*BrGLYI14*, *BrGLYI4*/*BrGLYI13*, *BrGLYI2*), each of which contained only two duplicated genes. Three duplicated genes were located on chromosome 9, and two of these duplicated genes were distributed on chromosomes 6 and 10. Chromosomes 3 and 8 had one duplicated gene (**Fig 1A**).

There are seven duplicated *BrGLYII* genes in the *B*. *rapa* genome. The duplicated genes were divided into three groups, and the aa similarity of the genes in a group was above 95%. One gene group contained three genes (*BrGLYII7*/*BrGLYII6*/*BrGLYII5*), and the other two groups contained two genes (*BrGLYII13*/*BrGLYII10*, *BrGLYII8*/*BrGLYII4*). Chromosome 3 had two duplicated genes, and chromosomes 4, 5, 6 and 8 contained one duplicated gene **(Fig 1B, S5 Table**).

Additionally, by comparing the *GLYI* and *GLYII* genes between *Arabidopsis* and the *B*. *rapa* subgenomes, we found that there are seven *BrGLYI* genes in least fractionated blocks (LF), four *BrGLYI* genes are located in the medium fractionated blocks (MF1), and two *BrGLYI* genes are located in the most fractionated blocks (MF2) (**Table 3**). Two of the *BrGLYII* genes are located in the LF and MF2 blocks, and one gene is distributed in the MF1 blocks (**Table 3**). In addition, only one gene, the *AtGLYI4* gene, is triplicated; three genes, including *AtGLYI1*, *AtGLYI11* and *AtGLYII*, are duplicated in the subgenome of *B*. *rapa* (**Table 3**). There are no *Arabidopsis* genes that are homologous to *BrGLYI3*, *BrGLYI6* and *BrGLYI14*.

**Table 3:** Identification of homologous *GLYI* and *GLYII* genes between *A*. *thaliana* and subgenomes in *B*. *rapa*.

*A*. *thaliana*	Locus identifier	CCB^a^	LF^b^	MF1^c^	MF2^d^
AtGLYI1	AT1G07645	A	BrGLYI4	–	BrGLYI13
AtGLYI2	AT1G08110	A	BrGLYI5	–	–
AtGLYI3	AT1G11840	A	–	BrGLYI11	–
AtGLYI4	AT1G15380	A	BrGLYI7	BrGLYI10	BrGLYI12
AtGLYI5	AT1G64185	D	–	–	–
AtGLYI6	AT1G67280	E	BrGLYI9	–	–
AtGLYI7	AT1G80160	E	–	BrGLYI1	–
AtGLYI8	AT2G28420	I	BrGLYI8	–	–
AtGLYI9	AT2G32090	J	BrGLYI3	–	–
AtGLYI10	AT5G41650	S	–	–	–
AtGLYI11	AT5G57040	Wb	BrGLYI16	BrGLYI2	–
AtGLY12	AT1G06570	A	BrGLYI15	–	BrGLYI14
AtGLYII1	AT1G06130	A	–	–	BrGLYII15
AtGLYII2	AT1G53580	C	BrGLYII10	BrGLYII13	–
AtGLYII3	AT2G31350	J	BrGLYII8	–	BrGLYII4
AtGLYII4	AT3G10850	F	BrGLYII9	–	–

a, conserved collinear block

b, the least fractionated blocks of *B*. *rapa* subgenome

c, the medium fractionated blocks

d, the most fractionated blocks.

### Phylogenetic and structure analyses of the *BrGLYI* and *BrGLYII* gene families

**Fig 2:**
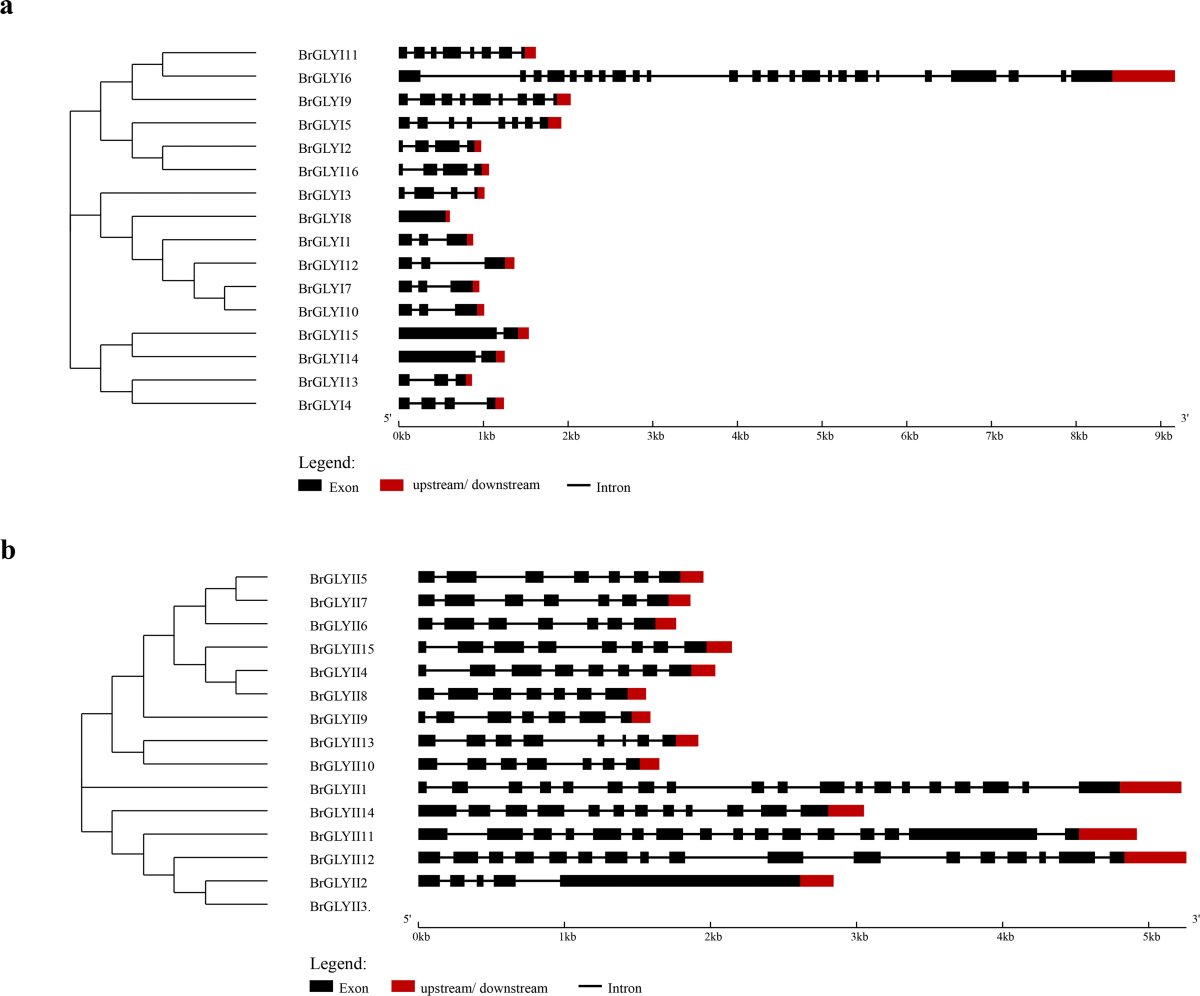
Gene structure and phylogenetic relationship of *BrGLYI* and *BrGLYII*. (A) BrGLYI, (B) BrGLYII proteins. An unrooted tree was generated using the Neighbor-Joining method with 1,000 bootstrap by MEGA5.05 software using the full-length amino acid sequences of the sixteen BrGLYI and fifteen BrGLYII proteins. CDS and amino acid sequences of *BrGLYI* and *BrGLYII* are listed in S2 and S3 Tables.

The gene structure analysis of the *BrGLYI* and *BrGLYII* indicated that the *BrGLY* genes had one to 23 introns except *BrGLYI8* (**Fig 2 and Table 2**). *BrGLYI13* and *BrGLYI14* only contained one intron. *BrGLYI2* gene had the largest number of introns. The *BrGLYII* genes also contained varied numbers of introns; for example, eighteen introns were identified in *BrGLYII1*, and four introns were predicted in *BrGLYII2*. As shown in Fig 2, the GLY proteins that clustered together possess a similar structure.

**Fig 3:**
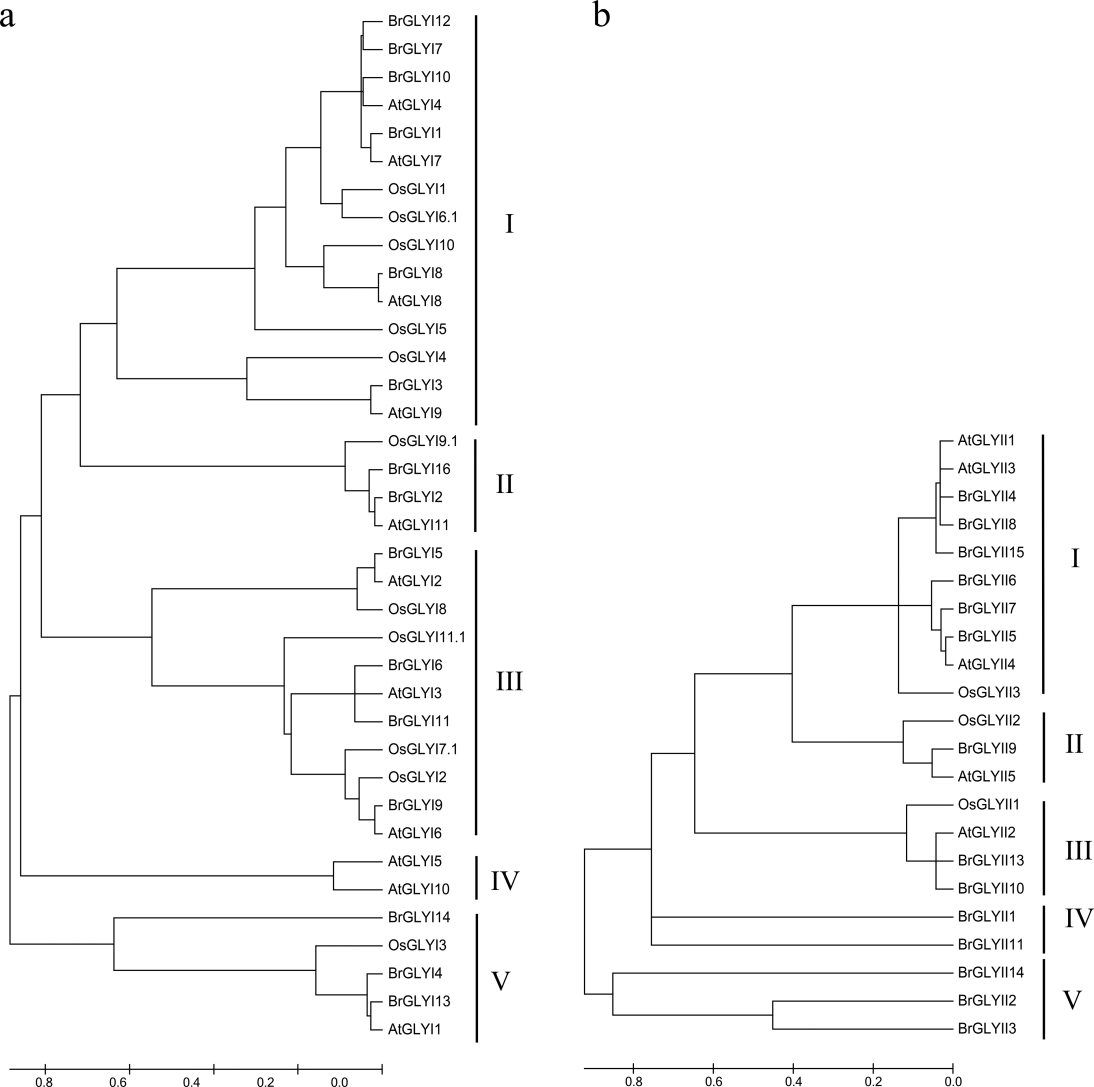
Phylogenetic relationships of GLYI and GLYII from various plant species. A phylogenetic tree based on the multiple alignments of the GLYI and GLYII amino acid sequences was constructed using MEGA 5.05 software with the Neighbor-Joining method. Bootstrap support from 1,000 reiterations is indicated above the branches. “Br”, “At” and “Os” refer to the GLYI and GLYII proteins in *B*. *rapa*, *A*. *thaliana* and *O sativa* (only the first splice variants were considered in the case of multiple members), respectively.

To examine the evolutionary relationships of the *GLY* genes among the predicted GLY proteins in Chinese cabbage, Arabidopsis and rice, a phylogenetic tree was drawn using their aa sequences. The results indicated that the GLYI and GLYII proteins were divided into five subfamilies (**Fig 3**). Among the GLYI proteins, the largest clade (Clade I) contained 15 members, whereas the smallest group (Clade IV) contained only two members from *Arabidopsis* (**Fig 3A**). The results indicated that the homology between BrGLYI and OsGLYI was much lower than that between BrGLYI and AtGLYI (**Fig 3A**). Clade I included six members of *B*. *rapa*, whereas four proteins were from Arabidopsis and five proteins were from rice. In this group, *AtGLYI4* transcription can be induced by osmotic, extreme temperature and wounding stress. Furthermore, *AtGLYI7* is highly up-regulated under salt, osmotic, extreme temperature and wounding stress [34]. Three BrGLYI proteins (BrGLYI7, BrGLYI10 and BrGLYI12) in Chinese cabbage had a high sequence similarity with AtGLYI4. BrGLYI1 showed a high similarity to AtGLYI7. We hypothesized that the similar BrGLYI proteins may play similar roles in the stress response. Group II contained one GLYI protein each in rice and Arabidopsis and two proteins in Chinese cabbage. Group III contained four BrGLYI proteins in Chinese cabbage. The functions of the proteins in this group may be related to salt stress because the OsGLYI 11 protein in this group improved the transgenic tobacco adaptation to lower Na^+^/K^+^ ratio stress [7]. Group IV only included two Arabidopsis proteins, i.e., the AtGLY5 and AtGLY10 proteins. Three BrGLYI proteins from Chinese cabbage, two GLYI proteins from rice and one protein from Arabidopsis belonged to Group V. OsGLYI3 in this group was found to be stress responsive (salinity stress, oxidative stress, and exogenous MG) in rice, which indicated its possible function in stress tolerance [34].

Similarly, the GLYII proteins formed five distinct clades (**Fig 3B**). Two BrGLYII proteins were clustered in groups III and IV, three BrGLYII proteins were classified in group V, and only one BrGLYII protein, BrGLYII9, was in Group II, whereas group I included six BrGLYII proteins (**Fig 3B**).

A phylogenetic relationship analysis revealed that GLYI and GLYII shared a closer relationship at the interspecific level, such as BrGLYI1, BjGLYI and BnGLYI. In addition, the proteins in Chinese cabbage showed a much closer evolutionary distance to *Arabidopsis* than rice; for example, BrGLYII9 displayed a closer relationship to AtGLYII5 than to OsGLYII (**Fig 3**).

To further analyze the protein sequence features of BrGLYI and BrGLYII, the conserved motifs of each protein were also identified using MEME (**S1 Fig**). We found that most proteins in the same group had similar motifs, and the LOGOs of these protein motifs were obtained by MEME (**S2 Fig**).

### Expression profiles of *BrGLYI* and *BrGLYII* in different tissues

**Fig 4:**
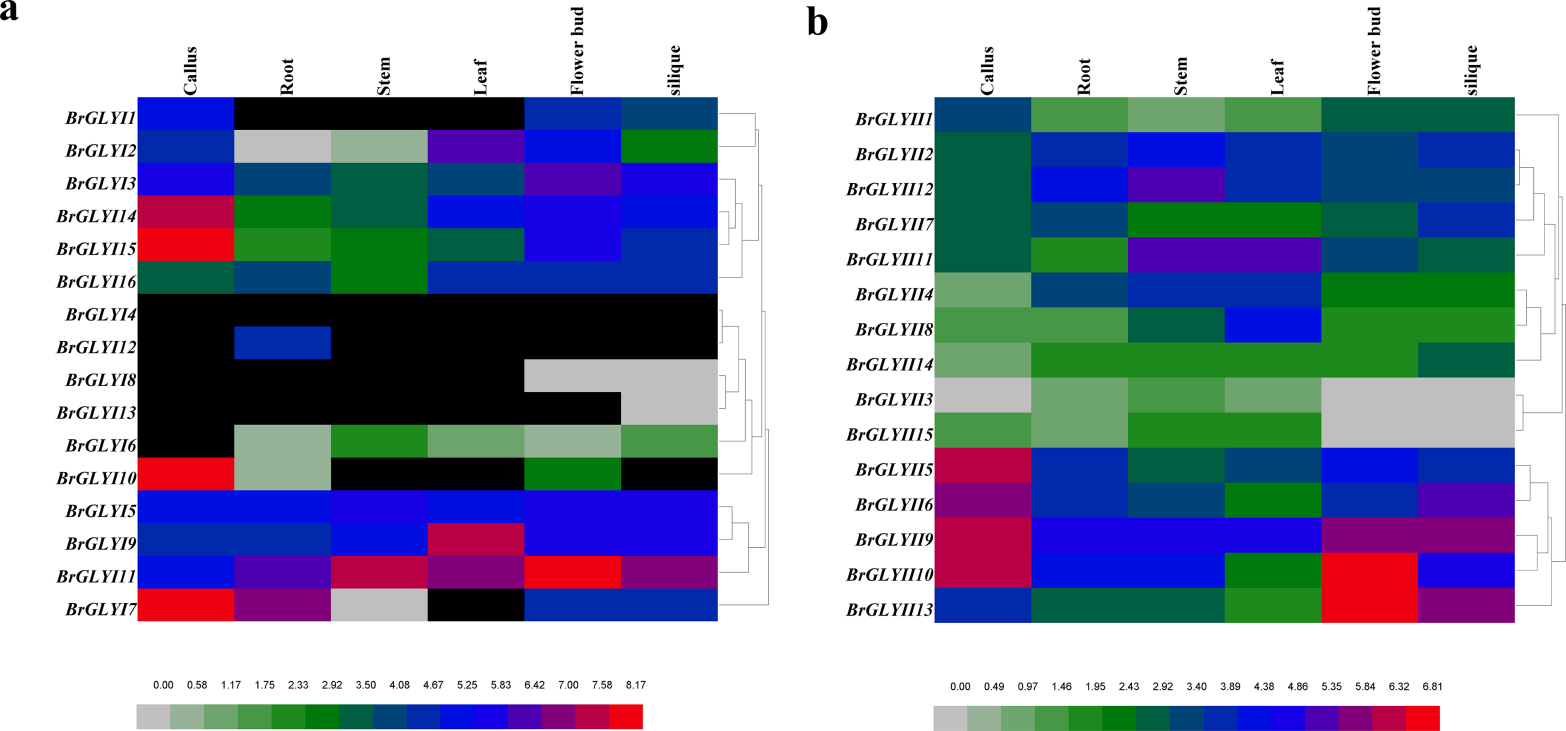
Expression profiles of the *BrGLY* genes using hierarchical clustering across various tissues. (A) The expression profiles of the *BrGLYI* genes; (B) The expression profiles of the *BrGLYII* genes. **Note:** The black color indicates that the gene was not detected in the tissue. The data was obtained from GEO database (http://www.ncbi.nlm.nih.gov/geo/query/acc.cgi?acc=GSE43245).

The transcription level of the *BrGLYI* and *BrGLYII* genes was analyzed using genome-wide transcription profiling data of Chinese cabbage (*B*. *rapa*). The expression data in roots, stems, leaves, flowers, siliques and callus were supplied. The Fragments Per Kilo base of exon sper Million fragments mapped (FPKM) values of the *BrGLY* gene are shown in **Fig 4** and **S6 Table**.

The expression clustering clearly reveals that the *BrGLYI* and *BrGLYII* genes were classified into different groups (**Fig 4**). By comparing the expression clustering data with the phylogeny analysis, we found that there was no direct correlation between the gene evolution and expression profiles. *BrGLYI3*, *BrGLYI5*, *BrGLYI9*, *BrGLYI11*, *BrGLYI14* and *BrGLYI15* showed a high level of ubiquitous expression during all developmental stages. Of the 16 *BrGLYI* genes, only the expression of *BrGLYI4* was undetectable during the six stages. *BrGLYI13* showed a very faint expression in siliques, whereas *BrGLYI8* showed a very faint expression in flowers and siliques. *BrGLYI6* showed a weak expression during all developmental stages, except for callus. Certain genes showed tissue-specific expression; for example, *BrGLYI12* was a root-specific gene. The expression of the other *BrGLYI* genes showed variable expression levels across different tissues. In contrast, the *BrGLYII* genes were all expressed in the six organs with variable expression levels; However, *BrGLYII3*, *BrGLYII14* and *BrGLYII15* were weakly expressed, and *BrGLYII1*, *BrGLYII4* and *BrGLYII7* showed a low expression level during all development stages (**Fig 4**).

**Fig 5:**
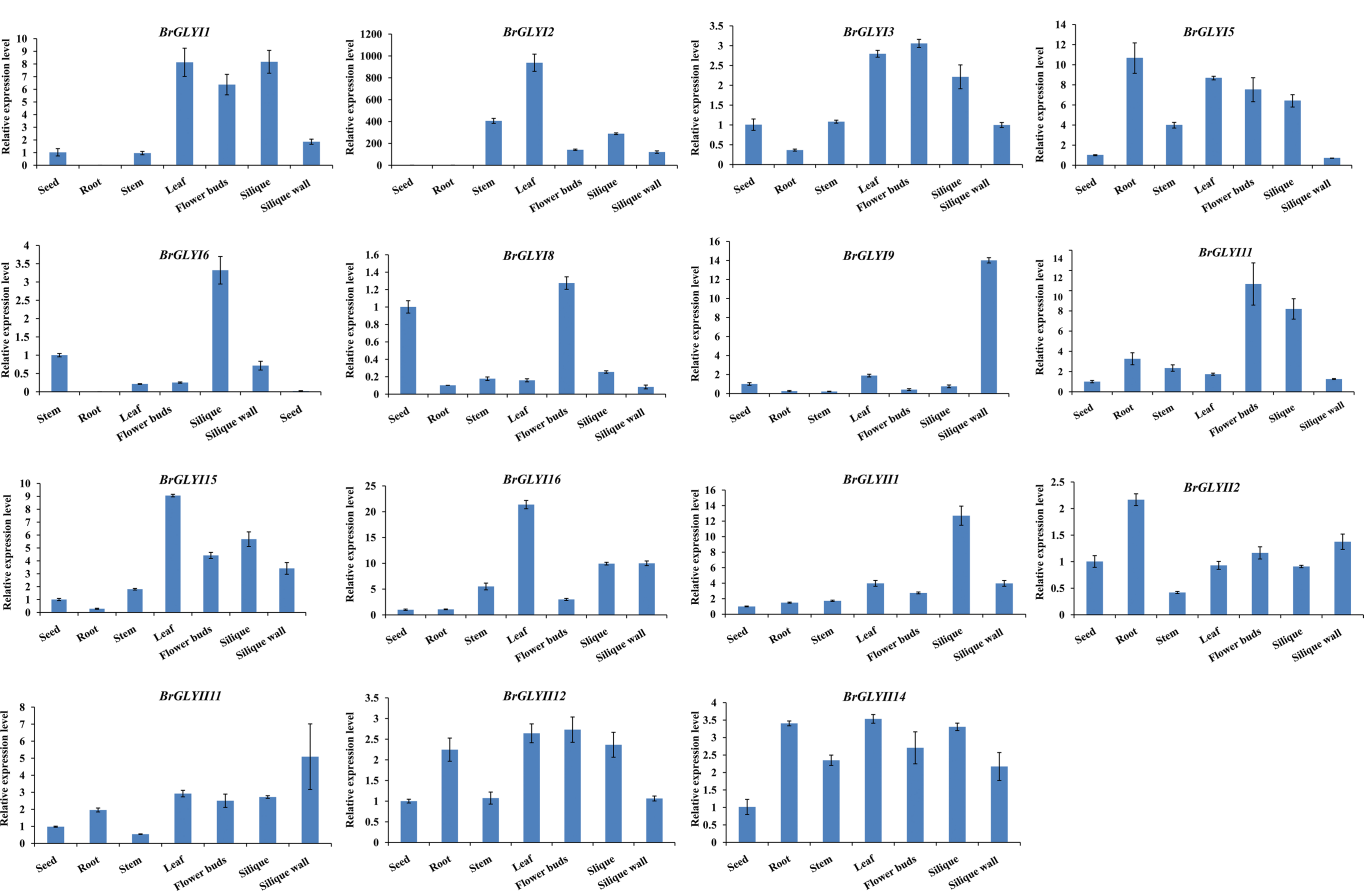
Relative expressions of the *BrGLYI* genes in different tissues of *B*. *rapa* confirmed by RT-qPCR. The normalized relative quantity in the seed was set as “1”. If the gene did not express in the seed, the expression level of in the stem was set at “1”.

To determine the expression patterns of the *BrGLYI* and *BrGLYII* genes obtained from the GEO data, we performed a RT-qPCR analysis of several genes from seven different organs (roots, stems, leaves, flower bud, siliques, silique wall and seeds) of *B*. *rapa*. After verifying the specificity for each primer pair, suitable RT-qPCR primer pairs for a total of 11 *BrGLYI* genes and 5 *BrGLYII* genes were selected (**S6 Table**). The expression of the other genes was not detected due to the unspecific primer design. The PCR products amplified ranged from 80 to 250 bp (**S6 Table**). According to the data, the expression pattern of the different *BrGLYI* and *BrGLYII* genes varied among the tissues (**Fig 5**). The expression of *BrGLYI4* was undetected, and the *BrGLYI8* and *BrGLYI6* genes were faintly expressed, which was consistent with the GEO data (**Fig 5**). *BrGLYI9* appeared to be expressed only in the root and stem, and *BrGLYI15* showed a lower expression level in the root. In addition, the expression of several genes could not be detected in certain tissues, e.g., the expression of *BrGLYI1*, *BrGLYI2* and *BrGLYI6* was not detected in the root (**Fig 5, S6 Table**). The above-mentioned results were consistent with the GEO data. The expression patterns of *BrGLYI2*, *BrGLYI3*, *BrGLYI9*, *BrGLYI11* and *BrGLYI14* were similar to the GEO data (**Fig 4** and **Fig 5**). However, several genes showed a lower or higher expression level in specific tissues, which was inconsistent with the GEO data. For example, *BrGLYI6* and *BrGLYI11* showed particularly high expression levels in the siliques, and *BrGLYI9* was strongly expressed in the silique wall; *BrGLYI5* and *BrGLYII2* were highly expressed in the root. *BrGLYI8* and *BrGLYI11* were much more highly expressed in the flower buds (**Fig 4** and **Fig 5**). Furthermore, the expression of *BrGLYI12* was obviously inconsistent with the public data due to its constitutive expression (**Fig 4** and **Fig 5**).

### Expression analysis of the *BrGLY* genes under stress conditions

**Fig 6:**
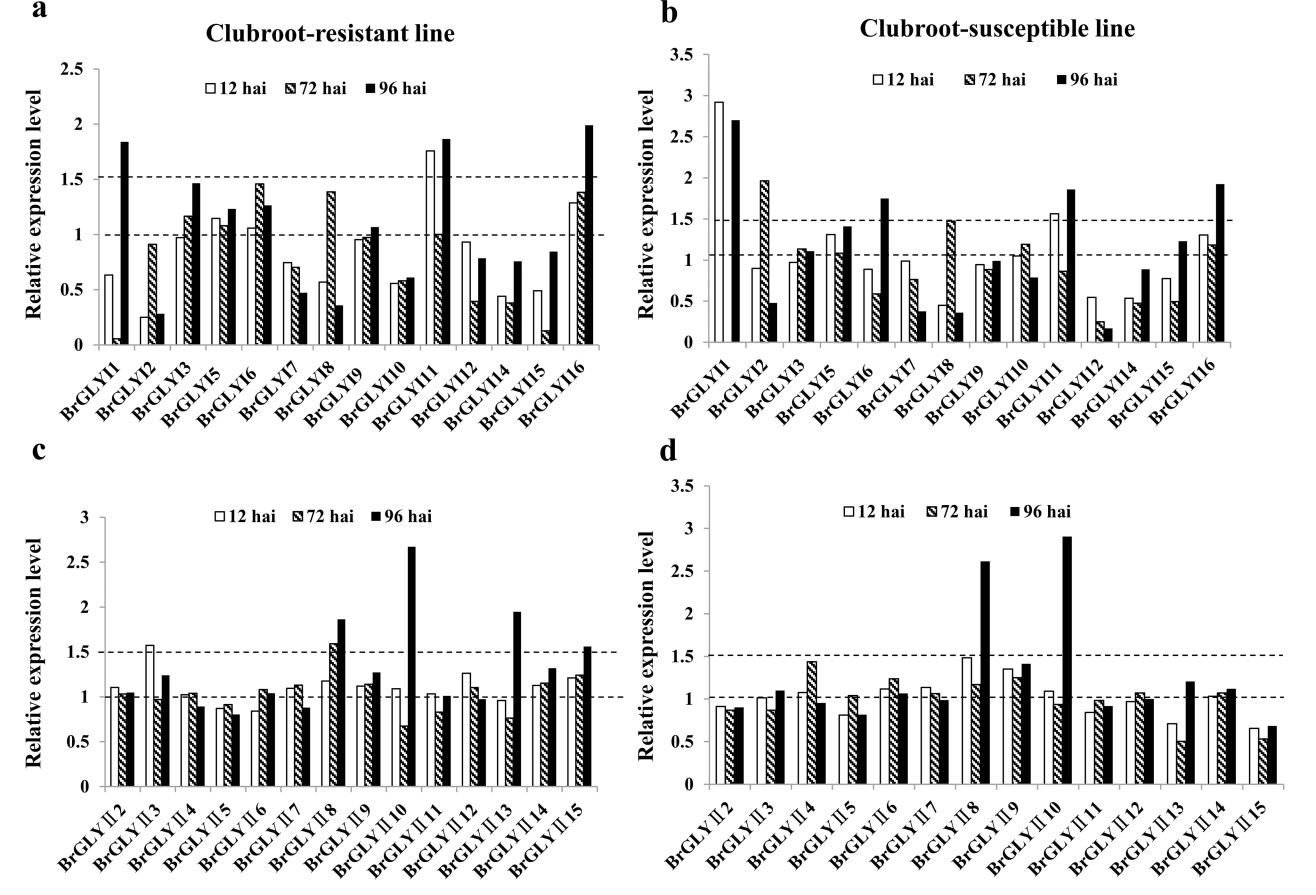
Expression of the *BrGLYI* genes in response to *P*. *brassicae* infection. Relative expression data of available *BrGLYI* (a, b) and *BrGLYII* (c, d) genes under heavy metal stresses and *P*. *brassicae* infection were obtained from the National Center for Biotechnology Information GEO database (http://www.ncbi.nlm.nih.gov/geo/query/acc.cgi?acc=GSE74044). Expression data is presented as fold-change by comparing with the corresponding samples under control conditions. a and c show the relative expression level at 12, 72, and 96 hours after inoculation (hai) in the near-isogenic lines (NILs) of the clubroot-resistant line of Chinese cabbage (*B*. *rapa*), while b and d show the expression level in the clubroot-susceptible line.

**Fig 7:**
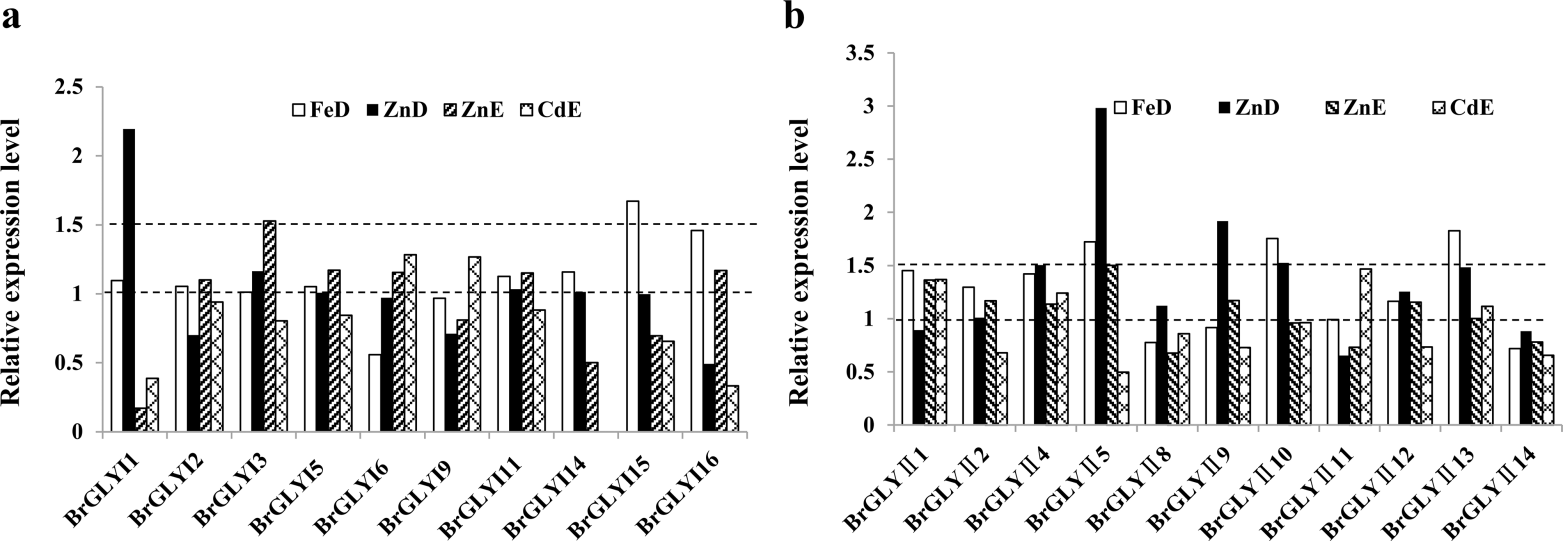
The relative expression levels of the *BrGLY* genes in response to heavy metal treatment. The raw data were obtained through GEO series accession number GSE55264 (http://www.ncbi.nlm.nih.gov/geo/query/acc.cgi?acc=GSE55264). The gene expression level changes under Zinc deficiency (ZnD), iron deficiency (FeD), excess Zn (ZnE) and cadmium exposure (CdE) compared to the normal condition were analyzed. (a) The *BrGLYI* gene expression level, (b) The *BrGLYII* gene expression level.

To reveal the response of the glyoxalase genes to biotic and abiotic stresses in Chinese cabbage, the expression of all *BrGLYI* and *BrGLYII* genes in response to stress conditions (including *P*. *brassicae* infection and FeD, ZnD, ZnE and CdE stress) were analyzed using the publicly available data regarding GSE74044 and GSE55264 in the GEO database. Among all *BrGLY* genes, 14 *BrGLYI* and *BrGLYII* genes were analyzed after *P*. *brassicae* infection, and 10 *BrGLYI* and 11 *BrGLYII* genes were analyzed under heavy metal stress. Different *BrGLY* genes showed diverse expression levels under these stresses (**Fig 6 and Fig 7**).

The *BrGLYI* genes in the two different lines showed relatively similar expression patterns (**Fig 6A and 6B**). In the clubroot-resistant line, four, five and seven *BrGLYI* genes showed an up-regulation at 12 hai, 72 hai and 96 hai after the *P*. *brassicae* infection, respectively. Among the up-regulated genes, the expression of *BrGLYI1*, *BrGLYI11* and *BrGLYI16* was up-regulated by more than 1.5-fold compared with their corresponding expression under the control condition. In the clubroot-susceptible line, four, five and seven *BrGLYI* genes showed an up-regulation at 12 hai, 72 hai and 96 hai after the *P*. *brassicae* infection, respectively. *BrGLYI1*, *BrGLYI6*, *BrGLYI11* and *BrGLYI16* were up-regulated by more than 1.5-fold at different infection times. Most interestingly, the *BrGLYI2* gene was down-regulated in the clubroot-resistant line; however, it was up-regulated in the clubroot-susceptible line.

In the case of *BrGLYII*, ten, eight and nine *BrGLYII* genes were induced in the clubroot-resistant line, while six, seven and seven *BrGLYII* genes were induced in the clubroot-susceptible line at 12 hai, 72 hai and 96 hai, respectively (**Fig 6C and 6D**). *BrGLYII8* and *BrGLYII10* were highly expressed after *P*. *brassicae* infection at 72 hai in both lines. *BrGLYII13* was more highly induced in the resistant line than susceptible line at 12 hai. Moreover, the expression of *BrGLYII15* was up-regulated in the resistant line; however, it was down-regulated in the susceptible line.

The expression of several *BrGLYI* genes was induced under the heavy metal stress conditions. FeD causes seven *BrGLYI* genes to become up-regulated and two *BrGLYI* genes to become down-regulated. Two *BrGLYI* genes were induced and four *BrGLYI* genes were down-regulated under the ZnD condition. Six *BrGLYI* genes were induced and four *BrGLYI* genes were down-regulated under the ZnE condition. The expression level of two *BrGLYI* genes increased and that of eight *BrGLYI* genes decreased under the CdE condition. Among these genes, *BrGLYI1* was induced by more than 2-fold under the ZnD condition, whereas *BrGLYI13* was induced by more than 1.5-fold under the ZnE condition. *BrGLYI15* was significantly induced under the FeD condition (**Fig 7A**).

By analyzing the response of the *BrGLYII* genes to the heavy metal stress, we found that the expression of *BrGLYII5*, *BrGLYII10* and *BrGLYII13* was significantly up-regulated (over 1.5-fold) under the FeD condition. *BrGLYII4*, *BrGLYII5*, *BrGLYII9*, *BrGLYII10* and *BrGLYII13* showed an up-regulation under the ZnD stress condition, whereas *BrGLYII5* showed a significant up-regulation under the ZnE stress condition. *BrGLYII11* was induced by approximately 1.5-fold under the CdE stress condition. These results illustrate the diverse responses of different *BrGLY* genes in the stress regulatory pathways in Chinese cabbage. Among the *BrGLYII* gene members, *BrGLYII5* was induced under the FeD, ZnD and ZnE stress conditions, which suggested that it may play a crucial role in heavy metal stress and its function requires further validation (**Fig 7B**).

**Fig 8:**
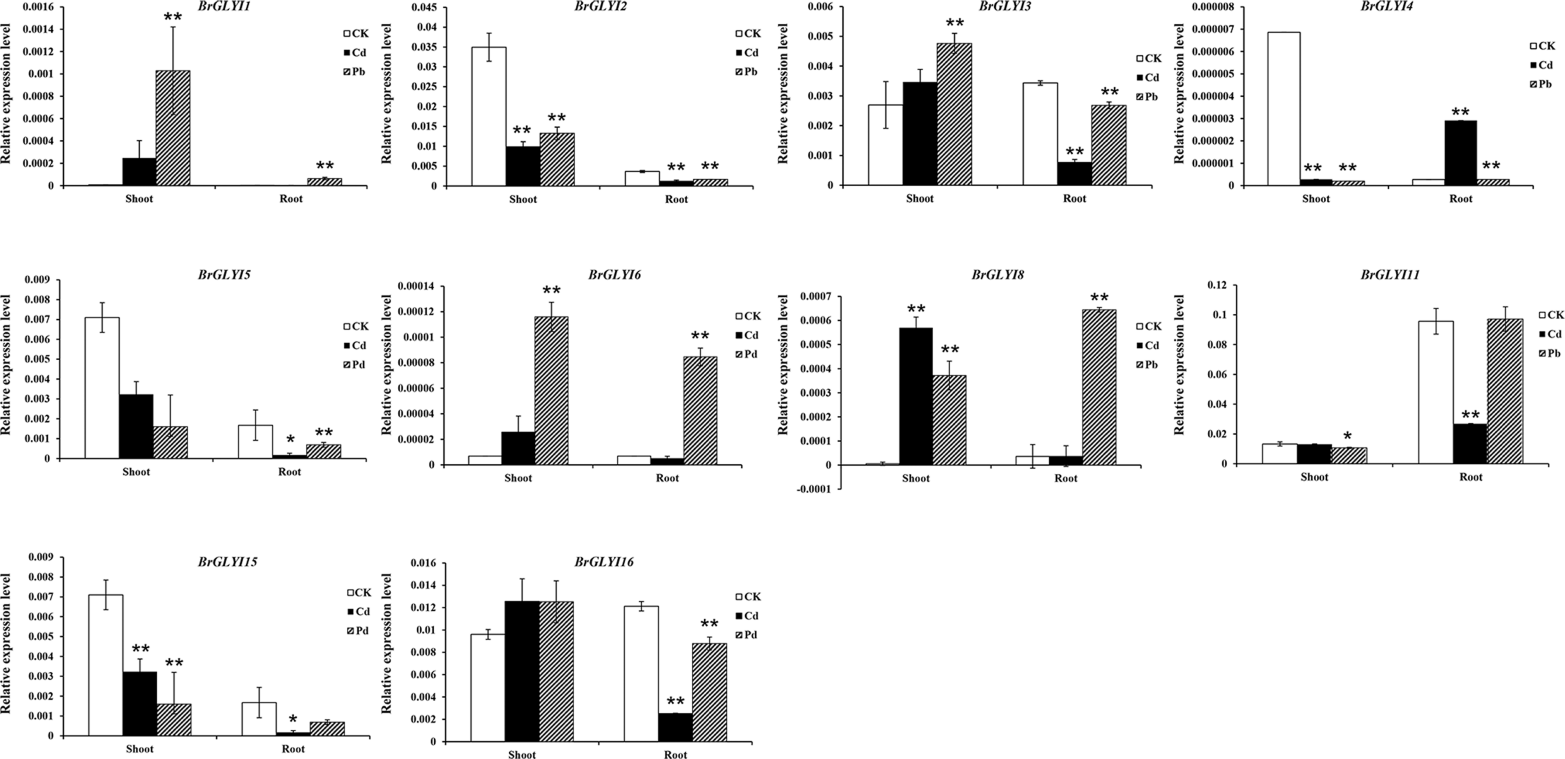
RT-qPCR assay of the expression of the representative *BrGLYI* under Cd and Pb treatments. The y-axis indicates the relative gene expression; the x-axis indicates the different treatments under the control (CK), Cd and Pb conditions.

To verify the response of the glyoxalase genes to heavy metals, RT-qPCR was performed to validate the nine candidate *BrGLYI* genes (*BrGLYI-1*, *2*, *3*, *4*, *5*, *6*, *8*, *11*, *15* and *16*) under the Pb and Cd treatment conditions (**Fig 8**). In the shoot, *BrGLYI8* were significantly up-regulated under the Cd condition and were approximately 1.8-fold higher than the expression under the control condition. The expression of *BrGLYI11* had no significant change under the Cd stress condition. Moreover, the expression of *BrGLYI3* and *BrGLYI6* showed significant increase under the Pb treatment compared with that in the control. Most interestingly, although the expression of *BrGLYI1* was almost undetected under the control conditions, its expression level was significantly induced under the Pb treatment conditions. In addition, the expression of *BrGLYI16* showed no significant difference under Cd and Pb treatments compared with control. In the root, *BrGLYI1*, *BrGLYI6* and *BrGLYI8* showed a significant up-regulation in response to Pb stress, and the expression level of *BrGLYI11* did not show any change under the Pb stress condition. However, the other *BrGLYI* genes were clearly suppressed under the stress conditions (**Fig 8**). These results indicated that *BrGLYI1*, *BrGLYI6*, *BrGLYI8*, *BrGLYI11* and *BrGLYI16* may play an important role in heavy metal resistance.

### Analysis of the regulatory elements in the *BrGLY* promoter

**Fig 9:**
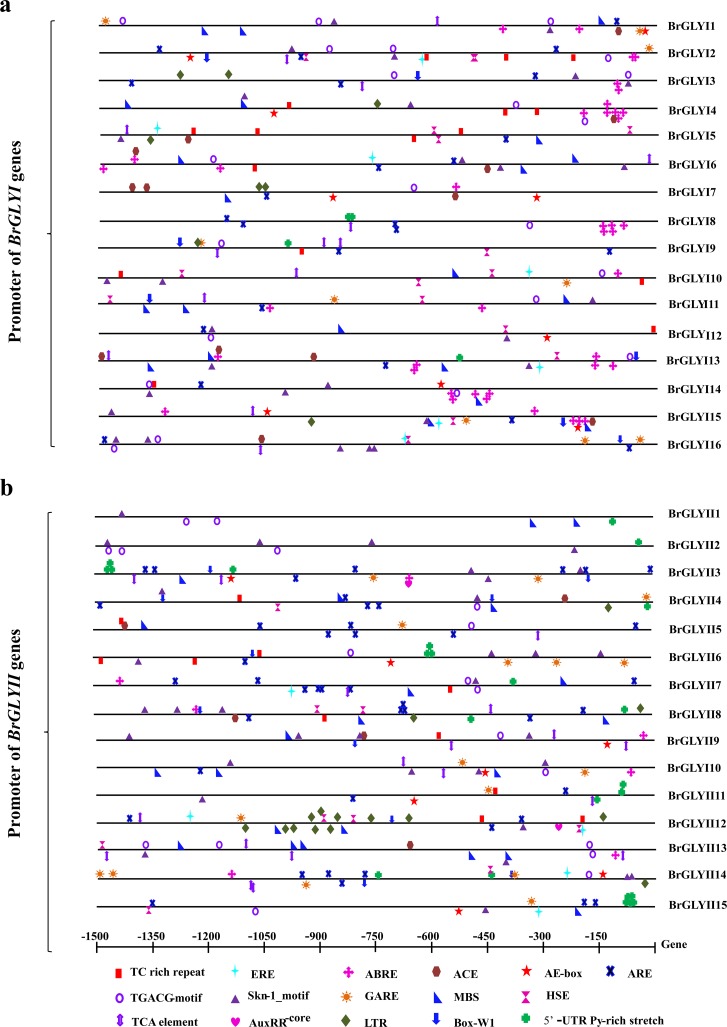
The promoter analysis in the *BrGLY* genes. Different elements are expressed by different color symbols and placed on the promoter according to their relative position. Symbols that are presented above the line indicate the elements at the forward strand, while those below indicate the reverse strand. The ABA-responsive element (ABRE), light response *cis-*acting element (ACE), light response module (AE-box), auxin responsive element (AuxRR-core), anaerobic induction element (ARE), fungal elicitor responsive element (BOX-W1), ethylene responsive element (ERE), gibberellin-responsive element (GARE), heat shock element (HSE), jasmonate and elicitor responsive element (JERE), low temperature responsive element (LTR), MYB-binding site (MBS), endosperm expression required element (Skn-1_motif), defense and stress responsive element (TC-rich repeat), salicylic acid responsive element (TCA), Methyl jasmonate-responsive element (TGACG motif), element conferring high transcription level (5’ UTR Py-rich stretch), and wounding and pathogen responsive elements (WUN-motif) were analyzed.

The *cis*-acting elements in promoter regions are known as regulation of gene transcription and their response to stress. Therefore, an analysis of 19 stress-responsive *cis*-acting elements in each *BrGLY* gene promoter was performed using PlantCARE database [40, 43], including ABRE, ACE, AE-box, AuxRR-core etc. (**Fig 9**). All these elements played a critical role in regulating gene transcription induced by various biological processes, such as biotic and abiotic stress responses, developmental processes, etc. Thus, the preliminary analyses of these elements will be helpful for understanding the gene^,^ responses to different stresses [49, 50]. These elements are distributed randomly in the *BrGLY* promoter sequences (including both positive and negative strands) without following a particular rule (**Fig 9**). Among the *BrGLYI* genes, the *BrGLYI12* promoter only has eight elements, while *BrGLYI2* and *BrGLYI13* have the maximum number of *cis*-elements (21 elements). Among the *BrGLYII* members, *BrGLYII12* has a maximum of 28 elements, while *BrGLYII2* has only eight *cis* elements. Almost every promoter region in the *BrGLY* genes contained ARE, Skn-1_motif and TGACG motif. Although the relationship between these elements and the responses of the genes under stress conditions requires further experimental investigation, our analysis results suggested that the *BrGLY* genes had a certain stress-responsive characteristic.

## Discussion

The genus *Brassica* is one of the most significant genera and is grown because its seeds, oil and vegetables have high nutritional value and include nutrients such as iron, vitamins, phytosterols and fiber [51]. The genus *Brassica* comprises six crop species. Among them, *B*. *rapa* (AA), *B*. *nigra* (BB), and *B*. *oleracea* (CC) were the ancestors of the three amphidiploid species *B*. *napus* (AC), *B*. *juncea* (AB) and *B*. *carinata* (BC) [52]. Chinese cabbage (*B*. *rapa* subsp. pekinensis), which is a type of *B*. *rapa*, is one of the most important vegetable crops in the world. The ‘A’ genome of *B*. *rapa* is valuable for gaining a better understanding of the genetic evolution of *Brassica* and expediting the genetic improvement of *Brassica* crops. Recently, many genomes of crop species, including *B*. *rapa*, have been sequenced, and the data have been released. Furthermore, bioinformatics analyses have developed rapidly. Therefore, we have the ability to identify large gene families in these species systematically.

MG is a cytotoxic metabolite generated from carbohydrate and lipid metabolism [1]. Previous reports have indicated that the level of MG increases when plants encounter various abiotic stresses [53]. The glyoxalase system, which contains GLYI and GLYII, can detoxify MG into D-lactate. The glyoxalase activity can be up-regulated under stress conditions, which reduces MG accumulation and protects plants from MG damage to a certain extent [10, 16, 26, 53–55]. Therefore, the two genes are suggested to be important candidate genes for improving plant tolerance by gene engineering. Recently, a genome-wide identification of the *GLY* gene has been performed preliminary in Arabidopsis and rice [34]. The analysis was also completed in soybean (*Glycine max*) and the results illustrate their developmental and stress specific responses [40]. However, the two gene families have not been analyzed in any other plant, including *Brassica* plants. In our study, *GLY* gene families in Chinese cabbage were identified at the genome level. The chromosomal location, gene structure, protein localization, protein motifs and expression patterns were then analyzed. In this study, we found 16 *BrGLYI* and 15 *BrGLYII* genes in Chinese cabbage. In a previous report, Arabidopsis and rice were shown to contain 11 *GLYI* genes and five and three *GLYII* genes [34], respectively. The number of *GLY* genes was lager in *B*. *rapa* than that in *Arabidopsis*, particularly the *GLYII* gene. Moreover, *AtGLYI10* did not have homologous genes in the three *B*. *rapa* subgenomes, and one *BrGLYI* and nine *BrGLYII* genes did not show homology to the *AtGLYI* genes. Therefore, the processes of polyploid evolution are likely accompanied by gene mutations and losses in addition to duplications or triplications.

The glyoxalase system is located in cellular organelles and cytoplasm. The widespread distribution of the GLY protein in living organisms indicates that it fulfills a function that is important to biological life. Previous studies suggested that the glyoxalase enzymes play a crucial role in tissue proliferation, cell division and malignancy [56–58]. In higher plants, GLYI activity was reported to be related to the cell division in pea, a *Datura* callus suspension and Brassica [16, 17]. Subsequently, the effects of GLYI on cell division and hormone levels were confirmed in soybean cell-suspension cultures [59]. In our study, we found that the expression of the five *BrGLYI* genes (*BrGLYI1*, *BrGLYI7*, *BrGLYI10*, *BrGLYI14* and *BrGLYI15*) in callus was much higher than that in the other tissues (FPKM > 200); however, *BrGLYII* did not show a similar expression pattern in callus. These results indicated that *BrGLYI* may play an important regulatory role in cell division as previously reported; however, its precise regulatory mechanism in cell division remains unclear and requires further study.

To investigate the response of the glyoxalase genes to various abiotic stress factors at the transcription level, the expression patterns of the *BrGLYI* and *BrGLYII* genes were analyzed using publicly available expression data and RT-qPCR. The expression of *BrGLYI4* was undetected. It may be that *BrGLYI4* had no expression or had spatial and temporal expression patterns. *BrGLYI8* and *BrGLYI6* expressed faintly; however, they were up-regulated under the Pb and Cd treatments. The two genes were selected to further study their functions. Several genes showed a high expression in specific tissues, such as the expression of *BrGLYI6* in siliques and that of *BrGLYI9* in silique walls. The abundant transcription of a gene in a specific organ usually suggests that the gene may play an important role in the development of the corresponding tissue. Many genes were highly expressed in more than one tissue and some genes were constitutively expressed in all the seven tissues, such as *BrGLYII12* and *BrGLYII14*. These genes may be required for development throughout the whole life. Moreover, the expression patterns of several *BrGLY* genes were inconsistent between the RT-qPCR and GEO data, such as the pattern for *BrGLYII2*. The possible reasons may be as follows: first, the plant materials were not sampled at precisely the same time, and some genes showed spatial and temporal expression patterns, and, second, the GEO data may not be specific to a gene because highly homologous genes might be difficult to distinguish.

The expression analysis of the *GLY* genes under the biotic and abiotic stress conditions showed that several *GLY* genes were stress responsive. *BrGLYI1*, *BrGLYI2*, *BrGLYI6*, *BrGLYI11* and *BrGLYI16* were up-regulated by more than 1.5-fold at different times when infected by *P*. *brassicae* in both the clubroot-resistant and clubroot-susceptible lines. Moreover, *BrGLYII8* and *BrGLYII10* were expressed at a high level after *P*. *brassicae* infection in both lines. Previous studies also showed that the *GLYI* genes were induced by pathogenic microorganism [9, 60, 61]. Thus, glyoxalases may play a crucial role in defending plants against infection by pathogens [21, 62, 63], and their function in plant disease resistance requires further investigation. In addition, *BrGLYI1* was significantly up-regulated under the ZnD condition, *BrGLYI13* was induced under the ZnE condition, and *BrGLYI15* was significantly induced under the FeD condition. Moreover, *BrGLYII5* was the most stress-inducible gene and was induced under the FeD, ZnD and ZnE stress conditions. The RT-qPCR analysis indicated that *BrGLYI1*, *BrGLYI3*, *BrGLYI6* and *BrGLYI8* were up-regulated under the Cd and Pb treatment conditions. In summary, using different Chinese cabbage varieties, we found that *BrGLYI6* and *BrGLYI1* may play an important role in tolerance to clubroot disease and heavy metal stress. The results will facilitate further functional exploration of these candidate genes in stress tolerance.

Moreover, many studies have confirmed that the glyoxalase pathway plays an important role in stress tolerance. In plants, previous reports have shown that transgenic plants overexpressing the *GLYI* genes have an improved tolerance to stress. Transgenic tobacco and *V*. *mungo* overexpressing *GLYI* from *B*. *juncea* had a high salt tolerance [3, 23]. Tobacco transgenically overexpressing *GLYI* and *GLYII* showed an enhanced tolerance to salinity and MG stress compared to that in wild type plants. Furthermore, when GLYI from rice, wheat, and sugar beet was expressed in tobacco, the transgenic tobacco showed an increased tolerance to salinity, heavy metal and MG stress [7, 9, 28]. Recently, we found that BnGLYI-3 transgenic yeast cells enhanced their tolerance to extreme temperature stress [2]. Jain et al. have found that the overexpression of *AtGLYI2*, *AtGLYI3* and *AtGLYI6* in *Escherichia coli* provides multi-stress tolerance (including salinity, exogenous MG, oxidative, mannitol and heat stress) [64]. Thus, the glyoxalase pathway is directly related to stress resistance in plant. In our study, certain *BrGLY* genes shared a high similarity with previously reported genes, and we speculated that these genes may have a similar function in Arabidopsis; for example, *BrGLYI5* shared approximately 87% identity with *ATGLYI2* and *BnGLYI-3*. Similarly, *BrGLYI11* and *BrGLYI9* showed 93% and 87% identity with *ATGLYI3* and *ATGLYI6*. In addition, further investigations should explore the mechanism of the response of the glyoxalase pathway to stress tolerance in plants to generate more stress-tolerant varieties using molecular approaches.

## Conclusion

We conducted a comprehensive analysis of glyoxalase gene families (*BrGLYI* and *BrGLYII*) in Chinese cabbage and then characterized 16 *BrGLYI* and 15 *BrGLYII* genes based on a genome wide sequence analysis. Detailed information, including chromosomal distribution, gene structure, duplication, phylogenetic relationships, conserved motifs, promoter *cis*-elements and the expression profiling in different organs and under biotic and abiotic stress conditions, was predicted and analyzed. Based on the phylogenetic analysis, the presence of conserved motifs and their corresponding expression, we provided insight into the possible function of these gene families in plant development and responses to specific stresses (pathogen infection and heavy metal stress). Our data shed light on the selection of candidate genes for stress tolerance and lay the foundation for further functional investigation on the *Glyoxalase* genes.

## Supporting information

S1 Fig **Conserved protein motif in (A) BrGLYI and (B) BrGLYII**.
(DOCX)

S2 Fig Logos of Chinese cabbage BrGLYI and BrGLYII protein motifs. The height of a letter indicates its relative frequency at the given position. (A) BrGLYI; (B) BrGLYII.
(DOCX)

S1 Table Specific primers used in the RT-qPCR analysis. (DOCX)

S2 Table The coding sequences of *BrGLY* genes in *B*. *rapa*. (DOCX)

S3 Table The amino acid sequences of *BrGLY* genes in *B*. *rapa*. (DOCX)

S4 Table Percentage of similarities among all BrGLYI proteins in Chinese cabbage. (DOCX)

S5 Table Pairwise similarities among paralogous pairs of BrGLYII proteins in *B*. *rapa*. (DOCX)

S6 Table Tissue-specific expression of BrGLYI and BrGLYII family genes ^a^. (DOCX)

## Abbreviations

aa: amino acid
ABRE: ABA-responsive element
ACE: light response *cis-*acting element
AE-box: light response module
ARE: anaerobic induction element
At: *Arabidopsis thaliana*
AuxRR-core: auxin responsive element
Bn: *Brassica napus*
*B. oleracea*: *Brassica oleracea*
BOX-W1: fungal elicitor responsive element
Bp: base pair
Br: *Brassica rapa*
BRAD: *Brassica* database
CdE: cadmium exposure
CDS: Coding DNA sequence
Ch: chloroplast
Cy: cytosol
ERE: ethylene responsive element
FeD: iron deficiency
FPKM: Fragments Per Kilo base of exon sper Million fragments mapped
GARE: gibberellin-responsive element
GLYI: glyoxalase I
GLYII: glyoxalase II
GSH: Glutathione (reduced)
Hai: Hours after inoculation
HMM: Hidden Markov Model
HSE: heat shock element
JERE: jasmonate and elicitor responsive element
Kb: Kilo base pair
LF: Least fractionated blocks
LTR: low temperature responsive element
MBS: MYB-binding site
MF1: Medium fractionated blocks
MF2: Most fractionated blocks
MG: Methylglyoxal
Mt: mitochondria
MW: molecular weight
NILs: Near-isogenic lines
NJ: Neighbor-joining
Nu: nucleus
ROS: Reactive oxygen species
S-LG: S-D-lactoylglutathione
Os: *Oryza sativa*
Pg: *Pennisetum glaucum*
PI: isoelectric point
PP: polypeptide length
RT-qPCR: quantitative real-time PCR analysis
ROS: Reactive oxygen species
Skn-1_motif: endosperm expression required element
kDa: kilodalton
TCA: salicylic acid responsive element
TC-rich repeat: defense and stress responsive element
TGACG motif: Methyl jasmonate-responsive element
5’ UTR Py-rich stretch: element conferring high transcription level
WUN-motif: wounding and pathogen responsive elements
ZnD: Zinc deficiency
ZnE: excess Zn
