# Supplementary figures and images for: Genome-wide analysis and expression profiles of glyoxalase gene families in Chinese cabbage (Brassica rapa L)
Source: PLoS One. 2018 Jan 11;13(1):e0191159. doi: 10.1371/journal.pone.0191159 (PMC5764358; doi:10.1371/journal.pone.0191159)

A


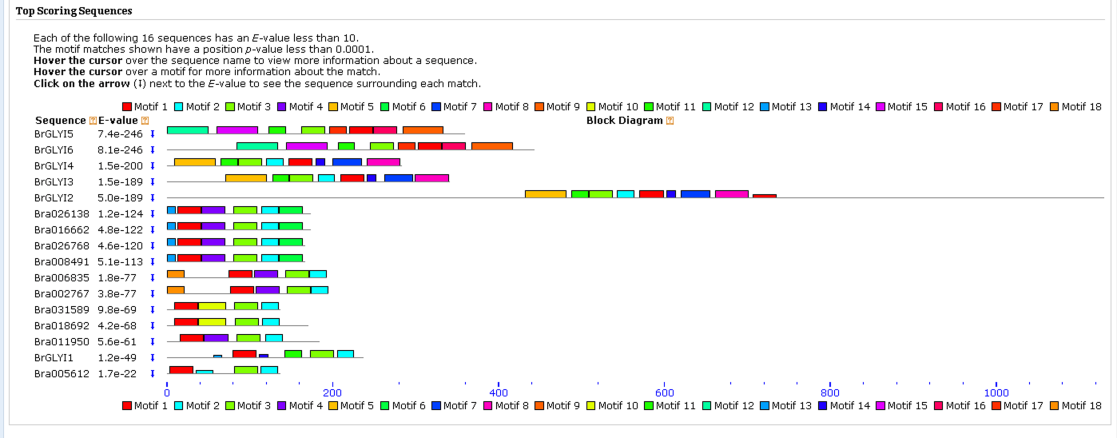


B


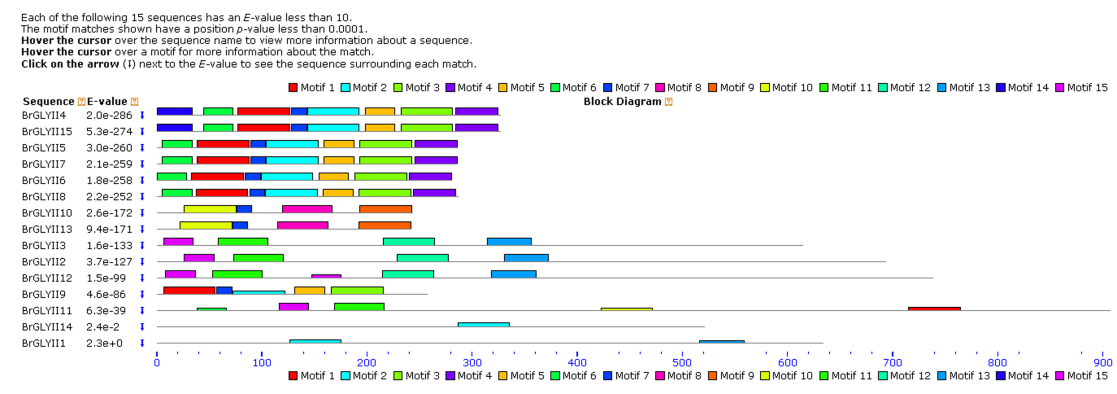


**S1 Fig. Conserved protein motif in** **(A)** **BrGLYI and (B) BrGLYII**

Supplement: S1 Fig — Conserved protein motif in (A) BrGLYI and (B) BrGLYII. (DOCX) [file pone.0191159.s001.docx]
