# Supplementary material for: Genome-wide analysis and expression profiles of glyoxalase gene families in Chinese cabbage (Brassica rapa L)
Source: PLoS One. 2018 Jan 11;13(1):e0191159. doi: 10.1371/journal.pone.0191159 (PMC5764358; doi:10.1371/journal.pone.0191159)
Supplement: S2 Fig — The height of a letter indicates its relative frequency at the given position. (A) BrGLYI; (B) BrGLYII. (DOCX) [file pone.0191159.s002.docx]

**A**


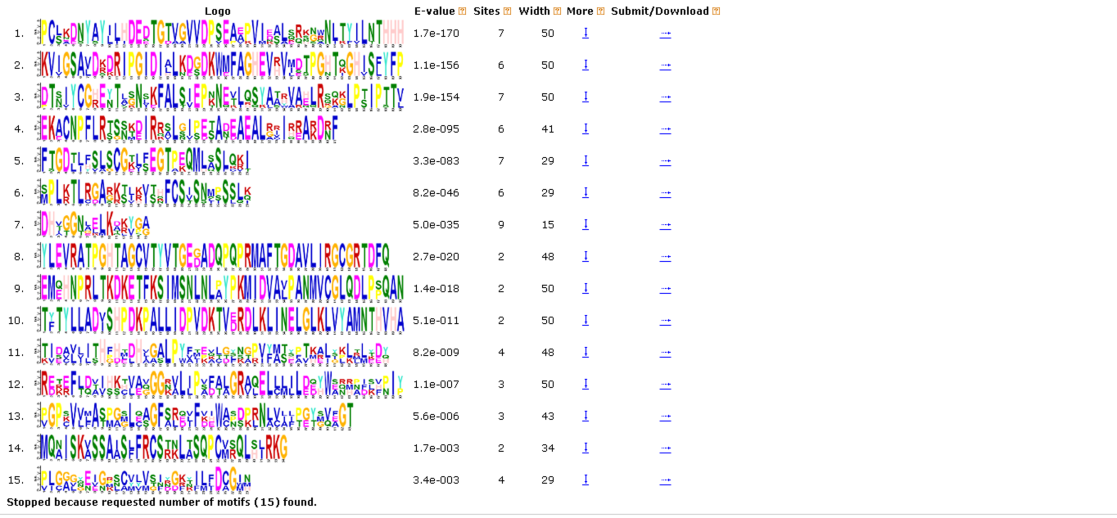


**B**

**
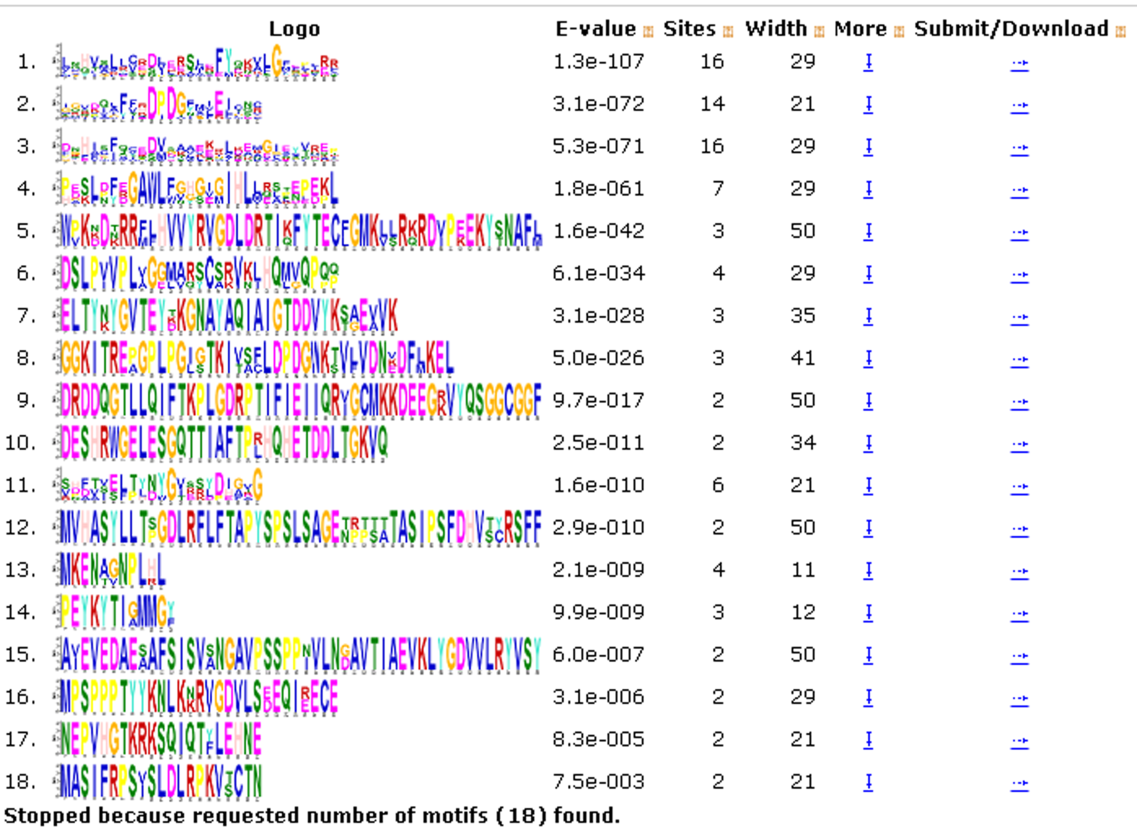
**

**S2 Fig. Logos of Chinese cabbage BrGLYI and BrGLYII protein motifs. The height of a letter indicates its relative frequency at the given position. (A) BrGLYI; (B) BrGLYII**
