# Supplementary material for: Genome-wide analysis and expression profiles of glyoxalase gene families in Chinese cabbage (Brassica rapa L)
Source: PLoS One. 2018 Jan 11;13(1):e0191159. doi: 10.1371/journal.pone.0191159 (PMC5764358; doi:10.1371/journal.pone.0191159)
Supplement: S1 Table — (DOCX) [file pone.0191159.s003.docx]

**S1 Table. Specific primers used in the RT-qPCR analysis**

| **GENE** | **FORWARD PRIMER 5’ - 3’** | **Tm**  **(**°C**)** | **REVERSE PRIMER 5’ - 3’** | **Tm**  **(**°C**)** | **FRAGMENT**  **(bp)** |
| --- | --- | --- | --- | --- | --- |
| BrGLYΙ1 | TGCAATCTACAGAGCCTGAG | 53.5 | CATACTCTATTCCCATTTCCTT | 52.8 | 129 |
| BrGLYΙ2 | GACCTGCACGAATCTCTCCA | 61.4 | GTGCTTGATTAGCTTTAACTCCAC | 58.6 | 107 |
| BrGLYΙ3 | GAGGTATTTGGGTTTGAGGA | 54.1 | GGAGGGTGGTTGAAGTGTTT | 56.2 | 121 |
| BrGLYΙ4 | GGTTGGATACGTTCGAGACA | 55.6 | ACTGGAAGATTAAGATTGATAGCT | 54.0 | 117 |
| BrGLYΙ5 | GCGAGAGATTTGAACAACTGGG | 63.2 | TCAAGCTGCGTTTCCGGCT | 66.5 | 146 |
| BrGLYΙ6 | GCTCAATCCGACAAGAAACC | 57.1 | TTGGCATACTCCAACCAGAAAA | 60.0 | 133 |
| BrGLYΙ8 | TGTTAGGGTTCGTGGAGACA | 56.0 | ATGGTCCGTGTTGGAAGGTA | 57.5 | 131 |
| BrGLYΙ9 | GAGGATCTTCTCAAGTGGGTC | 57.8 | CGACAGCAAT ACCAAAGTGA CC | 61.8 | 249 |
| BrGLYΙ11 | TGATGGGATATGCTGAGGAAT | 60.8 | CCTCCTAGCTCTTGGTTGGCT | 62.2 | 201 |
| BrGLYΙ15 | CCGGCGGAGAGAGATGGAGAGTAG | 60.5 | CTCTCCACCGGAAACATGGTTCACGC | 61.0 | 104 |
| BrGLYΙ16 | TCTTGCACGAATCATCTCCCTG | 64.7 | CAGCTGACCC GTGTGCTTGATT | 66.9 | 114 |
| BrGLYⅡ1 | CAGGCATTATTGTCTTGTCCGAC | 62.9 | TATATTCCCCTCAGGATGAAGT | 56.7 | 170 |
| BrGLYⅡ2 | AAAGGAGAGAGCAGCAGCCAAT | 63.9 | ACATGTAAAC GCAGGAACGA | 64.4 | 80 |
| BrGLYⅡ11 | TGCTCGCAGACCAGTTCCTACT | 63.6 | GAGGAGAAGTGACGGAACACGA | 63.9 | 133 |
| BrGLYⅡ12 | AGGTAGAGTTTGCAGGTGGAGG | 61.9 | ACACAACGGTCCTTCTATCAGA | 56.8 | 127 |
| BrGLYⅡ14 | CTCCCATCAGCAGATCTACCG | 62.1 | CGAAACTCCACTCTCCCACATC | 63.2 | 177 |
| *Actin* | CTGGAATTGCTGACCGTATGAG | 61.9 | ATCTGTTGGAAAGTGCTGAGGG | 62.8 | 145 |
