# Supplementary material for: Genome-wide analysis and expression profiles of glyoxalase gene families in Chinese cabbage (Brassica rapa L)
Source: PLoS One. 2018 Jan 11;13(1):e0191159. doi: 10.1371/journal.pone.0191159 (PMC5764358; doi:10.1371/journal.pone.0191159)
Supplement: S2 Table — (DOCX) [file pone.0191159.s004.docx]

**S2 Table. The coding sequences of *BrGLY* genes in *B. rapa***

| **Name** | **Locus**  **Identifier** | **Coding sequence** |
| --- | --- | --- |
| ***BrGLYI*** | | |
| *BrGLYI1* | Bra008491 | ATGAAAGAAAATACGGGAAACCCTCTCCATCTCAAGTCGTTGAACCACATATCTCTTCTA  TGTCGATCCGTGGAGGAGTCCATGAGCTTTTACCAGCACGTTTTAGGTTTTCTCCCGATC  AGGAGGCCTGGCTCTTTTGATTTCGATGGCGCCTGGTTGTTTGGTCATGGAGTTGGGATT  CATCTTCTGCAATCTACAGAGCCTGAGAAATTACTCAAGAAAACTGAGATCAATCCCAAG  GATAATCACATTTCTTTCCAGTGTGAGAGCATGGGGGCCGTAGAGAAAAAGCTTAAGGAA  ATGGGAATAGAGTATGTGAGAGCAGTGGTGGAAGAAGGTGGCATCCAAGTCGACCAGCTC  TTCTTCCACGATCCCGATGGCTTCATGATTGAGATTTGCAACTGCGACAGCCTTCCCGTC  GTGCCTCTTGCAGGAGAGATGGCTCGTTCATGCTCTCGTGTCAACATCCACCAACTGGTC  CAGCCACCTCAGATCCACCCCTAG |
| *BrGLYI2* | Bra006835 | ATGGCGTCTATTTTCAGACCTTCAATTTCACTAGACCTACGACCAAAGGTGACCTGCACG  AATCTCTCCACAAAAGAACGACTTGACTTTCACAACAAGAGTCTGAGAAAAGAGAAGCTC  AACATTAGGTTACGTGGAGTTAAAGCTAATCAAGCACAAGGGACTAGTGTGGTTACAGAA  GAGAAAGAGCTTAACAACAAAACCGATTATGGAGTTGTAGGGGTTCACCATGTGGGTCTG  CTGTGTGAAAACCTAGAACGGTCACTGGAGTTTTACCAGAACATTCTAGGCCTTGAGATC  AACGAGGCGAGGCCACACGATAAGCTTCCATATAGAGGAGCATGGTTATGGGTAGGTTCA  GAGATGATTCATCTAATGGAGCTTCCAAATCCTGATCCATTAACTGGTAGACCCGAGCAC  GGTGGTCGAGATAGACATGCTTGTATCGCAATCCGAGATGTTTCAGTTCTGAAAGAGATT  CTGGATAAAGCTGGGATTGCGTATACGATGAGTAAGTCAGGGAGGCCAGCGATATTCACT  CGTGATCCAGACACAAACGCTCTTGAGTTTACTCAAGTTTGA |
| *BrGLYI3* | Bra005612 | ATGGCGACTCTAGGCCATATCGCGAGAGAATCATCCGACGTCACACGCCTCGCCCTGTTC  TACAAAGAGGTATTTGGGTTTGAGGAGATAGAGAGCCCTGATTTTGGAGACCTCAAGGTG  ATATGGCTAAACTTACCAGGTGCTTTTGCGATGCACATCATCCAGAGAAACACTTCAACC  ACCCTCCCTGAAGGTCCTTACAGCGCCACCTCTGCCGTTCGAGATCCTAGCCATCTCCCT  ATGGGCCACCACATCTGCTTCTCCGTCTCCAACTTCGACTCCTTCCTTCGCTCTCTCAAG  GAGAAAGGGATAGAAACTTTCCAGAAGTCTCTTCCTGATGGAAAAGTCAAGCAAGTTTTC  TTCTTTGATCCTGATGGAAACGGATTAGAGGTAGCAAGTCGATCTGAGTCTTGA |
| *BrGLYI4* | Bra018692 | ATGGCGTCGAATGTCATAAAACCAGCGTATGCTTACACGGTTGTGTACGTTAGGGACGTC  GCGAAATCCGTAGAATTCTATTCGAGAGCCTTTGGTTACAACGTTCGTCGTCTTGACGAG  TCCCACAGGTGGGGGGAGCTAGAGAGCGGGCAGACAACGATAGCATTCACACCGCTTCAC  CAGCATGAGACGGATGACCTAACCGGTAAAGTTCAGTCGTCTACGCGTTCAGAGCGTGAG  AGAGCACCCCTCGAAGTCTGCTTCTGTTATGCTGATGTTGACGCGGCTTTCAAGAGGGCT  GTGGAGAATGGTGCGGTGGCTGTGAGCGAGCCGGAGGACAAGGAATGGGGCCAAAAGGTT  GGATACGTTCGAGACATTGATGGCATCGTTGTACGCATCGGAAGCCACGTTAAGTCATAT  AAACCTTGGACAAGTTTCGGGTTTTCTCTAGCTATCAATCTTAATCTTCCAGTTGTTCCA  CAGCTCAACAATATAACAAGCAAAAAAAGGCAATAA |
| *BrGLYI5* | Bra018654 | ATGGGTTCATACTCAATAGCAAGTGCTATATCTCGAGTTTCAGTTTCGCCTCTAAACCGT  TTCGTTAAACTTTATTCCACCGCTTTTTCTTACATCAATTGCCCTTGCAACAGAGCCCTG  AGGCCAAAGAGATTTGATCAGCTTCGTGTGTTCTCTATGGCGTCGGAAGCGAAGGAATCA  GCAGCAAACAATCCTGGCTTGTCAACGGTTCGGGATGAGGCTACCAAAGGGTATATCATG  CAGCAGACTATGTTTCGGGTGAAGGACCCTAAGGCTAGTCTTGACTTTTACTCACGTGTC  CTGGGAATGTCATTGCTCAAGAGGCTAGATTTCTCTGAGATGAAGTTCAGCTTGTACTTC  CTGGGCTACGAGGATACTTCGACAGCTCCAACAGATCCTACTGAGAGAACTGTTTGGACC  TTTGGTCGACCTGCAACAATTGAGCTGACTCACAACTGGGGCACAGAGAGTGATCCTGAG  TTTAAAGGCTATCATAATGGAAACTCCGAGCCTCGTGGATTTGGGCATATTGGGGTTACA  GTTGATGATGTGCACAAGGCATGCGAGAGATTTGAACAACTGGGAGTAGAGTTCGTCAAG  AAACCGAATGATGGAAAGATGAAGAATATAGCCTTCATCAAGGATCCTGATGGCTACTGG  ATCGAGATCTTTGATCTCAAGACTATCGGGACAACAGCCGGAAACGCAGCTTGA |
| *BrGLYI6* | Bra019830 | ATGGCTCTGAGGTGTTTCCCCATCTGGGCTTGTCCCCAGACGGCTTATTATCATTACCCT  CTGCTTGGTTTGGACACGAAACGCCGTCGTATCCCCCTCTGGGAATGCTCCTCCTCGGCT  TCCCAGAGAGCCGTCACTGCCGTTGGAAGCGAGGTTCCGTACGGACGTGAGCTCAAGAAG  CCCTCTGATGAAATGGGTCTGACTCAGGAGAGTCCCCAGCTGGAAACTTTTCATAGAGAC  TTGAGTATGCTTCCTAAACCGCTAACTGCAAACAGTCTTACCTCTTCTGCTGGGGATGAT  TCAAAAGTGCGAATTTCGTTTCAGGGCATACCAGGTGCGTATAGCGAGACAGCAGCACTG  AAAGCATATCCAAACTGTGAAACCGTACCATGTGACCAGTTTGAAACTGCGTTCCAGGCT  GTGGAGCTTTGGTTGGTGGATAAAGCAGTATTACCGATAGAGAATTCAGTAGGTGGTAGT  ATCCACCGTAACTATGATTTGCTTCTTCGTCATAGGCTTCACATTGTCCAAGAAGTTCAT  TTGCCTATTAATCATTGTCTCTTGGGAGTCCCTGGTGTTAGTATAGAAGATATTAAATGC  GTTCTAAGCCATCCTCAGGCGCTTGATCAATGTGTGAACTCACTTAACGATTTAGGCATA  CAGAGAGTCTCTGCAAAAGACACTGCTACTGCTGCTCAGACTGTTTCTTCGAGCGGCGAG  AGGAGTATAGGAGCAGTGGCGAGTGTGCGTGCTGCAAATATATACGGTCTTGATATTCTT  GTAGAGAACATACAAGATGATGCTAACAATGTGACACGGTTTCTTATACTTGCGAGAGAT  CCTATGATTCCAAGAACAGATCGACCATATAAGACAAGTATTGTGTTCTCGCTAGAAGAA  GGTCCTGGTGTGCTGTTCAAGGCCTTGGCTGTTTTTTCTTTAAGAAACATTAACTTATCC  AAGATAGAAAGTCGTCCGCAGAGAAGAAGACCGCTAAGAGTAGTTGATGGTTCTAACAAT  GGATGCGCCAAGTACTTTGATTACTTATTCTACATTGATTTTGAAGCTTCCATGGCTGAA  ACACGTGCTCAGCACGCCCTTGGCCATTTACAGGAATTCACCAGTTTCATCCGTATCCTG  GGGTGCTATCCTATGGATCTAGTCAGCAGTAGCAGAGAAGTTGTCGGTCAGTTAGAAACT  GATTGTTTTTCAAGTGACGGACGTTCCAAGTCCAGTGGTATCTGCTCGCCTCGTGCATCT  TCTCTCAGAAAATTGGCTGATGTTGCTTCATTGGATTGGCCAAAGAATGACACGCGCCGT  TTCTTCCATGTTGTATACCGTGTTGGTGATCTTGACCGCACAATCAAGTTTTACACTGAG  TGCTTTGGCATGAAGGTGTCGAGGCAAAGAGATGTCCCTAAGGAGAAGTATTCTAATGCT  TTTATGGGTTTTGGATCTGAAAAATCCCACTTCGCTGTGGAGCTCACTTATAATTATGGT  GTTAGCTCATATGACATTGGAGATGGATTTGGTCATTTCACCATTTCAACTCAAGATGTT  TACAAAATGGTTGAGACCGTACGTGCCAAGGGTGGAAATGTCACCAGAGAACCTGGTCCG  GTCGAAGGTGGAAGCAGCATCATTGCCATTGTGAAGGACCCTGATGGTTACCCTTTTGAA  CTCATCCAGAGAGGTCCAACTCCTGAACCATTCTGTCAAGTCATGCTTCGTGTTGGTGAC  CTTGACCGCGCCATCAAATTCTATGAGAAGGCCCTTGGGATGAGACTCTTGAGAAGGATT  AAGAAACCTGAATACAAGTACACCATAGGCATGATGGGATTTAATGAGTCGGTAGTTTTG  GAGCTAACCTATAAATATGGCGTGACTGAGTACAAAAAGGGCAATGCATATGCACAGATT  GCAATAGGCACTGATGATGTGTACAAAAGCGGTGAAGTTGTGAAGATAGTCAATAAAGAG  CTAGGAGGAAAGATCACTAGAGAACCAGGACCACTTCCTGGAATAGGCACTAAGATTGTC  TCATTTCTCGATCCAGATGGCTGGAAAACGGTTCTGGTAGACAACAAAGACTTTATGAAG  GAACTAGGCGAATCATCAGTTAATTACGTAAGTAGTAGTGTCAAGAATCTGGAAGAAGCT  CCAGAGGCAAAAACAAAGTCACAATCTTTTGAGGAGGAGGAGGAGAAGAAGAAGATGAGA  GGTGGGAGTCTATGGCAGCTAGGCCAATCCATAACCCGGCGTCTTGCTCAATCCGACAAG  AAACCTCTGTCACGCCGCTACTTAGCCTCTGGTGCTGACCTGAAAAAGACTGCTCTTTAC  GACTTCCACGTCGCTCATGGAGGAAAGATGGTTCCCTTTTCTGGTTGGAGTATGCCAATA  CAGTACAAAGACTCCATCATTGACTCAACGGTTAACTGCAGGGTCAATGGGAGTTTGTTT  GATGTTGCACATATGTGTGGTTTGAGTCTTAAAGGCAAAGACTGTGTTCCCTTTCTGGAG  AAGCTCGTGGTTGCTGACGTGGCTGGTTTGGCTCCTGGGACAGGGAGTTTGACTGTGTTT  ACAAACGAGAAGGGAGGTGCAATTGATGACTCGGTGATTACTAAAGTGACTGATGAGCAT  ATCTATCTGGTGGTGAATGATGGTTGTAGAGATAAGGATTTGGCTCATATTGAGGAACAC  ATGAAGGCTTTTAAATCCAAAGGAGGTCCTTTGGCTGCTCCGCTGCTTCAACACTTGACT  AAAGAAGACTTGAGCAAGCTTTACTTTGGACAGTTCCAGATTCTGGACATTAATGGTTCC  ACCTGCTTCCTTACCAGGACCAGCTCAAGAGGAAGAGTTCAAGAAGATCTCACAGTTAAT  GAATCTCTCTGTTTTGGCTTTACAGCCAAAGCAATCTTGGAGAAGTCCGAGGGAAAGGTA  AGGCTAACGGGTCTAGGAGCAAGAGACAGTCTCAGGCTAGAAGCAGGGCTTTCTCTATAC  GGCAATGACATGGAGCAACACATCTCTCCTGTTGAAGCGGGCCTCACATGGGCCATAGGG  AAACGTAGAAGAGCAGAAGGCGGGTTTCTTGGCGCGGATGTGATCCTCAAACAGCTAGAA  GATGAACCTACGATCAGAAGGGTGAGGTTTTTCTCATCGGGACCACCAGCGAGGTCGCAT  AGCGAGGTCCATGATGAGAATGGTAACAAGATTGGAGAGATCACGAGTGGTGGGTTTAGT  CCTAACCTGAAGAAGAACATAGCTATGGGGTATGTGAAGTCTGGTCAGCACAAGACAGGG  ACTAAAGTCAAGATCTTGGTACGTGGGAAACCTTATGAAGGTAATATCACCAAGATGCCG  TTCGTGGCTAACAAATACTACAAGCCATCATGA |
| *BrGLYI7* | Bra026138 | ATGAAGGAGAACGCAGGAAACCCTCTCCATCTCACGTCACTGAACCATGTATCTCTCTTG  TGCCGATCCATTGAAGAATCCATGGTTTTTTACCAAACGGTGTTAGGGTTCTTCCCTATT  CGAAGACCTGAGTCTTTAAATTTTGAAGGCGCTTGGTTGTTTGGACATGGAATTGGAATA  CATCTCTTGCGTTCCTCAGAACCAGAGAAACTTCCCAAGAAAACCGAGATCAATCCCAAA  GACAACCATATCTCCTTCCAGTGCGAGAGTATGTCAGCAGTGGAGAAGAAGCTTGAGGAA  ATGGAGATAGAGTATGTGAGGGCGATAGTTGAAGAAGGAGGGATCCAAGTGGATCAGCTT  TTCTTCCACGATCCAGATGGCTTCATGATTGAGATATGTAACTGTGATAGTCTCCCCGTT  GTCCCCCTCATAGGAGGAATGGCTCGGTCCTGCTCCAGAGTTAAACTTCATCAGATGGTG  CAGCCACAACAGCAGACTCAGATCCACCAAGTGGTCCACCCTTAA |
| *BrGLYI8* | Bra011950 | ATGGAGCAAAAGAACAAAAGCGACGAATCAAGGCCGCCATTGATGGCGTTAAACCATGTA  TCAAGACTTTGCAGAGACGTCAAAAAGTCTCTCGAGTTCTACACGAAAGTGTTAGGGTTC  GTGGAGACAGAGCGACCCGCGTCGCTAGACTTCGACGGTGCGTGGCTATTCAACTACGGT  GTTGGGATCCATTTGGTGCAGGCTAAAGACGAAGAGAAGCTACCTTCCAACACGGACCAT  TTGGACCCGATGGATAACCACATCTCGTTCCAGTGCGAAGACATGGAAGCTTTGGAGAAG  AGGCTCAAGGAAGTGGATGTGAAGTACATCAAGAGGACGGTCGGTGAGCAGGAAGACGCA  GCTATCGACCAGCTCTTCTTTAATGACCCCGATGGTTTCATGGTCGAGATTTGTAACTGC  GAGAATCTGGAGCTTAAACCGCGTGATTCAGCTGATGCTATACGTCTCCCGGGTGATCGA  CACGCGCCTCCTGTTTCTCTCCCTGGCTCGTCCGACCATGCAGATGATACTAGGCTCCCT  CAGACCAATTCTTAA |
| *BrGLYI9* | Bra004214 | ATGAGGATAATCTCAACGGCCTCCACCATCCGACCTTCCTTGCTAGGCTGCGTCTCTGCA  TCATCTCCTCGCTTCCCCGTCGTCTCCAGGAATCTCTCTTTCTCCCATGTTACTCAGTCA  AAGCTTTTGACTTTGAGAAGAAGCGTGAGCTGCTTAGGAGTAGCTGAATCTGGAAAAGCA  AGCACGGCCGCTACTGAAGAGGATCTTCTCAAGTGGGTCAAAGATGACAACAGAAGGATG  CTTCACGTCGTTTACCGTGTTGGCGATTTGGACCGGACCATAAAATTCTATACGGAATGT  CTTGGAATGAAGCTTCTACGCAAGCGTGATATACCGGAGGAGAAGTATACAAATGCTTTT  CTTGGTTATGGTCCTGAAGATTCGCACTTTGTCATTGAGCTCACTTATAATTATGGAGTT  GACAAGTATGACATTGGGGCAGGTTTTGGTCACTTTGGTATTGCTGTCGACGATGTGGCG  AAAACTGTAGAACTTATAAAAGCCAAAGGGGGCAAAGTAACAAGGGAGCCTGGTGCTGTC  AAAGGTGGCAAAACTGTAATCGCATTCATTGAAGATCCTGATGGTTACAAATTTGAACTC  TTGGAGAGAGGTCCTACACCTGAACCTCTTTGCCAAGTTATGCTCCGTGTTGGTGATCTC  GACAGGTCCATCAAATTCTACGAGAAGGCTTTTGGAATGGAACTTCTGCGCACAAGAGAC  AATCCAGAGTACAAGTACACAATAGCTATGATGGGATATGGTCCAGAAGACAAAACTGCA  GTCCTAGAGCTGACATATAACTATGGTGTCACTGAATATGATAAAGGAAATGCTTATGCT  CAGATTGCAATAGGAACGGATGACGTGTACAAAACCGCAGAGGCTGTTAAACTCTTTGGT  GGGAAAATCACGAGGGAACCTGGTCCCTTACCAGGTATAAGCACGAAGATCACCGCATGT  TTGGATCCAGATGGTTGGAAGTCGGTGTTTGTGGACAACGTTGATTTTCTCAAAGAACTG  GAGTGA |
| *BrGLYI10* | Bra016662 | ATGAAGGAAAACGCAGGAAACCCTCTCCATCTCACGTCACTGAACCATGTGTCTCTCTTG  TGCCGATCCATCGAAGAATCTATGAACTTTTACCAAAAGGTGTTAGGGTTCTTCCCTATT  CGAAGACCTGAATCTTTAAATTTTGAAGGCGCTTGGTTATTTGGACATGGAATTGGAATA  CACCTCTTGCGTGCCCTAGAACTTGAGAAACTTCCAAAGAAAAATGAAATTAACCCCAAA  GATAATCATATCTCTTTCCAGTGCGAAAGTATGGGAGCAGTGGAGAAGAAGCTTGATGAA  ATGGAGATAGACTATGTGAGGTCTAAAGTGGAAGAAGGAGGGATCCAAGTGGACCAGCTC  TTCTTCCACGACCCTGATGGTTTCATGATCGAGATCTGCAACTGTGACAGCCTCCCCATT  GTCCCCCTCGTAGGAGGCATGGTTCGGTCTTGCTCAAGAGTCAAACTCCATCAGATGGTG  CAGCCACAACCCCAAACTCAGATCAACCAAGTGGTCCATCCGTAA |
| *BrGLYI11* | Bra016811 | ATGGCAGAAAATGCTGATTTGTTGGAGTGGCCAAAGAAGGATAAGCGTCGTTTTCTCCAT  GTTGTGTACCGCGTTGGTGATCTTGATCGCACTATCCAGTTCTACACTGAGTGCTTTGGC  ATGAAGCTGTTGAGGAAAAGAGATGTCCCTGAGGAGAAGTACTCTAATGCTTTCCTTGGT  TTTGGTCCTGAAACCTCCAACTTCGTTGTCGAGCTCACTTACAATTATGGTGTAAGCTCA  TATGACATTGGAACTGGATTTGGGCATTTCGCTATTTCAACTCAAGATGTTTCCAAGATG  GTTGAGGCGGTTCGTGCCAAGGGTGGAAATGGCACCAGAGAGCCTGGTCCGGTCAAAGGT  GGAGGCAGTGTTATTGCGTTTGTAAAGGACCCTGATGGTTACATGTTTGAGCTCATCCAG  AGAGGTCCAACTCCTGAACCTCTCTGTCAAGTCATGCTTCGTGTTGGTGATCTTGACCGT  GCCATCAAGTTCTATGAAAAGGCCCTAGGGATGAGACTCTTGAGAAGGATTGAGAGACCT  GAATACAAGTACACCATAGGCATGATGGGATATGCTGAGGAATATGAGTCCATAGTTTTG  GAGCTGACCTATAACTACGGCGTGACTGAGTACACAAAGGGCAACGCATATGCACAGATT  GCAATAGGCACGGATGATGTGTACAAAAGCGCTGAAGTAGTGAAGATAGCCAACCAAGAG  CTAGGAGGAAAAATCACAAGAGAAGCCGGACCTCTTCCTGGACTCGGCACCAAGATTGTC  TCCTTCCTCGATCCAGATGGGTGGAAAACAGTCCTGGTAGACAATGAAGATTTTCTGAAG  GAACTGGAATGA |
| *BrGLYI12* | Bra026768 | ATGAAGGAAAACGCAGTCAACCCTCTCCGTCTCACGTCACTGAACCATGTGTCTCTCTTG  TGCCGATCGCTCGAAGAGTCGATGAATTTTTACCAAAAGGTCTTAGGGTTCTTCCCAGTT  CGAAGACCTGAGTCTTTAGATTTTGAAGGCGCTTGGTTGTTCGGACATGGAATTGGAATA  CATCTCTTGCGTTCCACAGAGCCTGAAAAACTTCCCAAGAAAACTGCGATTAATTCCAAA  GATAACCACATCTCTTTTCAGTGCGAGAGTATGGCAGCAGTGGAGAAGAAGCTTGATGAA  ATGGAAATAGAGTATGTGAGGGAGATAGTAGAAGGAAGAGGGATCAAAGTGGACCAGATC  TTCTTTCACGACCCTGATGGCTTCATGATAGAGATCTGCAATTGCGATAGCCTCCCCGTT  GTCCCCCTCGTAGGAGGACTGGCCCAGTACTGCGCAAAAGTTAAACTTCATCAGATGGGG  CAGCCACAACCGCAGACTAATTAA |
| *BrGLYI13* | Bra031589 | ATGGCGTCGAATATCATGAGACCAGCGTTTGCTTATACGGTTGTGTACGTGAAGGACGTG  GCCAAATCCGTAGAATTTTACTCTAGAGCTTTTGGTCACAACGTCCGTCGTCTTGACGAG  TCCCACAGGTGGGGAGAGCTAGAGAGCGGACAAACGACGATAGCCTTCACACCGCGTCAC  CAGCATGAGACCGACGACTTAACGGGGAAGGTACAGGCTACGCATTCAGACCCTGAGAGA  GCACCCATCGAAGTCTGCTTCTGTTACCCGGATGTTGATGCCGCCTTCAAGAGGGCTGTG  GAGAACGGTGCGGTGGCTGTCAGCGAGCCAGAGGACAAGGAATGGGGACAGAAGGTTGGT  TACGTGCGAGACATTGACGGCATCGTTGTCCGCATTGGTAGCCACGTTAAATGA |
| *BrGLYI14* | Bra032415 | ATGGTTCACGCCTCCTACCTACTCACCCCCGGCGACCTCCGATTCCTCTTCACCGCTCCC  TACTCTCCATCTCTCTCCGCCGGCGAAACTCGAACATCCGCCACAGCCTCAATCCCATCC  TTCGATCACGTCTCTTGCCGCTCCTTCTTCTCTTCGCACGGACTCGGCGTAAGAGCAGTC  GCCATCGAAGTCGAAGACGCTGAGTCAGCATTCTCCATCAGCGTCGCAAACGGCGCCGTT  CCTTCCTCCCCTCCTAACGTCCTCAACGGAGCCGTTACGATCGCGGAGGTTAAACTATAC  GGAGACGTCGTCCTCCGTTACGTTAGTTATCATAACGGAGCCGTTAATTTCCTCCCCGGA  TTTGAATCTGTTGACGATACGTCGTCGTTTCCGCTAGATTACGGTATACGCCGTCTCGAC  CACGCAGTGGGGAACGTCCCCGAGCTGGGCCCAGCTTTAACTTACCTCGCGGGGTTCACA  GGCTTCCACCAGTTCGCGGAGTTCACGGCAGACGACGTGGGAACAGCCGAGAGCGGTTTA  AACTCGGCTGTTTTAGCCAGCAACGACGAGATGGTTCTGTTGCCGGTGAACGAGCCGGTG  CACGGGACGAAGAGGAAGAGTCAGATCCAGACGTTTCTTGAACACAACGAAGGAGCCGGG  CTGCAGCATTTGGCTCTGATGAGCGAAGATATATTCAGGACGCTGAGGGAGATGAGGAAG  AGGAGCGGCGTTGGAGGGTTTGACTTCATGCCTTCTCCTCCGCCTACTTATTACAAGAAT  CTCAAGAAAAGGGTTGGAGATGTGCTGAGTGATGAGCAGATTAGGGAGTGTGAGGAGCTG  GGGATTCTTGTGGATAGAGATGATCAGGGGACGTTGCTTCAGATCTTTACAAAACCACTT  GGTGACAGGCCGACGATATTTATAGAGATAATACAGAGAGTGGGATGCATGAAGAAGGAT  GAGGAAGGGAAGGTTTACCAGAGCGGAGGATGTGGTGGGTTTGGTAAAGGTAACTTCTCT  GAGCTTTTTAAGTCTATTGAAGAGTATGAGAAGACTCTTGAAGCCAAGCAGCTTGTGGGG  TGA |
| *BrGLYI15* | Bra015511 | ATGGGGCACGAAAACGCAGCCGTTTCAGAGAACCAGCATCACGACGACGCTGCAACTACC  TCCGCGTCCCCGGGGTTTAAGCTCGTCGGATTCTCCAAGTTCGTGAGGAAGAATCCAAAG  TCCGACAAGTTCAAAGTCAAGCGCTTCCACCACATCGAGTTCTGGTGCGGCGACGCCACC  AACGTCGCACGCCGCTTCTCGTGGGGACTCGGCATGAGATTCTCCGCCAAATCCGATCTC  TCCACCGGAAACATGGTTCACGCCTCCTACCTCCTCACCTCCGGCGACCTCCGATTCCTC  TTCACCGCTCCCTACTCTCCATCTCTCTCCGCCGGCGAGAATCCACCGACCACCACAGCC  TCCATCCCATCTTTCGACCACGTCACCTACCGCTCCTTCTTCTCCTCACACGGTCTCGGA  GTAAGAGCAGTCGCTGTTGAAGTAGAAGACGCAGAGGCAGCCTTCTCCATCAGTGTCTCA  AACGGCGCCGTTCCTTCATCCCCTCCTATCGTCCTAAACGACGCCGTTACGATCGCTGAG  GTTAAACTATACGGCGACGTCGTTCTCCGTTACGTTAGTTACAAAGTAGCAACCGTTTTC  CTTCCAAGATTCGAAACTGTGGATGACACGTCGTCGTTTCCACTAGACTACGGTATACGC  CGCCTCGACCACGCGGTTGGAAACGTCCCCGAGCTCGGTCCAGCGCTAACTTACCTCTCA  AGGCTCACCGGCTTCCACCAGTTCGCGGAGTTCACAGCGGACGACGTGGGAACAGCCGAG  AGCGGTTTGAACTCGGCGGTTCTGGCTAACAACGACGAGACGGTTCTTCTGCCGGTCAAC  GAGCCGGTTCACGGGACGAAGAGGAAGAGTCAGATCCAGACGTATCTGGAGCACAACGAA  GGCGCGGGGGTGCAGCATCTGGCGCTGATGAGCGAAGACATATTCAGGACTCTGAGGGAG  ATGAGGAAGAGGAGCGGCGTTGGAGGTTTTGACTTCATGCCTTCTCCTCCGCCTACTTAC  TACAAGAATCTCAAGAACCGTGTGGGAGATGTGCTTAGCGAGGAGCAGATTGAGGAGTGT  GAGGAGTTGGGGATTCTTGTGGATAGAGATGATCAAGGGACGTTGCTTCAGATCTTCACT  AAACCACTTGGTGACAGGCCGACGATATTTATAGAGATAATACAGAGGATAGGATGCATG  AAGAAAGATGAGGAAGGGAGAGTTTACCAGAGTGGAGGATGTGGTGGCTTTGGCAAAGGC  AACTTCTCTGAGCTTTTCAAGTCTATTGAAGAGTATGAGAAGACTCTTGAAGCTAAACAG  CTTGTGGGGTGA |
| *BrGLYI16* | Bra002767 | ATGGCGTCTATTTTCAGACCTTCAGTTTCACTTGATCTACGACCAAAGGTGTCTTGCACG  AATCATCTCCCTGCGATAGAACGATTCGAGTTTCAGAAGAACAAGAATCTGAGAAAAGAT  AGGCTCAATGGTATCTTGAAGGCTAATCAAGCACACGGGTCAGCTGAAGGGATCAGTGTG  GTACAAGAGAAAGAGATTAACAATCAAACCGATTATGGAGTTGTTGGGGTTCACCACGTT  GGTTTGCTCTGCGAGAACTTAGAACGGTCACTGGAGTTTTACCAGAACATTTTAGGACTT  GAGATCAACGAGGCGAGACCACATGATAAGCTTCCGTATAGAGGAGCATGGTTATGGGTA  GGCTCGGAGATGATTCATTTAATGGAGCTTCCAAATCCTGATCCGTTAACAGGCAGACCC  GAGCACGGAGGCCGGGATCGACACGCTTGTATCGCAATCCGTGATGTTTCATATCTTAAA  GAGATTTTGGACAAAGCTGGGATTGAGTATACTATGAGTAGGTCGGGGAGGCCAGCGATA  TTCACTCGTGATCCAGACGCAAACGCTCTTGAGTTTACTCAAGTTTGA |
| ***BrGLYⅡ*** | | |
| *BrGLYⅡ1* | Bra011454 | ATGTCTGCGGTTATTAAACAACCAACCTGTAAGGAAGAAAGGATGAACGCTCTTCAGGCA  TTATTGTCTTGTCCGACGGGCTCTATTCGCACTGAAACTCCACCTACTGACATCGGGGAA  GCTCAAGAGACATTTCCACTTTCATTGGACAAAGACAAACTACCTGGGGTTTTTCATTGT  GGGTTTCATTCCAAGAAATCTTTCGGAGCAACTTCATACTTGATACTTCATCCTGAGGGG  AATATACTTGTTGATAGTCCCAGGTACGTAGAGAAACTTGCTGGGAAGATTGAGAAGATG  GGTGGTGTTCGCTACATGTTTTTGACACACAGGGATGATGTTGCGGATCACAAGAAATGG  GCAGATCGATTCAAGTGTACCAGAATTCTGCATTCTGAAGATGTCCAACCTTCGACCACT  GATGTGGAGTTAAAGCTGGAAGGAAGTGGACCATGGAAACTCTATGAAGATGTCGAGCTT  ATACACACTCCTGGTCACACTGAAGGATCAGTGTGCTTGTTCCATAAACCTCTCAAGGCA  TTATTCACTGGAGACCATCTAACTATGTACGAATCTGGAATGAGCATTATAGAGATGTAC  AACCATTGTTCATTGCCTCTCCAGCTCGAGAGCGTAGAAAGATTGATCAAGCTGGATTTC  AATTGGGTGATACCGGATTCGAATTACCTCATGGCATCCTCTATCTCCCATGATCCTTCT  TCTTCTTCCACGTCTCTCCTTAATCTCCAAACCCAACAATCGATCTTCGGTTACAAGGAC  AAAGTAAAGGACTTTGAGAAAACCCAATTGAGGATTCCAGTTTCTTTCAGAAAGAAAGGT  ATTAACTTGCAAATGATGGCGTCAGGAAAGACACCTGGACTGACTCAGGAAGCTAATGAC  TGCACTTATGAGGCTAATATTGATAGAGATAATAATAACACTGACGTGTTTGACGACATG  AAACAGCGGTTCCTCGCCTTCAAGAGGCTCAAGTACATGGATAACTTAGAACACTACAAA  AAGCTAGCAGATGCTCAAGCTCCAAAGTTTCTAGTGATTGCTTGTGCAGACTCCAGGGTC  TGTCCTTCAGCCGTCCTGGGATTTCAACCCGGTGAAGCATTCACCGTTCGTAACATTGCA  AATTTAGTACCTCCATATGAGTCTGGACCTACTGAGACAAAAGCTGCTCTCCAGTTCTCT  GTGAATACTCTTGAAGTGGAGAACATTTTAGTAATTGGTCATAGCCGCTGTGGAGGGATT  CAAGCTTTAATGGGCATGGAAGAAGTAGATTCCAGAAGTTTCATACATAACTGGGTGATT  GTGGGGAAGAAGGCAAAGGAAAGCACAAAAGCTGTTGCTTCAAACCTCCATTTTGATCAT  CAGTGTCAACATTGTGAAAAGACATCGATAAACCATTCATTAGAAAGACTCCTTGGTTAT  CCGTGGATAGAAGAGAAAGTGCGGAAAGGGTCACTGTCCCTCCATGGTGGGTACTATGAT  TTTGTAAACTGTACATTTGAGAAATGGACTGTCGATTATGAAGGAAGCAGAGGCAGTGGG  ATTGCTGTTAAAAACCGTTGTGTGTTGAAAATATCATTTCGTAATGCTATACCTCTCATT  GAGAGGGCGGAGTGGAGATGGTTGGCGTTGCTGGAGGGAAAGAAGGTGAACCGGGTTTTG  GAAACTGAGGGAATTTTCGGAAACCAAAACCGGGAAATTGAGGAAAACCGGGTGTTGGAT  TGCTTGGAAAACCGGGTTTCGGAAACTGAGGGAAAGGCATGGGGTTGTTTGGGAAACCTT  GCCCTGGGGCTGGGAGTTGAGGAAAACCGGGTTTTGGGAATTGAGGGAATGGCATTGGAT  TTGCTGGGAAACCGGGTCTTGGAAACTGAGGAAAACCGGGTTTAG |
| *BrGLYⅡ2* | Bra031460 | ATGGCATCTTCTTCGACATCTCTGAAAAGGAGAGAGCAGCAGCCAATGTCGCGAGAAGGA  GATCAGCTTATCGTCACTCCTTTAGGCGCCGGTAACGAAGTCGGTCGTTCCTGCGTTTAC  ATGTCCTTCCGCGGCAAAACCATTCTGTTCGATTGCGGGATCAATCCTGCCTACTCGGGA  ATGGCTGCCTTGCCTTACTTCGATGAGATTGATCCTTCCACCATTGATGTCCTCTTGATT  ACTCACTTTCATTTGGATCATGCAGCATCCCTTCCTTATTTTCTAGAGAAGACTACATTC  AAAGGAAGAGTTTTCATGACGCATGCTACCAAGGCTATCTACAAGTTGCTGCTTACAGAT  TATGTTAAAGTCAGCAAGGTTTCTGTGGAAGACATGTTGTTCGATGAACATGACATCAAC  AAATCCATGGATAAAATTGAGGTTATTGATTTCCATCAAACGGTTGAAGTGAACGGCATT  AAGTTCTGGTGCTACACGGCAGGCCATGTTTTGGGTGCAGCCATGTTCATGGTGGACATT  GCTGGTGTCCGGATCCTCTACACAGGCGACTATTCCCGTGAGGAAGACCGACATCTAAGA  GCAGCTGAGCTTCCTCAATTCTCCCCTGACATATGCATCATTGAATCCACTTCCGGCGTC  CAGCTTCATCAGTCTCGTCACATCAGGGAGAAACGCTTCACCGATGTTATCCATTCGACA  GTTGCCCAGGGCGGTCGTGTCCTGATCCCAGCTTTTGCCCTCGGCCGTGCGCAGGAACTC  CTCTTGATTCTAGATGAGTACTGGGCCAACCACCCTGATCTACACAACATCCCTATCTAC  TATGCGTCACCGCTCGCCAAAAAGTGTATGGCTGTTTACCAGACCTACATTTTATCCATG  AACGACAGGATCCGCAACCAGTTTGCGAATTCTAATCCCTTTGTGTTCAAGCACATATCT  GCGCTGAACAGCATCGATGATTTCAGAGATGTTGGTCCGTCTGTGGTTATGGCTAGTCCT  GGTGGTCTTCAAAGCGGTTTCTCGAGGCAGCTCTTTGACATCTGGTGTTCGGATAAGAGA  AACGCTTGTATCATACCTGGTTATATGGTGGAAGGTACACTGGCGAAAACGATAATCAAT  GAGCCCAAGGAGGTGACTCTTATGAACGGTCTTACTGCTCCTCTCAACATGCAGGTGCAC  TACATCTCCTTCTCTGCTCACGCAGACTATGCGCAGACGAGCACTTTCTTGAAAGAGCTC  ATGCCACCAAACATCATCCTTGTCCACGGTGAAGCTAACGAGATGATGAGGCTCAAACAG  AAACTCTTTACTGAGTTTCCTGATGGAAACACGAGGATCATGAATCCGAAGAACTGTGAG  TCGGTTGAAATGTACTTCAACTCTGAGAAAATGGCGAAAACCATTGGGAGATTGGCTGAG  AAGACACCTGATGTTGGAGATTCAGTGAGTGGAATTCTGGTGAAGAAAGGTTTCACTTAT  CAGATAATGGCACCTGATGATCTCCATGTTTTCTCACAGCTATCGACAGCAACTGTTACT  CAGCGGATAACTATCCCTTTCTCTGGAGCTTTCGGTGTGATAACACATCGCCTCGGGAAG  ATTTTCGAGTCCGTTGAATCTTCAACGGACGAGGAAACTGGTCTTCCAGCGCTGAAAGTA  CACGAAAGAGTAACTGTGAAGCAAGAGTCAGAGAAGCACATCTCGCTTCAGTGGTCATCT  GATCCAATAAGTGACATGGTTTCAGACTCCATTGTGGCTCTGGTTCTCAACATCAGCCGG  GAAGTCCCCAAGATCGTTGCGGAGGAAGAAGTTGCTGTGAAATCCGAAGAAGAGAATGGG  AAAAAAGTGGAAAAAGTGATATATGCTCTTCTTGTGTCGCTCTTTGGGGATGTGAAACTT  GGAGAGAATGGGAAGCTGGTGATTAGTGTCGATGGCAATGTCGCTCATCTTGATAAAGAG  AGTGGAGATGTAGAGGGTGAGCATGAAGGTCTAAAAGAAAGAGTGAGAGTAGCTTTTCAT  CGGATTCAAAGCGCTGTGAAACCAATCCCTCTCTCAGCTGAATGA |
| *BrGLYⅡ3* | Bra026637 | ATGGCGATCGATTGCCTCGTACTGGGTGCTGGACAAGAGATAGGGAAGAGCTGTGTAGTA  GTAACGATCAATGGCAAAAAGATAATGTTCGATTGTGGGATGCACATGGGCTGCGACGAT  CACAACCGATACCCAGATTTCTCTATCCTCTCCAAGTCCGGTGATTTCGATAACACCATC  TCCTGTCTCATCATCACTCACTTTCATATGGATCATGTTGGAGCGCTTCCTTACTTCACG  GAGGTCTGTGGGTACAATGGTCCTGTTTACATGTCGTATCCCACGAAGGCTTTGTCTCCG  TTGATGCTTGAGGACTATAGGAGGATTATGGTGGATAGAAGAGGCGAGGAGGAGCTTTTC  ACTTCGGCCCATATCGCTAGTTGCATGAACAAAGTAATTCCATTGGATTTGAAGCAAACG  ATTCAAGTTGATGAAGACCTTCAAATTCGTGCCTATTATGCAGGGCATGTACTTGGAGCA  GTGATGGTGTATGCAAAGGTCGGAGATGCGGCGATTGTGTACACCGGAGATTACAATATG  ACTACAGATAGACATCTTGGAGCAGCGAAAATCGACAGGCTCCAGTTGGATCTTCTCATA  TCAGAGTCTACATATGCAACTACCACTCGCGGCTCAAAATATCCCAGGGAGAGAGAGTTT  CTTCAAGCTGTTCATAAATGTGTTGCTGGCGGAGGGAAAGCTCTGATTCCTTCATTTGCT  CTTGGAAGGGCTCAGGAACTATGCATGCTGCTCGATGATTACTGGGAACGCATGAATATA  AAGGTTCCAATTTACTTCTCGTCAGGTTTGACCATCCAAGCAAACATGTATTACAAAATG  CTCATAAGCTGGACAAGCCAGAACGTCAAAGAAAAGCATGTTACACATAACCCATTTGAT  TTTAAGAATGTTAAAGATTTCTATCGATCTCTTATACATGCACCTGGGCCATGTGTTCTC  TTTGCCACACCCGGTATGCTCTGTGCCGGATTCTCACTAGAAGTGTTCAAGCATTGGGCT  CCTTCACCCCTAAATCTCGTTGCCCTGCCCGGTTACTCTGTGGCTGGTACTGTTGGTCAC  AAACTGATGTCTGGTAAACCAACAACGGTTGATCTCTACAATGGCACCAAGGTTGATGTC  CGTTGCAAGATACATCAAGTGGCTTTCAGTCCTCACACAGATGCAAAAGGAATAATGGAT  CTCACAAAGTTTCTTTCCCCAAAGAACATTGTACTCGTGCACGGAGAAAAACCCAGCATG  ATGTCTCTCAAGGACAAGATAACCTCAGAGCTTGGCATCCCCTGTTTCGTCCCTGCCAAT  GGCGAAACGGTTTCGGTCGCTTCGACCACTTTTGTAAAAGCAAACGCTTCTGATATGTTT  CTTAAAATCTGCTCTAACCCAAACTTCAGATTCTCAAACTCCTCTACTCAGCTCCGTGTT  ACGGACCAGAGAACCGCAGATGGGGTTTTGGTAATAGAGAAGAGCAAAAAGGCAAAAATT  GTTCACCAAGATGAAGTCTCTGAGGTGTTACATGAGAAAGACCATGTGGTCTCTTTGGCT  TATTGTTGTCCTGTCAAAGTTAAGGGAGAATCAGATGATGTTGGTCTGATCAAACAGTTG  TCGGAAAAGATCTCGGAGACAGTGTCTTGTGCTGATGAGATCCATGAATCTGAGACATGT  TTGCAGGTTGGATCTTTCAAGGGTTCTTTGTGTCTGAAAGAGGAGTGTGTGCATAGAAGA  GGGATAAGTAGTAGCTGTAGTGAAGTTAAGTTCTTGTGTTGCAACTGGTCTGTTGCAGAT  TTAGAGCTTGGTTGGGGAATCATCAATGCTATGAAACAAAATCTTTGA |
| *BrGLYⅡ4* | Bra022836 | ATGCAAAACATCTCGAAAGCTTCCTCTGCTATCTCCTTCTTCCGATGCTCTAGGAACCTA  GCAAGTCAGCCATGTGTGAGGCAGCTTCACATCCGAAAGGGTCTTGTCAGTAGAGTCATG  AAGCTTGTCTCTTCACCTCTTAGGACTCTCCGCGGCGCTGGCAAATCTATTCGAGTCTCC  AGTTTCTGCAGTGTCTCCTCCAGTATCTCCTCTTTGCAGATTGAGATGGTGCCTTGTCTT  AAAGACAACTATGCTTACATTTTGCATGACGAGGATACTGGTACTGTTGGGGTGGTTGAC  CCTTCTGAAGCTGAACCTGTGATTGATTCTTTGCAGAGGAGTGGTCGGAACCTAACGTAT  ATATTGAATACACATCATCATTATGATCATACTGGTGGCAATCTGGAATTGAAAGACAGG  TACGGTGCAAAGGTGATTGGCTCAGCTTTAGATAGAGACCGGATACCTGGGATTGATATA  GCCTTGAAGGATGGTGACAAGTGGATGTTTGCTGGTCATGAAGTCCATGTTATGGATACT  CCTGGCCACACAAAAGGCCATATCAGCTTGTACTTTCCAGGATCACGAGCTATCTTCTCT  GGGGACACCTTGTTTAGCTTATCTTGTGGTAAACTCTTTGAAGGTACCCCTAAGCAGATG  CTTGCTTCTCTCAAAAAGATCATTTCTTTACCAGATGACACAAGCATATACTGTGGTCAT  GAATATACACTGAGTAATTCCAAGTTTGCGTTGTCTATAGAGCCAAACAATCAAGTACTT  CAGTCTTATGCAGCTCATGTTGCAGAGCTCCGTAAAAAGAAGTTACCTACGATTCCGACA  ACATTGAAGATGGAGAAAGCTTGTAACCCATTCCTCCGCAGTTCGAATACAGATATTCGT  CAGGCTTTAGGTATTTCAGAGACTGCAGATGAAGCAGAAGCTTTGGCTATTATCCGAGAA  GCAAAGGATAATTTCAAAGCTTAG |
| *BrGLYⅡ5* | Bra000305 | ATGTGGTTATTCTCCATGCCCCTCAAAACTCTTCGTGGAGCTAGAAAAACTCTTAAAGTT  ACTCACTTTTGTAGCATCTCCAACATGCCCTCTTCCTTAAAAATCGAACTGGTGCCTTGT  AGTAAGGACAGCTATGCGTATCTTTTGCACGATGAAGACACTGGGACGGTTGGAGTTGTT  GATCCTTCTGAGGCTGCGCCTGTTATTGATGCCTTGAGCAGGAAGAATTGGAACTTAACT  TATATATTGAATACTCATCATCATGACGATCACATAGGGGGGAATGCTGAGCTGAAAGCT  AGGTATGGCGCAAAGGTGATTGGCTCAGCTGTGGATAAGGATCGGATTCCTGGAATTGAC  ATACTTCTCAAGGATAGTGATAAGTGGATGTTTGCTGGGCATGAGGTTCGGGTAATTGAC  ACTCCTGGTCACACACAAGGCCATATTAGCTTTTACTTTCCCGGGTCAGCCACAATATTC  ACAGGAGACCTGATACATAGCTTATCTTGTGGTACCCTCTCAGAAGGAACCCCCGAGCAG  ATGCTTTCATCACTCCAAAAGATCGTGTCTTTACCAGATGATACAAATATATATTGCGGT  CGCGAAAACACAGCAGGCAATATCAAGTTTGCACTATCTATAGAACCAAAGAATGAAACT  CTTCACTCTTATGCAACCCGAGTCGCCCATCTTCGCAGCCAGGGACTCCCTTCGATTCCA  ACGACTGTTAAGGTAGAGAAAGCGTGTAACCCGTTTCTCAGAACATCCAGCAAAGAAATC  CGTAGATCTTTAAGCATTCCAGACTCGGCAAACGAAGCCGAAGCACTGCGTTGTATTCAC  AGAGCCAGAGATCGTTTCTAA |
| *BrGLYⅡ6* | Bra037715 | ATGCCCCTCAAAACCCTCCGTGGAGCTAGAAAAACACTTAAGATCACACACTTTTGTAGC  ATCTCCAACATGCCCTCTTCATTAAAAATCGAACTGGTGCCGTGTAGTAAGGACAACTAC  GCGTATCTTTTGCACGATGAAGACACAGGCACTGTTGGAGTCGTTGATCCTTCTGAGGCT  GCTCCTGTTATAGAGGCGTTGAGTAGGAAGAATTGGAACTTGACTTACATTTTGAATACT  CATCATCATGATGACCATGTAGGTGGCAATGCTGAACTGAAAGCAAAGTATGGCGCAAAG  GTGATTGGCTCAGCTCTGGATAAGGATAGGATTCCTGGGATTGACATACATCTGAATGAT  GGTGATAAGTGGATGTTTGCTGGACATGAGGTTCGAGTACTTAGCACTCCTGGCAACACA  CAAGGCCATATCAGCTTCTACTTCCCAGGGTCAGCCACAATATTCACAGGAGACTTGTTA  TATAGCTTATCATGTGGTACCATCTCAGAAGGTACCCCTGAACAGATGCTTTCATCACTA  CAAAAGATCGTCTCATTACCAGATGATACAAATATATATTGTGGTCGAGAAAACACAGCA  GGCAATCTCAAGTTTGCATTATCGGTAGAACCAAAGAATGAAACTCTTCAGTCTTATGCA  ACCCGAGTTGCTCATCTTCGAAGCCAGGGACTCCCATCGATTCCAACGACTGTGAAGTTG  GAGAAAGAGTGTAACCCATTCCTCAGAACATCAAGCAAAGACATACGCAAATCTTTAAGC  ATTCCAGAGTCAGCAACTGAAGCTGAAGCACTGCGTCGTATACAGAGAGCAAGAGATCGT  TTCTAG |
| *BrGLYⅡ7* | Bra004763 | ATGTGGTTATTCTCAATGCCACTCAAAACACTCCGTGGAGCTAGAAAAACACTTAAGATT  ACTCACTTTTGTAGCATCTCCAACATGCCCTCTTCTTTAAAAATCGAACTGGTGCCGTGT  AGTAAGGAGAACTATGCTTATATTTTGCACGATGAAGACACTGGCACGGTTGGAGTCGTT  GATCCTTCTGAGGCTGCACCTGTTATAGAGGCGTTGAGTAGGAAAAATTGGAACTTGACT  TATATATTGAATACTCATCATCATGATGATCACATAGGGGGGAATGCTGAGCTGAAAGCA  AAGTATGGCGCAAAGGTGATTGGCTCAGCTGTGGATAAGGATCGGATTCCTGGAATTGAC  ATACTTCTCAAGGAGAGTGATAAGTGGATGTTTGCTGGACATGAGGTTCGGGTTATTGAC  ACTCCTGGCCACACACAAGGCCATGTTAGCTTCTACTTTCCCGGATCAGCCACAGTGTTC  ACAGGAGATTTGATACATAGCTTATCTTGTGGTACCCTTTCGGAAGGTACCCCTGAGCAG  ATGCTTTCATCATTCCAGAAGATTGTTTCTTTACCAGATGATACGAATATATACTGCGGT  CGTGAAAACACATCAGGGAATGTCAAGTTTGCACTATCCATAGAACCAAAGAATGAAACT  CTTCGGTCTTATGCAACCCGAGTCGCCCATCTCCGCAGCCAAGGGCTCCCCTCGATTCCA  ACGACTGTTAAGGTGGAGAAAGCGTGTAACCCATTCCTCAGAACATCAAGCAAAGAAATC  CGCAGATCTTTAAACATTCCAGAGTCAGCAAACGAAGCTGAAGCATTACGTCGTATACAC  AGAGCAAGAGACCGTTTCTAA |
| *BrGLYⅡ8* | Bra018252 | ATGAAGCTCGTCTCTTCACCCCTTAGGACTCTACGCGGTGCTAGTAAATCTATTCGTGTC  TCCAATTTCTGCAGTGTCTCCAACCTCTCCTCATTACAAATCGAACTGGTGCCTTGTCTT  AACGACAACTATGCTTACATTTTACATGACGAGGATACTGGTACAGTTGGTGTGGTTGAC  CCTTCTGAAGCTGAACCTGTGATAGAATCATTACAGAGGAGTGGTCGAAACCTAACGTAT  ATATTAAATACACATCATCATTATGATCATACTGGTGGCAATCTGGAATTGAAAGACAGG  TACGGTGCAAAGGTGATTGGCTCAGCTGTAGACAGAGACCGCATTCCTGGAATCGATATA  GCATTGAAGGATGGTGACAAATGGATGTTTGCTGGTCATGAAGTCCATGTTATGGATTCT  CCTGGCCATACAAAAGGCCATATCAGTTTATACTTCCCAGGATCACGAGCTATCTTCACT  GGGGACACCTTGTTTAGCTTGTCTTGTGGTAAACTCTTTGAAGGTACACCTAAGCAGATG  CTTGCGTCTCTCCAAAGGATCATATCTTTACCAGACGACACGAGCATATACTGTGGTCAT  GAATATACACTGAGTAATTCAAAGTTTGCCTTGTCCATAGAGCCAAACAACGAAGTACTC  CAATCTTATGCAGCTCATGTTGCAGAACTCCGTCAAAAGAAACTACCCACGATTCCGACA  ACAGTGAAGATGGAGAAAGCTTGTAACCCGTTCCTCCGAAGTTCGAATACAGATATTCGT  CGGGCGTTAGGCATTTCAGAGACTGCAGATGATGCAGAAGCTTTAGGGATTATCCGAGAA  GCTAAGGATAATTTCAAAGCTTAG |
| *BrGLYⅡ9* | Bra029872 | ATGAAGATCTCCCACGTTCCCTGTCTAGAAGACAACTACTCCTACCTGATAATCGACGAG  AGCACCGGAGACGCGGCGGTTGTGGATCCAGTTGAGCCCGAGAAGGTAATCAAGTCGGCT  GAGCAGCACAGTGCCAATATCAAGTTCGTACTCACCACCCATCATCACTGGGATCATGCC  GGTGGCAACGAGAAGATGAAGCAGTTGGTGTCTGGAATCAAAGTCTATGGAGGCTCTCTT  GATAAGGTCAAGGGATGCACTGATGCTGTCGATAATGGTGACACCTTGTCTTTGGGTCAA  AATATTAACATATTAGCTCTTCACACCCCTTGTCACACCAAGGGTCACATTAGTTACTAT  GTCACAGGCAAAGATGGAGAAACCCCAGCTGTGTTCACTGGAGATACACTTTTCGTTGCT  GGCTGTGGGGAGGTTTTCGAAGGGACAGCTGAACAGATGCATCAGTCCTTGTGTGTGACT  CTGGCTTCATTACCTAAACCGACCCAGGTTTACTGCGGCCACGAGTACACTGTGAAGAAC  TTGGAGTTTGCTTTAACTGTGGAACCAAACAACGAGAAGATACAGCAGAAGCTATCATGG  GCCCGTCAACAGCGCCAAGCGAATCTTCCCACAATCCCTTCAACACTAGAGGAAGAGCTC  GAAACAAACCCGTTTATGCGCGTTAATAATCCTGAGATACAGGAGAAACTTGGTTGCAAA  TCACCGATCGATACTCTCAGAGAAATCAGGAACAAGAAGGATCAGTGGAGGGGCTAA |
| *BrGLYⅡ10* | Bra039681 | ATGGGTTCATCTTCCTCTTCCTCCTCCTCCTCCTCCTCGAAGCTTCTCTTCCGTCAGCTC  TTTGAGAAAGAGTCTTCCACTTTCACGTATCTCCTCGCCGACGTGTCTCATCCCGATAAG  CCTGCTCTGTTGATTGATCCGGTGGACAAGACTGTTGAAAGAGACTTGAAGCTGATCAAT  GAGCTAGGCTTGAAGCTTGTCTATGCTATGAACACTCATGTTCATGCTGATCATGTCACT  GGCACTGGACTTCTTAAGACAAAGGTACCCGGTGTGAAGTCTGTTATTTCAAAAGCAAGT  GGTTCCAAAGCAGATATGTTTCTTGAGCCTGGTGACAAAGTATCCATTGGTGATATATAC  CTTGAGGTTCGTGCTACACCTGGACATACTGCAGGATGTGTTACATATGTGACTGGAGAG  GACGCGGATCAGCCCCAACCAAGAATGGCTTTTACCGGGGATGCTGTCCTTATCCGCGGT  TGTGGGAGAACTGACTTTCAGATATTTACATTGCCAAAGGACACATTGATCTATCCAGCT  CATGACTACAAAGGCTACGAGGTAAGTACAGTTGGAGAAGAGATGGAACACAACCCACGT  TTAACCAAAGATAAAGAAACATTCAAATCCATCATGTCTAATCTGAATCTAGCGTATCCG  AAGATGATTGATGTTGCAGTTCCAGCAAACATGGTGTGTGGATTACAAGATTTGCCTCCT  CAAGCCAACTTATAA |
| *BrGLYⅡ11* | Bra038629 | ATGAAACCTGCTGCCTCTTTGCATGGTTACCCTTCTTCCCCTATCTACTTCGATGCTCGC  AGACCAGTTCCTACTCCTCCCTCCAAAATGGCAGCTTTCAGTGCTCTATCGCTTTCTCCT  TACTCTTTCACCTTCCGACAAAGCTCTCCAGTTAGGTCTACCGTTTCGTGTTCCGTCACT  TCTCCTCCTGCCTCTTCTGGTACTTCTTCTTCTTCTTCTTCTTCCAATAAGACACCTCGT  AGAAGATCTGGTAGACTAGAAGGAGCGGGGAAAAGCATGGAGGACTCTGTGAAACGTAAA  ATGGAACAGTTTTATGAAGGAACCGATGGACCTCCGCTCCGTGTCCTTCCCATAGGTGGC  CTTGGTGAGATCGGGATGAACTGTATGCTTGTTGGCAACTATGATCGTTACATTCTAATC  GACGCCGGTATTATGTTCCCTGATTATGATGACCCTGGGGTCCAGAAAATTATGCCAGAC  ACAGGGTTTATCAGACGATGGAAACACAAGATTGAAGCTGTTGTTATAACGCATGGTCAT  GAAGATCACATTGGTGCCTTGCCTTGGGTTATCCCAGCTTTGGACTCTAATACACCAATA  TTCGCATCATCCTTTACCATGGAGCTTATAAAGAAGCGCTTGAAGGAGCATGGGATCTTT  GTTCAGTCTAGGCTCAAGACATTTAATACTCGAAGGAGATTTATGGCTGGACCATTTGAA  ATAGAACCCATTACAGTTACTCACTCTATTCCTGATTGTAGTGGTTTAGTCCTCCGTTGC  GCTGATGGTAATATTCTTCACACCGGAGACTGGAAGATTGATGAAGCACCATTGGATGGA  AAAGTCTTTGATCGTGAGGCTTTAGAGGAACTCTCTAAGGAAGGAGTCACGTTGATGATG  AGTGACTCAACAAATGTATTGTCACCGGGAAGGACAACTAGCGAAAAAGTGGTAGCAGAT  GCTCTGGTGAGGAATGTAATGGCGGCCAAGGGAAGAGTTATCACAACTCAGTTTGCCTCC  AATATACACCGTTTAGGAAGTATTAAGGCTGCTGCTGATTTAACTGGTCGAAAGTTGGTC  TTTGTTGGCATGTCCTTGAGGACATATCTAGAAGCAGCTTGGAAGGATGGAAAGGCTCCA  ATTGACCCGTCAAGTTTGGTGAAAGTTGAAGATATTGAAGCATATTCTCCTAAGGACTTA  TTGATCGTCACGACTGGATCACAAGCGGAACCACGCGCTGCCCTGAATCTTGCGTCATAT  GGAAGTAGTCATGCTTTCAAACTTACCAAGGAAGACATAATACTTTACTCAGCCAAGGTA  ATCCCAGGCAATGAATCAAGAGTAATGAAGATGATGAATCGGTTAGCAGATATCGGTCCA  AAAATTGTCATGGGTAAAAATGAAATGCTGCACACATCTGGTCATGCCTACCGTGGAGAG  TTGGAAGAGGTTCTTAAAATAGTGAAACCCCAGCATTTTCTCCCCATACATGGAGAACTT  TTGTTTCTCAAGGAGCATGAGTTGCTCGGGAAGTCTACTGGGATTCGTCACACTACTGTT  ATAAAGAATGGAGAGATGCTTGGAGTTTCTCACTTAAGAAATAGAAGAGTTTTGTCCAAT  GGATTTAGCTCTCTTGGGAGGGAGAACTTGCAGTTAATGTATAGTGACGGTGATAAGGCA  TTTGGCACAGCAAGTGAACTCTGTATTGACGAGAGACTCAGAATATCATCGGATGGCATT  ATAGTTCTGAGCATGGAAATCATGCGCCCGGGCGCCTCGGAGAACACTTTGAAAGGGAAG  ATAAGAATCACAACGCGATGTATGTGGCTTGACAAAGGAAGACTATTAGATGCACTTCAC  AAGGCAGCACATGCTGCTCTATCAAGTTGTCCTGTGAACTGTCCCTTGTCTCACATGGAA  AGAACAGTCTCCGAAGTCCTGAGGAAGATTGTGAGGAAGTACAGTGGTAAAAGGCCTGAA  GTCATTGCCATAGCCATGGAAAATCCCATGGCGGTCCGAGCTGATGAGGTCAGTGCGAGG  ATGTCCGGGGATCCAAATCTTGGCTCTGGAGTTGCAGCGTTAAGGAAAGTTGTGGAAGGA  AACCATAAAAGAAACCGAACCAAGAAAGCACCTTCGCAAGAAGAAGCTGGGGAGATAATT  GATAGTGCAGGACTACTAGCTGAGGAAGGAACCGCTTCGTCGACATACACAGAAGGTGCT  GAAGATGTGCCTGTTCGGAGTTCTTCTGAAGAATCGGATGATTTTTGGAAATCATTCATC  AATCCATCATCACCACCTTCACCTGATGAAACCAAAAACGTGGATAAGTCACCTGATGCA  GAGACTAAAACAGAGGATAGCGAAAGCAGCAGAGAAGAGGAGGATGATGATAATACATCT  GATTCTCAAACCAAGTCGTCAACAAAACGTGTGAGGAGGAACAAATGGAAGCCTGAGGAA  GTTAAGAAGGTGATCAGAATGCGTGGAGAGTTGCACAGCAGGTTCCAAGTGGTGAAAGGT  AGAATGGCTTTGTGGGAAGAGATCTCTTCAAATCTATCTGCTGAAGGAATCAATCGAAGC  CCGGGACAGTGCAAGTCTCTGTGGGCGTCTCTTATTCAGAAATACGAGGAGTGCAAGGCG  GATGAAAGAAGCAAGACGAGCTGGTCACATTATGAGGACATGAACAGCATTTTGTCTGAG  TTAGACACACCTGCGCCTAAGTAA |
| *BrGLYⅡ12* | Bra009712 | ATGGGTACTTCGGTGCAGGTTTCTCCACTGTGCGGAGTGTACAACGAGAATCCACTCGCA  TACTTGGTCTCCATCGATGGCTTCAACTTCCTCCTCGACTGTGGTTGGAACGACCTCTTC  GACCCTTCCCTCCTCGAACCTCTCTCCAGGGTTGCTTCTACCGTAGATGCGGTTTTGCTT  TCTCATCCAGATACGCTTCACCTCGGTGCTCTTCCTTATGCCATGAAGCAGCTTGGACTC  TCTGCTCCTGTTTACGCCACTGAGCCTGTTCACAGACTAGGTCTCCTCACTATGTACGAT  CAGTATCTATCCAGAAAGCAAGTCTCCGACTTTGATCTGTTCACGCTGGATGACATTGAT  TCAGCTTTCCAGAATGTCATCAGATTGACTTACTCTCAGAACTTCCATCTTTCTGGGAAG  GGAGAGGGTATTGTAATTGCTCCTCATGTTGCTGGACATATGCTGGGAGGTAGTATCTGG  AAGATAACAAAGGATGGGGAGGAGGTTGTGTATGCTGTTGACTACAATCATCGGAAAGAA  AGGCATTTAAACGGAACTGTTTTACAGTCTTTTGTTCGACCTGCTGTTCTGATTACCGAT  GCGTACAATGCTCTTTATACCAATCAAACCCAAAGCCATCACAGGGACACAGAATTTCTT  GATACCATTTCAAAGCATCTGGAAGTTGGGGGCAATGTTTTATTGCCAGTAGATACTGCG  GGTCGAGTTCTGGAACTTCTCTTGATACTTGAACAGCATTGGTCACAGAGAGCTTTTAGC  TTTCCCATTTACTTTCTCACATATGTGTCATCTAGCACAATTGACTATGTTAAGAGCTTC  CTCGAGTGGATGAGTGACTCCATTTCAAAGTCCTTTGAGACTTCACGTGATAATGCCTTT  CTATTGAGGCATGTCACTCTCTTGATAAACAAGACTGATCTGGATAATGCTCCACCTGGT  CCAAAGGTTGTTCTTGCGTCCATGGCTAGTCTTGAAGCTGGTTTTGCTCGAGATATATTT  GTGGAATGGGCCAATGATCCCAGAAATTTAGTCCTCTTTACTGAAACAGGCCAGTTTGGC  ACTTTAGCTCGTATGCTTCAGGCAGCCCCACCTCCCAAATTTGTTAAAGTCACCATGTCT  AAGAGGGTTCCTTTGGCCGGGGAAGAGTTGATTGCATATGAAGAGGAGCAAAACAGACTT  AAGAGAGAGGAAGCCTTGCGAGCTAGCCTCGTCAAAGAGGAGGAAACAAAAGCTTCCCAC  GGACCTGATGATAACTCAAGTGAACCAATGGTCATAGATACCAAGACTACTCACGATGTT  GTTGGGTCTCACGGACCTGCATATAAAGATATATTGATAGATGGATTTGTTCCCCCATCC  AGCAGCATTGCCCCAATGTTCCCATTCTATGATAACACTGCTGACTGGGACGAGTATGGG  GAGGTCATTAATCCAGATGACTACGTGATCAAGGATGAGGACATGGACCGAGGAGCAATG  CATGCAGGAGGTGATGTGGATGGAAGGCTTGATGAGGCAACTGCTAGTCTCATGCTAGAT  ACTAGACCTTCGAAAGTCATATCCAATGAGCTAATTGTGACTGTTAGTTGTTCACTGGTT  AAAATAGACTACGAAGGTCGTTCGGATGGCCGCTCAATCAAGTCAACGATTGCACATGTT  TCTCCTCTGAAACTTGTTCTGGTGCACGCAACAGCAGAGGCTACGGAGCATTTGAAGCAA  CACTGCTTGAACAGCATCTGTCCGCACGTGTACGCTCCGAAAATAGAGGAGACCATTGAT  GTGACTTCTGATCTATGTGCCTATAAGGTCCAACTCTCTGAGAAGCTGATGAGCAATGTG  ATCTTCAAGAAGCTGGGAGATTCAGAAGTAGCATGGGTGGATTCAGAGGTAGGGAAGACA  GAGAGCGAGAAGCGGTGTCTGCAACCAATGGCAAGTGCAGCAGCTCCTCACAAACCCGTG  CTAGTGGGAGATCTGAAAATGCAAGACTTCAAGCAGTTTCTGGCAAGCAAAGGAGTTCAG  GTAGAGTTTGCAGGTGGAGGAGCTTTACGATGCGGTGAGTATGTCACTCTAAGGAAGGTC  GGTCCAACTGGTCAGAAGGGGGGAGCATCAGGGCCACAGCAAATTCTGATAGAAGGACCG  TTGTGTGAAGATTATTATAAGATCAGGGATTATCTATATTCTCAGTTTTACCTCCTCTGA |
| *BrGLYⅡ13* | Bra030931 | ATGGGTTCGTCTTCCTCTTCCTCCTCGAAGCTTCTCTTCCGTCAGCTCTTCGAGAAAGAG  TCTTCCACTTATACGTATCTTCTCGCCGACATTTCCCATCCGGACAAACCTGCTCTGTTG  ATTGATCCTGTGGACAAAACTGTCGATAGAGATTTGAAGCTGATCAATGAGTTAGGATTG  AAGCTTGTCTATGCTATGAACACTCATGTTCATGCTGATCATGTAACTGGAACTGGTCTT  CTTAAGACAAAGGTCCCAGGTGTGAAGTCCGTAATCTCAAAAGCAAGTGGTTCCAAAGCA  GATAAGTTTGTTGAACATGGAGAGAGAGTATCTATTGGTGATTTATACCTCGAGGTACGT  GCTACACCTGGACATACAGCAGGATGTGTTACATATGTGACTGGAGAAGGAGCTGATCAG  CCCCAACCAAGAATGGCTTTTACCGGCGATGCTGTACTGATCCGCGGTTGTGGGAGAACC  GACTTTCAGGGTGGATGCTCGGATCAACTCTATGAGTCTGTGCACTCACAGGCAACTCAT  GACTACAAAGGTTTCGAGGTAAGTACAGTTGGAGAAGAGATGCAACACAACCCGCGTTTA  ACTAAAGACAAAGAAACATTCAAATCCATCATGTCAAATCTGAATCTGCCGTATCCGAAA  ATGATTGATGTTGCATTACCAGCAAACATGGTGTGTGGATTACAAGACCTGCCTTCTCAA  GCCAACTAG |
| *BrGLYⅡ14* | Bra024757 | ATGGGCAATCTCAATCTCGCGGTGATCATCAAGAACCCTGGAGACTCCGCCCAGTTCCTC  CTCGAGAAGCAAAAACAGCCGCCAAAATTTGGGGACGAAGCTTACGACTCCTACGTCGAT  TCCAATCTCTGGGACCTCCCATCAGCAGATCTACCGTCTCTAGAAGACGGAACAAGGTCC  GTAAATGCTCTCTCTATAGCTGAATCATGCTCAGAAGAGATCGATTTGAAGAATTTCGAC  TTAGACTCTACCCTGATTCGACTGTTGGCGAGTTTAGGGATTGAGTTTAGCGATGTGGGA  GAGTGGAGTTTCGTCAGGTATGTAGTGGAGCCTGAGTTTGGACCTGACTCATGCGTCCCT  ACTTGTTTTCTCTCCGGGAAGCTGTTGGATACAGACAAGAGTTTACAAGACAACTGTAAG  TGGATGTCCATGGAAGCTTGCTTTGACTGTCTTTTAGATGCAAAACCGGGTGGTGATCGT  GTTGGACCATTAGTGCTACTCGGGCTTGGGGATGGTTCCATGAAGCAGAAGTTGGCACCT  TCTCTACCTGTCCAGGAATATCCACCCGGTGTTATGATTGTGCCAATGCGTAGCCGGACA  TTGAAACCTTTCACGACTACTAATCTGGTTGTGTTTGCTCCTGAAAATGTTTCAGTGGAC  GATCAAGAGAGAGATTTTGTAATTCATGGGGATGCGTTGATTGTGGATCCTGGATGCCAC  TACAAACTCCACATTGAGCTCAAGAAAATTGCTGATGCTTTACCTAGAAAGCTAATTGTC  TTTGTTACACATCACCATCGTGATCACATTGGTGGTCTTTCTGCTATACAAGAAAGCAAT  CCTGATGCTATTCTAGTGGCACATGTCAAAACTAGGAATCGCATTGATGGTTGGTCTGGT  AACTATACCCCAGTTTCTGGTGGAGAAAACATCTATGTCAATGGTCAGAGTTTGACCGTC  ATTTTTGCTCCGGGACACACAGATGGCCATATGGCACTACTTCATAACTCCACTCGTTCT  CTGATTGTTGGTGATCATTGTGTTGGTAAAGGAAGTGCTTTCTTGGACATAAGGTCAGGT  GGGAACATGACGGAATACTTTCAAACGACATATAAGTTCCTGGAGCTTTCTCCACATGTA  GTCATTCCCATGCATGGAAGGGTCAATCTGTGGCCAAAACACATGCTCTGCGGATATCTC  AAAAACCGCAGGAGCAGAGAAGAATCAATCCTAAAGGCCACTGAGGATGGAGCTCAGACA  TTGTTTGACATAGTTTCCAATGTGTACTCAAAAGTGGATCGCAGTTTCTGGTTGGCTGCA  GCGTCAAACGTGAGGCTGCATATCGACAACCTAGCTGTAGAAAACAAATTACCAGAGGGA  TTCTCAATCCAGAAGTTTAAAGCAAGTTGCGGATTTAGTTTTGTGGTACGGTGGGCTGCG  GGTTATATCGGTAGCCGGATTCCATTCAAGATCAATAAGCCAGGTTTAATTATGTCAGTG  ATAGCTGCAGGAGCTGGTTATTTTCTTCTCTACACTTGCAAAAAGAAGAACACTATTGAA  TCTTGA |
| *BrGLYⅡ15* | Bra032436 | ATGCAAGCTATCTCGAAAGTTTCTTCTGCAGCCTCCTTGTTTCGATGTTCTACGAAGCTA  ACAAGTCAGCCATGTATGAGCCAGCTTAGCCTCAGAAAAGGACTTGCCTCTGGTGTGATT  AAGTTGTTCTCTTCGCCTCTCAAGACTCTGTGCGATGCTGGAAGATCTGTCCACGTGTCA  AGATTCTGCAGTACCTCTAATATCTCTTCCTCATTGCAAATTGAACTGGTGCCATGTCTC  GCCGACAACTACGCTTATATATTGCACGACGAGGAGACTGGTACAGTTGGTGTGGTTGAC  CCTTCTGAAGCTGTTCCCGTTATGAATGCCTTAAAGCAGAATGGTCAAAACTTAACATAT  ATACTGAATACACATCACCATTATGATCACACTGGTGGGAACTTGGAATTAAAAGACAAG  TATGGTGCAAAGGTGGTCGGCTCAGCTGTAGATAGTAAACGGATTCCTGGAATTGATATA  GCTCTGAAAGATGGTGATAAGTGGGAGTTCGCTGGCCATGAAGTCCATGTTATGGAAACT  CCTGGCCACACAATAGGCCATATTAGTTTCTACTTTCCAGGGGCACGAGCAGTTTTCACT  GGGGACACCTTATTTAGCTTATCGTGTGGTAAGCTCTTCGAAGGTACTCCAGAGCAGATG  CTAGCTTCGCTCCAGAGGATAGTTGCTTTGCCAGATGACACAAGCATATATTGTGGCCAT  GAATACACACTGAGTAACTCCAAGTTTGCGTTATCTATAGAACCAACTAACGAGGTACTC  CAGTCTTACGCAGCCTACGTTGCAGAGCTCCGTGGCAAGAAGTTACCTACGATTCCAACG  ACGGTGAAAATGGAGAAAGCGTGCAACCCGTTTCTCCGCACTGGGAACATGGATATTCGC  CGGGTTCTAGGTGTACCAGAGACTGCAGATGAAGCAGAAGCTCTGGGCGTTATAAGAAGA  GCAAAGGACAATTTTAAAGCTTAA |
