# Supplementary material for: Genome-wide analysis and expression profiles of glyoxalase gene families in Chinese cabbage (Brassica rapa L)
Source: PLoS One. 2018 Jan 11;13(1):e0191159. doi: 10.1371/journal.pone.0191159 (PMC5764358; doi:10.1371/journal.pone.0191159)
Supplement: S3 Table — (DOCX) [file pone.0191159.s005.docx]

**S3 Table. The amino acid sequences of *BrGLY* genes in *B. rapa***

| **Name** | **Locus**  **Identifier** | **Amino acid sequences** |
| --- | --- | --- |
| ***BrGLYI*** | | |
| BrGLYI1 | Bra008491 | MKENTGNPLHLKSLNHISLLCRSVEESMSFYQHVLGFLPIRRPGSFDFDGAWLFGHGVGI  HLLQSTEPEKLLKKTEINPKDNHISFQCESMGAVEKKLKEMGIEYVRAVVEEGGIQVDQL  FFHDPDGFMIEICNCDSLPVVPLAGEMARSCSRVNIHQLVQPPQIHP |
| BrGLYI2 | Bra006835 | MASIFRPSISLDLRPKVTCTNLSTKERLDFHNKSLRKEKLNIRLRGVKANQAQGTSVVTE  EKELNNKTDYGVVGVHHVGLLCENLERSLEFYQNILGLEINEARPHDKLPYRGAWLWVGS  EMIHLMELPNPDPLTGRPEHGGRDRHACIAIRDVSVLKEILDKAGIAYTMSKSGRPAIFT  RDPDTNALEFTQV |
| BrGLYI3 | Bra005612 | MATLGHIARESSDVTRLALFYKEVFGFEEIESPDFGDLKVIWLNLPGAFAMHIIQRNTST  TLPEGPYSATSAVRDPSHLPMGHHICFSVSNFDSFLRSLKEKGIETFQKSLPDGKVKQVF  FFDPDGNGLEVASRSES |
| BrGLYI4 | Bra018692 | MASNVIKPAYAYTVVYVRDVAKSVEFYSRAFGYNVRRLDESHRWGELESGQTTIAFTPLH  QHETDDLTGKVQSSTRSERERAPLEVCFCYADVDAAFKRAVENGAVAVSEPEDKEWGQKV  GYVRDIDGIVVRIGSHVKSYKPWTSFGFSLAINLNLPVVPQLNNITSKKRQ |
| BrGLYI5 | Bra018654 | MGSYSIASAISRVSVSPLNRFVKLYSTAFSYINCPCNRALRPKRFDQLRVFSMASEAKES  AANNPGLSTVRDEATKGYIMQQTMFRVKDPKASLDFYSRVLGMSLLKRLDFSEMKFSLYF  LGYEDTSTAPTDPTERTVWTFGRPATIELTHNWGTESDPEFKGYHNGNSEPRGFGHIGVT  VDDVHKACERFEQLGVEFVKKPNDGKMKNIAFIKDPDGYWIEIFDLKTIGTTAGNAA |
| BrGLYI6 | Bra019830 | MALRCFPIWACPQTAYYHYPLLGLDTKRRRIPLWECSSSASQRAVTAVGSEVPYGRELKK  PSDEMGLTQESPQLETFHRDLSMLPKPLTANSLTSSAGDDSKVRISFQGIPGAYSETAAL  KAYPNCETVPCDQFETAFQAVELWLVDKAVLPIENSVGGSIHRNYDLLLRHRLHIVQEVH  LPINHCLLGVPGVSIEDIKCVLSHPQALDQCVNSLNDLGIQRVSAKDTATAAQTVSSSGE  RSIGAVASVRAANIYGLDILVENIQDDANNVTRFLILARDPMIPRTDRPYKTSIVFSLEE  GPGVLFKALAVFSLRNINLSKIESRPQRRRPLRVVDGSNNGCAKYFDYLFYIDFEASMAE  TRAQHALGHLQEFTSFIRILGCYPMDLVSSSREVVGQLETDCFSSDGRSKSSGICSPRAS  SLRKLADVASLDWPKNDTRRFFHVVYRVGDLDRTIKFYTECFGMKVSRQRDVPKEKYSNA  FMGFGSEKSHFAVELTYNYGVSSYDIGDGFGHFTISTQDVYKMVETVRAKGGNVTREPGP  VEGGSSIIAIVKDPDGYPFELIQRGPTPEPFCQVMLRVGDLDRAIKFYEKALGMRLLRRI  KKPEYKYTIGMMGFNESVVLELTYKYGVTEYKKGNAYAQIAIGTDDVYKSGEVVKIVNKE  LGGKITREPGPLPGIGTKIVSFLDPDGWKTVLVDNKDFMKELGESSVNYVSSSVKNLEEA  PEAKTKSQSFEEEEEKKKMRGGSLWQLGQSITRRLAQSDKKPLSRRYLASGADLKKTALY  DFHVAHGGKMVPFSGWSMPIQYKDSIIDSTVNCRVNGSLFDVAHMCGLSLKGKDCVPFLE  KLVVADVAGLAPGTGSLTVFTNEKGGAIDDSVITKVTDEHIYLVVNDGCRDKDLAHIEEH  MKAFKSKGGPLAAPLLQHLTKEDLSKLYFGQFQILDINGSTCFLTRTSSRGRVQEDLTVN  ESLCFGFTAKAILEKSEGKVRLTGLGARDSLRLEAGLSLYGNDMEQHISPVEAGLTWAIG  KRRRAEGGFLGADVILKQLEDEPTIRRVRFFSSGPPARSHSEVHDENGNKIGEITSGGFS  PNLKKNIAMGYVKSGQHKTGTKVKILVRGKPYEGNITKMPFVANKYYKPS |
| BrGLYI7 | Bra026138 | MKENAGNPLHLTSLNHVSLLCRSIEESMVFYQTVLGFFPIRRPESLNFEGAWLFGHGIGI  HLLRSSEPEKLPKKTEINPKDNHISFQCESMSAVEKKLEEMEIEYVRAIVEEGGIQVDQL  FFHDPDGFMIEICNCDSLPVVPLIGGMARSCSRVKLHQMVQPQQQTQIHQVVHP |
| BrGLYI8 | Bra011950 | MEQKNKSDESRPPLMALNHVSRLCRDVKKSLEFYTKVLGFVETERPASLDFDGAWLFNYG  VGIHLVQAKDEEKLPSNTDHLDPMDNHISFQCEDMEALEKRLKEVDVKYIKRTVGEQEDA  AIDQLFFNDPDGFMVEICNCENLELKPRDSADAIRLPGDRHAPPVSLPGSSDHADDTRLP  QTNS |
| BrGLYI9 | Bra004214 | MRIISTASTIRPSLLGCVSASSPRFPVVSRNLSFSHVTQSKLLTLRRSVSCLGVAESGKA  STAATEEDLLKWVKDDNRRMLHVVYRVGDLDRTIKFYTECLGMKLLRKRDIPEEKYTNAF  LGYGPEDSHFVIELTYNYGVDKYDIGAGFGHFGIAVDDVAKTVELIKAKGGKVTREPGAV  KGGKTVIAFIEDPDGYKFELLERGPTPEPLCQVMLRVGDLDRSIKFYEKAFGMELLRTRD  NPEYKYTIAMMGYGPEDKTAVLELTYNYGVTEYDKGNAYAQIAIGTDDVYKTAEAVKLFG  GKITREPGPLPGISTKITACLDPDGWKSVFVDNVDFLKELE |
| BrGLYI10 | Bra016662 | MKENAGNPLHLTSLNHVSLLCRSIEESMNFYQKVLGFFPIRRPESLNFEGAWLFGHGIGI  HLLRALELEKLPKKNEINPKDNHISFQCESMGAVEKKLDEMEIDYVRSKVEEGGIQVDQL  FFHDPDGFMIEICNCDSLPIVPLVGGMVRSCSRVKLHQMVQPQPQTQINQVVHP |
| BrGLYI11 | Bra016811 | MAENADLLEWPKKDKRRFLHVVYRVGDLDRTIQFYTECFGMKLLRKRDVPEEKYSNAFLG  FGPETSNFVVELTYNYGVSSYDIGTGFGHFAISTQDVSKMVEAVRAKGGNGTREPGPVKG  GGSVIAFVKDPDGYMFELIQRGPTPEPLCQVMLRVGDLDRAIKFYEKALGMRLLRRIERP  EYKYTIGMMGYAEEYESIVLELTYNYGVTEYTKGNAYAQIAIGTDDVYKSAEVVKIANQE  LGGKITREAGPLPGLGTKIVSFLDPDGWKTVLVDNEDFLKELE |
| BrGLYI12 | Bra026768 | MKENAVNPLRLTSLNHVSLLCRSLEESMNFYQKVLGFFPVRRPESLDFEGAWLFGHGIGI  HLLRSTEPEKLPKKTAINSKDNHISFQCESMAAVEKKLDEMEIEYVREIVEGRGIKVDQI  FFHDPDGFMIEICNCDSLPVVPLVGGLAQYCAKVKLHQMGQPQPQTN |
| BrGLYI13 | Bra031589 | MASNIMRPAFAYTVVYVKDVAKSVEFYSRAFGHNVRRLDESHRWGELESGQTTIAFTPRH  QHETDDLTGKVQATHSDPERAPIEVCFCYPDVDAAFKRAVENGAVAVSEPEDKEWGQKVG  YVRDIDGIVVRIGSHVK |
| BrGLYI14 | Bra032415 | MVHASYLLTPGDLRFLFTAPYSPSLSAGETRTSATASIPSFDHVSCRSFFSSHGLGVRAV  AIEVEDAESAFSISVANGAVPSSPPNVLNGAVTIAEVKLYGDVVLRYVSYHNGAVNFLPG  FESVDDTSSFPLDYGIRRLDHAVGNVPELGPALTYLAGFTGFHQFAEFTADDVGTAESGL  NSAVLASNDEMVLLPVNEPVHGTKRKSQIQTFLEHNEGAGLQHLALMSEDIFRTLREMRK  RSGVGGFDFMPSPPPTYYKNLKKRVGDVLSDEQIRECEELGILVDRDDQGTLLQIFTKPL  GDRPTIFIEIIQRVGCMKKDEEGKVYQSGGCGGFGKGNFSELFKSIEEYEKTLEAKQLVG |
| BrGLYI15 | Bra015511 | MGHENAAVSENQHHDDAATTSASPGFKLVGFSKFVRKNPKSDKFKVKRFHHIEFWCGDAT  NVARRFSWGLGMRFSAKSDLSTGNMVHASYLLTSGDLRFLFTAPYSPSLSAGENPPTTTA  SIPSFDHVTYRSFFSSHGLGVRAVAVEVEDAEAAFSISVSNGAVPSSPPIVLNDAVTIAE  VKLYGDVVLRYVSYKVATVFLPRFETVDDTSSFPLDYGIRRLDHAVGNVPELGPALTYLS  RLTGFHQFAEFTADDVGTAESGLNSAVLANNDETVLLPVNEPVHGTKRKSQIQTYLEHNE  GAGVQHLALMSEDIFRTLREMRKRSGVGGFDFMPSPPPTYYKNLKNRVGDVLSEEQIEEC  EELGILVDRDDQGTLLQIFTKPLGDRPTIFIEIIQRIGCMKKDEEGRVYQSGGCGGFGKG  NFSELFKSIEEYEKTLEAKQLVG |
| BrGLYI16 | Bra002767 | MASIFRPSVSLDLRPKVSCTNHLPAIERFEFQKNKNLRKDRLNGILKANQAHGSAEGISV  VQEKEINNQTDYGVVGVHHVGLLCENLERSLEFYQNILGLEINEARPHDKLPYRGAWLWV  GSEMIHLMELPNPDPLTGRPEHGGRDRHACIAIRDVSYLKEILDKAGIEYTMSRSGRPAI  FTRDPDANALEFTQV |
| **BrGLYII** | | |
| BrGLYII1 | Bra011454 | MSAVIKQPTCKEERMNALQALLSCPTGSIRTETPPTDIGEAQETFPLSLDKDKLPGVFHC  GFHSKKSFGATSYLILHPEGNILVDSPRYVEKLAGKIEKMGGVRYMFLTHRDDVADHKKW  ADRFKCTRILHSEDVQPSTTDVELKLEGSGPWKLYEDVELIHTPGHTEGSVCLFHKPLKA  LFTGDHLTMYESGMSIIEMYNHCSLPLQLESVERLIKLDFNWVIPDSNYLMASSISHDPS  SSSTSLLNLQTQQSIFGYKDKVKDFEKTQLRIPVSFRKKGINLQMMASGKTPGLTQEAND  CTYEANIDRDNNNTDVFDDMKQRFLAFKRLKYMDNLEHYKKLADAQAPKFLVIACADSRV  CPSAVLGFQPGEAFTVRNIANLVPPYESGPTETKAALQFSVNTLEVENILVIGHSRCGGI  QALMGMEEVDSRSFIHNWVIVGKKAKESTKAVASNLHFDHQCQHCEKTSINHSLERLLGY  PWIEEKVRKGSLSLHGGYYDFVNCTFEKWTVDYEGSRGSGIAVKNRCVLKISFRNAIPLI  ERAEWRWLALLEGKKVNRVLETEGIFGNQNREIEENRVLDCLENRVSETEGKAWGCLGNL  ALGLGVEENRVLGIEGMALDLLGNRVLETEENRV* |
| BrGLYII2 | Bra031460 | MASSSTSLKRREQQPMSREGDQLIVTPLGAGNEVGRSCVYMSFRGKTILFDCGINPAYSG  MAALPYFDEIDPSTIDVLLITHFHLDHAASLPYFLEKTTFKGRVFMTHATKAIYKLLLTD  YVKVSKVSVEDMLFDEHDINKSMDKIEVIDFHQTVEVNGIKFWCYTAGHVLGAAMFMVDI  AGVRILYTGDYSREEDRHLRAAELPQFSPDICIIESTSGVQLHQSRHIREKRFTDVIHST  VAQGGRVLIPAFALGRAQELLLILDEYWANHPDLHNIPIYYASPLAKKCMAVYQTYILSM  NDRIRNQFANSNPFVFKHISALNSIDDFRDVGPSVVMASPGGLQSGFSRQLFDIWCSDKR  NACIIPGYMVEGTLAKTIINEPKEVTLMNGLTAPLNMQVHYISFSAHADYAQTSTFLKEL  MPPNIILVHGEANEMMRLKQKLFTEFPDGNTRIMNPKNCESVEMYFNSEKMAKTIGRLAE  KTPDVGDSVSGILVKKGFTYQIMAPDDLHVFSQLSTATVTQRITIPFSGAFGVITHRLGK  IFESVESSTDEETGLPALKVHERVTVKQESEKHISLQWSSDPISDMVSDSIVALVLNISR  EVPKIVAEEEVAVKSEEENGKKVEKVIYALLVSLFGDVKLGENGKLVISVDGNVAHLDKE  SGDVEGEHEGLKERVRVAFHRIQSAVKPIPLSAE* |
| BrGLYII3 | Bra026637 | MAIDCLVLGAGQEIGKSCVVVTINGKKIMFDCGMHMGCDDHNRYPDFSILSKSGDFDNTI  SCLIITHFHMDHVGALPYFTEVCGYNGPVYMSYPTKALSPLMLEDYRRIMVDRRGEEELF  TSAHIASCMNKVIPLDLKQTIQVDEDLQIRAYYAGHVLGAVMVYAKVGDAAIVYTGDYNM  TTDRHLGAAKIDRLQLDLLISESTYATTTRGSKYPREREFLQAVHKCVAGGGKALIPSFA  LGRAQELCMLLDDYWERMNIKVPIYFSSGLTIQANMYYKMLISWTSQNVKEKHVTHNPFD  FKNVKDFYRSLIHAPGPCVLFATPGMLCAGFSLEVFKHWAPSPLNLVALPGYSVAGTVGH  KLMSGKPTTVDLYNGTKVDVRCKIHQVAFSPHTDAKGIMDLTKFLSPKNIVLVHGEKPSM  MSLKDKITSELGIPCFVPANGETVSVASTTFVKANASDMFLKICSNPNFRFSNSSTQLRV  TDQRTADGVLVIEKSKKAKIVHQDEVSEVLHEKDHVVSLAYCCPVKVKGESDDVGLIKQL  SEKISETVSCADEIHESETCLQVGSFKGSLCLKEECVHRRGISSSCSEVKFLCCNWSVAD  LELGWGIINAMKQNL* |
| BrGLYII4 | Bra022836 | MQNISKASSAISFFRCSRNLASQPCVRQLHIRKGLVSRVMKLVSSPLRTLRGAGKSIRVS  SFCSVSSSISSLQIEMVPCLKDNYAYILHDEDTGTVGVVDPSEAEPVIDSLQRSGRNLTY  ILNTHHHYDHTGGNLELKDRYGAKVIGSALDRDRIPGIDIALKDGDKWMFAGHEVHVMDT  PGHTKGHISLYFPGSRAIFSGDTLFSLSCGKLFEGTPKQMLASLKKIISLPDDTSIYCGH  EYTLSNSKFALSIEPNNQVLQSYAAHVAELRKKKLPTIPTTLKMEKACNPFLRSSNTDIR  QALGISETADEAEALAIIREAKDNFKA* |
| BrGLYII5 | Bra000305 | MWLFSMPLKTLRGARKTLKVTHFCSISNMPSSLKIELVPCSKDSYAYLLHDEDTGTVGVV  DPSEAAPVIDALSRKNWNLTYILNTHHHDDHIGGNAELKARYGAKVIGSAVDKDRIPGID  ILLKDSDKWMFAGHEVRVIDTPGHTQGHISFYFPGSATIFTGDLIHSLSCGTLSEGTPEQ  MLSSLQKIVSLPDDTNIYCGRENTAGNIKFALSIEPKNETLHSYATRVAHLRSQGLPSIP  TTVKVEKACNPFLRTSSKEIRRSLSIPDSANEAEALRCIHRARDRF* |
| BrGLYII6 | Bra037715 | MPLKTLRGARKTLKITHFCSISNMPSSLKIELVPCSKDNYAYLLHDEDTGTVGVVDPSEA  APVIEALSRKNWNLTYILNTHHHDDHVGGNAELKAKYGAKVIGSALDKDRIPGIDIHLND  GDKWMFAGHEVRVLSTPGNTQGHISFYFPGSATIFTGDLLYSLSCGTISEGTPEQMLSSL  QKIVSLPDDTNIYCGRENTAGNLKFALSVEPKNETLQSYATRVAHLRSQGLPSIPTTVKL  EKECNPFLRTSSKDIRKSLSIPESATEAEALRRIQRARDRF* |
| BrGLYII7 | Bra004763 | MWLFSMPLKTLRGARKTLKITHFCSISNMPSSLKIELVPCSKENYAYILHDEDTGTVGVV  DPSEAAPVIEALSRKNWNLTYILNTHHHDDHIGGNAELKAKYGAKVIGSAVDKDRIPGID  ILLKESDKWMFAGHEVRVIDTPGHTQGHVSFYFPGSATVFTGDLIHSLSCGTLSEGTPEQ  MLSSFQKIVSLPDDTNIYCGRENTSGNVKFALSIEPKNETLRSYATRVAHLRSQGLPSIP  TTVKVEKACNPFLRTSSKEIRRSLNIPESANEAEALRRIHRARDRF* |
| BrGLYII8 | Bra018252 | MKLVSSPLRTLRGASKSIRVSNFCSVSNLSSLQIELVPCLNDNYAYILHDEDTGTVGVVD  PSEAEPVIESLQRSGRNLTYILNTHHHYDHTGGNLELKDRYGAKVIGSAVDRDRIPGIDI  ALKDGDKWMFAGHEVHVMDSPGHTKGHISLYFPGSRAIFTGDTLFSLSCGKLFEGTPKQM  LASLQRIISLPDDTSIYCGHEYTLSNSKFALSIEPNNEVLQSYAAHVAELRQKKLPTIPT  TVKMEKACNPFLRSSNTDIRRALGISETADDAEALGIIREAKDNFKA* |
| BrGLYII9 | Bra029872 | MKISHVPCLEDNYSYLIIDESTGDAAVVDPVEPEKVIKSAEQHSANIKFVLTTHHHWDHA  GGNEKMKQLVSGIKVYGGSLDKVKGCTDAVDNGDTLSLGQNINILALHTPCHTKGHISYY  VTGKDGETPAVFTGDTLFVAGCGEVFEGTAEQMHQSLCVTLASLPKPTQVYCGHEYTVKN  LEFALTVEPNNEKIQQKLSWARQQRQANLPTIPSTLEEELETNPFMRVNNPEIQEKLGCK  SPIDTLREIRNKKDQWRG* |
| BrGLYII10 | Bra039681 | MGSSSSSSSSSSSKLLFRQLFEKESSTFTYLLADVSHPDKPALLIDPVDKTVERDLKLIN  ELGLKLVYAMNTHVHADHVTGTGLLKTKVPGVKSVISKASGSKADMFLEPGDKVSIGDIY  LEVRATPGHTAGCVTYVTGEDADQPQPRMAFTGDAVLIRGCGRTDFQIFTLPKDTLIYPA  HDYKGYEVSTVGEEMEHNPRLTKDKETFKSIMSNLNLAYPKMIDVAVPANMVCGLQDLPP  QANL* |
| BrGLYII11 | Bra038629 | MKPAASLHGYPSSPIYFDARRPVPTPPSKMAAFSALSLSPYSFTFRQSSPVRSTVSCSVT  SPPASSGTSSSSSSSNKTPRRRSGRLEGAGKSMEDSVKRKMEQFYEGTDGPPLRVLPIGG  LGEIGMNCMLVGNYDRYILIDAGIMFPDYDDPGVQKIMPDTGFIRRWKHKIEAVVITHGH  EDHIGALPWVIPALDSNTPIFASSFTMELIKKRLKEHGIFVQSRLKTFNTRRRFMAGPFE  IEPITVTHSIPDCSGLVLRCADGNILHTGDWKIDEAPLDGKVFDREALEELSKEGVTLMM  SDSTNVLSPGRTTSEKVVADALVRNVMAAKGRVITTQFASNIHRLGSIKAAADLTGRKLV  FVGMSLRTYLEAAWKDGKAPIDPSSLVKVEDIEAYSPKDLLIVTTGSQAEPRAALNLASY  GSSHAFKLTKEDIILYSAKVIPGNESRVMKMMNRLADIGPKIVMGKNEMLHTSGHAYRGE  LEEVLKIVKPQHFLPIHGELLFLKEHELLGKSTGIRHTTVIKNGEMLGVSHLRNRRVLSN  GFSSLGRENLQLMYSDGDKAFGTASELCIDERLRISSDGIIVLSMEIMRPGASENTLKGK  IRITTRCMWLDKGRLLDALHKAAHAALSSCPVNCPLSHMERTVSEVLRKIVRKYSGKRPE  VIAIAMENPMAVRADEVSARMSGDPNLGSGVAALRKVVEGNHKRNRTKKAPSQEEAGEII  DSAGLLAEEGTASSTYTEGAEDVPVRSSSEESDDFWKSFINPSSPPSPDETKNVDKSPDA  ETKTEDSESSREEEDDDNTSDSQTKSSTKRVRRNKWKPEEVKKVIRMRGELHSRFQVVKG  RMALWEEISSNLSAEGINRSPGQCKSLWASLIQKYEECKADERSKTSWSHYEDMNSILSE  LDTPAPK* |
| BrGLYII12 | Bra009712 | MGTSVQVSPLCGVYNENPLAYLVSIDGFNFLLDCGWNDLFDPSLLEPLSRVASTVDAVLL  SHPDTLHLGALPYAMKQLGLSAPVYATEPVHRLGLLTMYDQYLSRKQVSDFDLFTLDDID  SAFQNVIRLTYSQNFHLSGKGEGIVIAPHVAGHMLGGSIWKITKDGEEVVYAVDYNHRKE  RHLNGTVLQSFVRPAVLITDAYNALYTNQTQSHHRDTEFLDTISKHLEVGGNVLLPVDTA  GRVLELLLILEQHWSQRAFSFPIYFLTYVSSSTIDYVKSFLEWMSDSISKSFETSRDNAF  LLRHVTLLINKTDLDNAPPGPKVVLASMASLEAGFARDIFVEWANDPRNLVLFTETGQFG  TLARMLQAAPPPKFVKVTMSKRVPLAGEELIAYEEEQNRLKREEALRASLVKEEETKASH  GPDDNSSEPMVIDTKTTHDVVGSHGPAYKDILIDGFVPPSSSIAPMFPFYDNTADWDEYG  EVINPDDYVIKDEDMDRGAMHAGGDVDGRLDEATASLMLDTRPSKVISNELIVTVSCSLV  KIDYEGRSDGRSIKSTIAHVSPLKLVLVHATAEATEHLKQHCLNSICPHVYAPKIEETID  VTSDLCAYKVQLSEKLMSNVIFKKLGDSEVAWVDSEVGKTESEKRCLQPMASAAAPHKPV  LVGDLKMQDFKQFLASKGVQVEFAGGGALRCGEYVTLRKVGPTGQKGGASGPQQILIEGP  LCEDYYKIRDYLYSQFYLL* |
| BrGLYII13 | Bra030931 | MGSSSSSSSKLLFRQLFEKESSTYTYLLADISHPDKPALLIDPVDKTVDRDLKLINELGL  KLVYAMNTHVHADHVTGTGLLKTKVPGVKSVISKASGSKADKFVEHGERVSIGDLYLEVR  ATPGHTAGCVTYVTGEGADQPQPRMAFTGDAVLIRGCGRTDFQGGCSDQLYESVHSQATH  DYKGFEVSTVGEEMQHNPRLTKDKETFKSIMSNLNLPYPKMIDVALPANMVCGLQDLPSQ  AN* |
| BrGLYII14 | Bra024757 | MGNLNLAVIIKNPGDSAQFLLEKQKQPPKFGDEAYDSYVDSNLWDLPSADLPSLEDGTRS  VNALSIAESCSEEIDLKNFDLDSTLIRLLASLGIEFSDVGEWSFVRYVVEPEFGPDSCVP  TCFLSGKLLDTDKSLQDNCKWMSMEACFDCLLDAKPGGDRVGPLVLLGLGDGSMKQKLAP  SLPVQEYPPGVMIVPMRSRTLKPFTTTNLVVFAPENVSVDDQERDFVIHGDALIVDPGCH  YKLHIELKKIADALPRKLIVFVTHHHRDHIGGLSAIQESNPDAILVAHVKTRNRIDGWSG  NYTPVSGGENIYVNGQSLTVIFAPGHTDGHMALLHNSTRSLIVGDHCVGKGSAFLDIRSG  GNMTEYFQTTYKFLELSPHVVIPMHGRVNLWPKHMLCGYLKNRRSREESILKATEDGAQT  LFDIVSNVYSKVDRSFWLAAASNVRLHIDNLAVENKLPEGFSIQKFKASCGFSFVVRWAA  GYIGSRIPFKINKPGLIMSVIAAGAGYFLLYTCKKKNTIES* |
| BrGLYII15 | Bra032436 | MQAISKVSSAASLFRCSTKLTSQPCMSQLSLRKGLASGVIKLFSSPLKTLCDAGRSVHVS  RFCSTSNISSSLQIELVPCLADNYAYILHDEETGTVGVVDPSEAVPVMNALKQNGQNLTY  ILNTHHHYDHTGGNLELKDKYGAKVVGSAVDSKRIPGIDIALKDGDKWEFAGHEVHVMET  PGHTIGHISFYFPGARAVFTGDTLFSLSCGKLFEGTPEQMLASLQRIVALPDDTSIYCGH  EYTLSNSKFALSIEPTNEVLQSYAAYVAELRGKKLPTIPTTVKMEKACNPFLRTGNMDIR  RVLGVPETADEAEALGVIRRAKDNFKA* |
