# Supplementary material for: Genome-wide analysis and expression profiles of glyoxalase gene families in Chinese cabbage (Brassica rapa L)
Source: PLoS One. 2018 Jan 11;13(1):e0191159. doi: 10.1371/journal.pone.0191159 (PMC5764358; doi:10.1371/journal.pone.0191159)
Supplement: S4 Table — (DOCX) [file pone.0191159.s006.docx]

**S4 Table. Percentage of similarities among all** **BrGLYI proteins in Chinese cabbage.**

|  | BrGLYI1 | BrGLYI10 | BrGLYI7 | BrGLYI12 | BrGLYI8 | BrGLYI16 | BrGLYI2 | BrGLYI3 | BrGLYI13 | BrGLYI4 | BrGLYI14 | BrGLYI15 | BrGLYI11 | BrGLYI6 | BrGLYI9 | BrGLYI5 |
| --- | --- | --- | --- | --- | --- | --- | --- | --- | --- | --- | --- | --- | --- | --- | --- | --- |
| BrGLYI1 | 100 | 96 | 97 | 96 | 91 | 83 | 83 | 88 | 87 | 86 | 70 | 63 | 77 | 5 | 73 | 80 |
| BrGLYI10 |  | 100 | 99 | 97 | 90 | 82 | 83 | 87 | 86 | 86 | 70 | 63 | 77 | 5 | 73 | 79 |
| BrGLYI7 |  |  | 100 | 97 | 90 | 82 | 83 | 87 | 86 | 86 | 70 | 63 | 77 | 5 | 72 | 79 |
| BrGLYI12 |  |  |  | 100 | 91 | 83 | 83 | 87 | 87 | 86 | 71 | 64 | 77 | 5 | 73 | 80 |
| BrGLYI8 |  |  |  |  | 100 | 82 | 82 | 86 | 86 | 86 | 70 | 63 | 78 | 5 | 72 | 79 |
| BrGLYI16 |  |  |  |  |  | 100 | 97 | 84 | 83 | 80 | 71 | 64 | 73 | 6 | 73 | 82 |
| BrGLYI2 |  |  |  |  |  |  | 100 | 84 | 84 | 81 | 71 | 64 | 73 | 6 | 73 | 81 |
| BrGLYI3 |  |  |  |  |  |  |  | 100 | 89 | 86 | 70 | 63 | 77 | 4 | 72 | 81 |
| BrGLYI13 |  |  |  |  |  |  |  |  | 100 | 96 | 71 | 64 | 77 | 4 | 72 | 81 |
| BrGLYI4 |  |  |  |  |  |  |  |  |  | 100 | 71 | 64 | 77 | 4 | 72 | 79 |
| BrGLYI14 |  |  |  |  |  |  |  |  |  |  | 100 | 90 | 64 | 2 | 65 | 70 |
| BrGLYI15 |  |  |  |  |  |  |  |  |  |  |  | 100 | 57 | 2 | 58 | 63 |
| BrGLYI11 |  |  |  |  |  |  |  |  |  |  |  |  | 100 | 23 | 88 | 71 |
| BrGLYI6 |  |  |  |  |  |  |  |  |  |  |  |  |  | 100 | 20 | 4 |
| BrGLYI9 |  |  |  |  |  |  |  |  |  |  |  |  |  |  | 100 | 72 |
| BrGLYI5 |  |  |  |  |  |  |  |  |  |  |  |  |  |  |  | 100 |
