# Supplementary material for: Genome-wide analysis and expression profiles of glyoxalase gene families in Chinese cabbage (Brassica rapa L)
Source: PLoS One. 2018 Jan 11;13(1):e0191159. doi: 10.1371/journal.pone.0191159 (PMC5764358; doi:10.1371/journal.pone.0191159)
Supplement: S5 Table — (DOCX) [file pone.0191159.s007.docx]

**S5 Table. Pairwise similarities among paralogous pairs of BrGLYII proteins in *B. rapa***

| BrGLYII | BrGLYII5 | BrGLYⅡ7 | BrGLYⅡ6 | BrGLYⅡ8 | BrGLYⅡ4 | BrGLYⅡ15 | BrGLYⅡ9 | BrGLYⅡ14 | BrGLYⅡ12 | BrGLYⅡ3 | BrGLYⅡ2 | BrGLYⅡ11 | BrGLYⅡ1 | BrGLYⅡ13 | BrGLYⅡ10 |
| --- | --- | --- | --- | --- | --- | --- | --- | --- | --- | --- | --- | --- | --- | --- | --- |
| BrGLYII5 | 100 | 98 | 97 | 91 | 87 | 86 | 79 | 50 | 24 | 38 | 29 | 7 | 36 | 74 | 74 |
| BrGLYⅡ7 |  | 100 | 96 | 91 | 86 | 86 | 79 | 50 | 24 | 38 | 29 | 7 | 36 | 74 | 75 |
| BrGLYⅡ6 |  |  | 100 | 90 | 86 | 86 | 80 | 50 | 24 | 38 | 29 | 8 | 36 | 74 | 75 |
| BrGLYⅡ8 |  |  |  | 100 | 94 | 91 | 81 | 50 | 24 | 38 | 28 | 7 | 36 | 74 | 74 |
| BrGLYⅡ4 |  |  |  |  | 100 | 93 | 76 | 50 | 24 | 38 | 29 | 8 | 36 | 70 | 70 |
| BrGLYⅡ15 |  |  |  |  |  | 100 | 76 | 50 | 24 | 38 | 29 | 8 | 37 | 70 | 71 |
| BrGLYⅡ9 |  |  |  |  |  |  | 100 | 49 | 23 | 38 | 29 | 7 | 35 | 73 | 73 |
| BrGLYⅡ14 |  |  |  |  |  |  |  | 100 | 24 | 32 | 27 | 8 | 37 | 49 | 48 |
| BrGLYⅡ12 |  |  |  |  |  |  |  |  | 100 | 34 | 33 | 10 | 17 | 23 | 23 |
| BrGLYⅡ3 |  |  |  |  |  |  |  |  |  | 100 | 47 | 12 | 28 | 37 | 38 |
| BrGLYⅡ2 |  |  |  |  |  |  |  |  |  |  | 100 | 10 | 20 | 28 | 28 |
| BrGLYⅡ11 |  |  |  |  |  |  |  |  |  |  |  | 100 | 5 | 7 | 7 |
| BrGLYⅡ1 |  |  |  |  |  |  |  |  |  |  |  |  | 100 | 36 | 36 |
| BrGLYⅡ13 |  |  |  |  |  |  |  |  |  |  |  |  |  | 100 | 96 |
| BrGLYⅡ10 |  |  |  |  |  |  |  |  |  |  |  |  |  |  | 100 |
