# Supplementary material for: Genome-wide analysis and expression profiles of glyoxalase gene families in Chinese cabbage (Brassica rapa L)
Source: PLoS One. 2018 Jan 11;13(1):e0191159. doi: 10.1371/journal.pone.0191159 (PMC5764358; doi:10.1371/journal.pone.0191159)
Supplement: S6 Table — (DOCX) [file pone.0191159.s008.docx]

**S6 Table. Tissue-specific expression of *BrGLYI* and *BrGLYII* family genes ^a^**

| **Gene**  **symbol** | **Locus**  **identifier** | **Callus** | **Root** | **Stem** | **Leaf** | **Flower bud** | **silique** |
| --- | --- | --- | --- | --- | --- | --- | --- |
| BrGLYΙ1 | Bra008491 | 49 | 0 | 0 | 0 | 34 | 22 |
| BrGLYΙ2 | [Bra006835](javascript:modalDialog('multiSearch.php?gene=Bra006835','select%20database',390,200)) | 36 | 1 | 2 | 98 | 50 | 11 |
| BrGLYΙ3 | [Bra005612](javascript:modalDialog('multiSearch.php?gene=Bra005612','select%20database',390,200)) | 85 | 18 | 13 | 23 | 98 | 84 |
| BrGLYΙ4 | Bra018692 | 0 | 0 | 0 | 0 | 0 | 0 |
| BrGLYΙ5 | Bra018654 | 48 | 48 | 67 | 39 | 71 | 79 |
| BrGLYΙ6 | Bra019830 | 0 | 2 | 7 | 3 | 2 | 5 |
| BrGLYΙ7 | Bra026138 | 431 | 153 | 1 | 0 | 30 | 35 |
| BrGLYΙ8 | [Bra011950](javascript:modalDialog('multiSearch.php?gene=Bra011950','select%20database',390,200)) | 0 | 0 | 0 | 0 | 1 | 1 |
| BrGLYΙ9 | Bra004214 | 35 | 36 | 50 | 194 | 57 | 59 |
| BrGLYΙ10 | [Bra016662](javascript:modalDialog('multiSearch.php?gene=Bra016662','select%20database',390,200)) | 322 | 2 | 0 | 0 | 8 | 0 |
| BrGLYΙ11 | Bra016811 | 48 | 124 | 261 | 157 | 401 | 182 |
| BrGLYΙ12 | Bra026768 | 0 | 37 | 0 | 0 | 0 | 0 |
| BrGLYΙ13 | Bra031589 | 0 | 0 | 0 | 0 | 0 | 1 |
| BrGLYΙ14 | Bra032415 | 216 | 8 | 12 | 40 | 77 | 51 |
| BrGLYΙ15 | Bra015511 | 392 | 7 | 8 | 14 | 61 | 38 |
| BrGLYΙ16 | [Bra002767](javascript:modalDialog('multiSearch.php?gene=Bra002767','select%20database',390,200)) | 16 | 19 | 9 | 33 | 36 | 27 |
| BrGLYⅡ1 | Bra011454 | 14 | 3 | 2 | 3 | 10 | 9 |
| BrGLYⅡ2 | Bra031460 | 8 | 17 | 28 | 15 | 12 | 17 |
| BrGLYⅡ3 | Bra026637 | 1 | 2 | 3 | 2 | 1 | 1 |
| BrGLYⅡ4 | Bra022836 | 2 | 12 | 17 | 19 | 6 | 6 |
| BrGLYⅡ5 | Bra000305 | 91 | 18 | 9 | 13 | 25 | 15 |
| BrGLYⅡ6 | Bra037715 | 63 | 15 | 13 | 6 | 17 | 56 |
| BrGLYⅡ7 | Bra004763 | 9 | 12 | 7 | 6 | 9 | 17 |
| BrGLYⅡ8 | Bra018252 | 3 | 3 | 8 | 23 | 5 | 5 |
| BrGLYⅡ9 | Bra029872 | 89 | 40 | 34 | 32 | 77 | 70 |
| BrGLYⅡ10 | Bra039681 | 91 | 24 | 23 | 6 | 133 | 37 |
| BrGLYⅡ11 | Bra038629 | 8 | 5 | 45 | 54 | 13 | 8 |
| BrGLYⅡ12 | Bra009712 | 9 | 24 | 42 | 18 | 13 | 14 |
| BrGLYⅡ13 | Bra030931 | 16 | 8 | 10 | 5 | 157 | 65 |
| BrGLYⅡ14 | Bra024757 | 2 | 5 | 4 | 4 | 5 | 8 |
| BrGLYⅡ15 | Bra032436 | 3 | 2 | 5 | 5 | 1 | 1 |

**^a^** The gene expression levels were calculated by using fragments per kilobase per million (FPKM) measure.
